# Enteric Nervous System Damage by Food Contaminants: A Pathway to Neurodegeneration?

**DOI:** 10.1111/1541-4337.70448

**Published:** 2026-04-09

**Authors:** Helena Ramos, Ana Margarida Araújo, Isabel M. P. L. V. O. Ferreira, Miguel A. Faria

**Affiliations:** ^1^ LAQV‐REQUIMTE, Laboratory of Bromatology and Hydrology, Faculty of Pharmacy University of Porto Porto Portugal

## Abstract

The enteric nervous system (ENS), a key component of the gut–brain axis, has emerged as a critical player in the pathogenesis of Parkinson's disease (PD). It is the first neural system exposed to food contaminants (FCs)—a diverse group of ubiquitous toxic compounds fortuitously present in food derived from production, processing, storage, or environmental contamination. Emerging evidence suggests that FCs may initiate or amplify neurodegenerative processes, yet their effects on the ENS and their impact in gut‐to‐brain communication remain insufficiently characterized. This systematic review synthesizes current evidence on FCs‐induced effects on the ENS and its involvement in mediating neurotoxicity from dietary toxicants exposure. Following PRISMA guidelines, 67 studies were included pertaining to cellular or mammalian experimental models exposed to FCs via enteral routes, reporting ENS‐related outcomes or studying vagal involvement in modulating FC toxicity. The main FCs evaluated were pesticides, toxins, bisphenols, acrylamide, manganese, and micro‐/nanoplastics. Across studies, FCs consistently induced neurochemical remodeling of the ENS, activation of enteric glia, often coupled with intestinal alterations. Rotenone, paraquat, and polystyrene micro‐/nanoplastics promote α‐synuclein aggregation within the ENS and its vagal propagation to the brain. Vagotomy models confirmed that disrupting ENS–CNS communication attenuates FC‐related central neurotoxicity, supporting the involvement of food toxicants in gut‐to‐brain propagation of neurotoxic signals. These findings support the *body‐first* hypothesis of PD and position the ENS as a critical, yet underinvestigated interface in exposome‐related neurotoxicology. The review highlights research gaps and the need for improved models and long‐term, low‐dose studies reflecting realistic FC exposure.

## 1 Introduction

**FIGURE 1:**
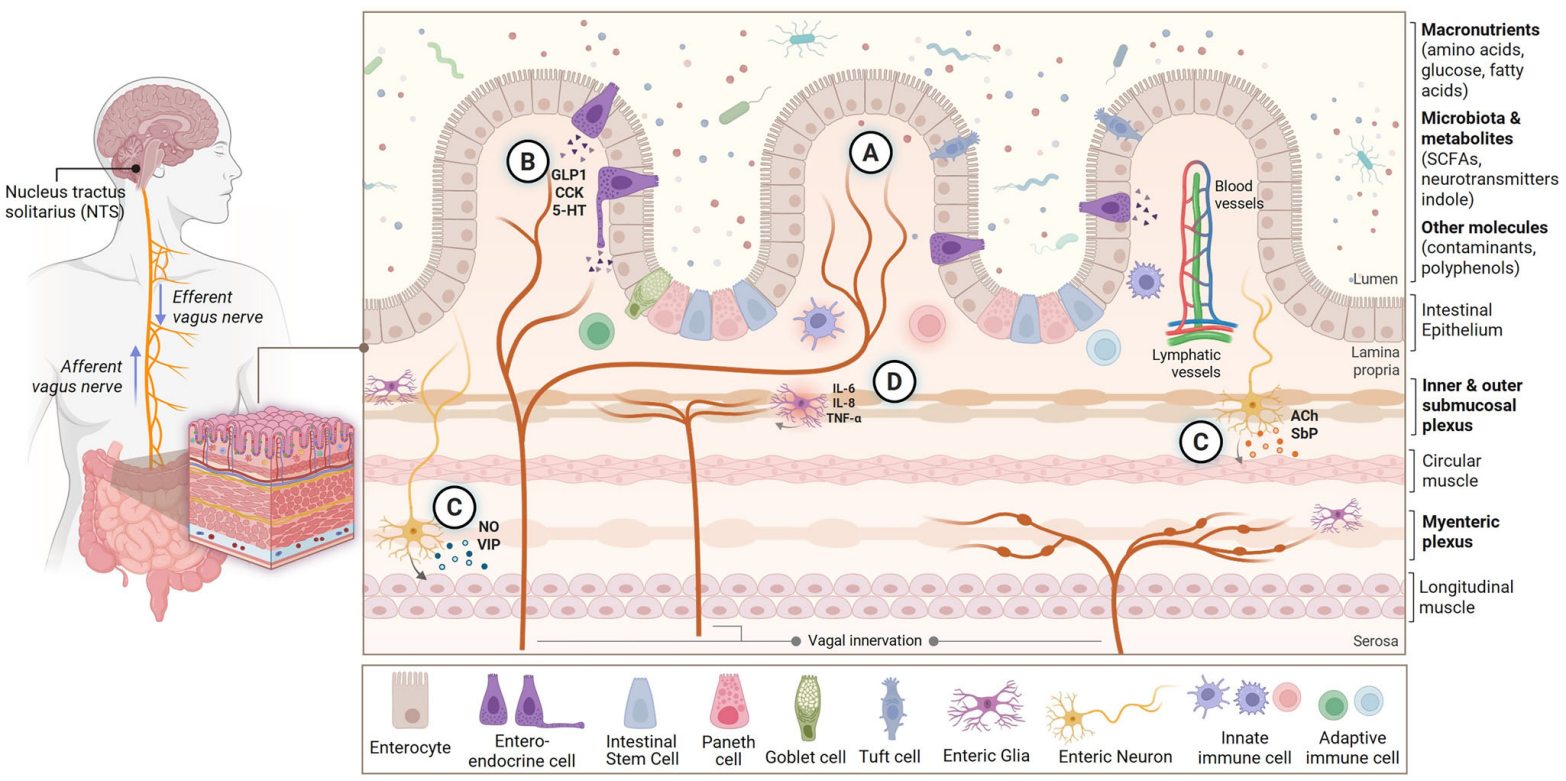
The enteric nervous system (ENS) as an interface between the gut and the brain. Schematic representation of the ENS within the gut wall and its integration in the gut–brain axis. The ENS comprises two major plexuses: the submucosal and myenteric plexuses, located respectively within the submucosa and between the circular and longitudinal muscle layers. These plexuses contain enteric neurons and glia that regulate motility, secretion, and barrier integrity, while maintaining bidirectional communication with the central nervous system via the vagus nerve. (A) Intrinsic sensory neurons detect luminal cues, including nutrients, microbial metabolites, and food contaminants. (B) Enteroendocrine cells release hormones such as glucagon‐like peptide 1 (GLP‐1), cholecystokinin (CCK), and serotonin (5‐HT), modulating vagal afferent activity. (C) Secretomotor and inhibitory motor neurons control gut contractility through neurotransmitters such as acetylcholine (ACh), substance P (SbP), vasoactive intestinal peptide (VIP), and nitric oxide (NO). (D) Enteric glia and immune cells coordinate local inflammatory responses through cytokines such as IL‐6, IL‐8, and TNF‐α. Together, these interconnected pathways regulate gastrointestinal homeostasis and constitute a primary route through which luminal exposures, including dietary food contaminants, can influence brain function.

The enteric nervous system (ENS), along with the endocrine system, immune system, and the gut microbiota constitutes the gut–brain axis (GBA), a dynamic and bidirectional network that regulates digestion, immune function and stress responses (Holzer and Farzi 2014; Carabotti et al. 2015; Chakrabarti et al. 2022). It comprises an autonomous network of 200–600 million neurons embedded within the gut wall (Sharkey and Mawe 2023) that governs key physiological processes, including motility, secretion, mucosal blood flow, and maintenance of epithelial barrier integrity, while also serving as a direct communication hub with the brain (Carabotti et al. 2015; Fung and Vanden Berghe 2020; Spencer and Hu 2020; Sharkey and Mawe 2023; Gonzales and Gulbransen 2025). Structurally, it is organized into two major plexuses: the myenteric plexus (MP), located between the circular and longitudinal muscle layers and essential for motility, and the submucosal plexus (SP), located closer to the lumen and involved in secretory and absorptive functions (Figure 1; Montanari et al. 2023; Sharkey and Mawe 2023). In humans and other large mammals, the SP is further subdivided into an inner submucosal plexus (ISP), adjacent to the muscularis mucosae, and an outer submucosal plexus (OSP), near the circular muscle layer (Montanari et al. 2023; Sharkey and Mawe 2023). Within these plexuses, the ENS comprises a highly diverse population of unmyelinated neurons, estimated to include at least 20 functional subtypes utilizing over 30 neurotransmitters, along with a dense network of enteric glia that support both neural and epithelial function (Bubeck, Becker, and Patankar 2023). Functionally, ENS neurons fall into three main categories: intrinsic primary afferent neurons (IPANs), interneurons, and motor neurons (Fleming et al. 2020; Montanari et al. 2023). These are further classified based on morphology (Dogiel types), electric, neurochemical, and functional‐related properties (Sharkey and Mawe 2023).

IPANs, often found in the MP and to a lesser extent in the SP, detect mechanical and chemical changes in the lumen, activating interneuronal pathways that organize ascending excitatory and descending inhibitory circuits essential for peristaltic reflexes, which in turn drive motor neurons targeting smooth muscle, secretory epithelium, or blood vessels (Fleming et al. 2020; Montanari et al. 2023; Sharkey and Mawe 2023). Excitatory motor neurons are typically cholinergic (acetylcholine [ACh]) and may corelease substance P (SbP, a neuromodulator peptide), stimulating muscle contraction (Fung and Vanden Berghe 2020). Inhibitory motor neurons produce nitric oxide (NO) via neuronal nitric oxide synthase (nNOS), and commonly coexpress vasoactive intestinal peptide (VIP) and pituitary adenylate cyclase‐activating peptide (PACAP), facilitating smooth muscle relaxation and vasodilation (Fung and Vanden Berghe 2020). In addition to regulating gut motility, the MP engages in reciprocal neuron‐macrophage signaling circuits through cholinergic and dopaminergic pathways that are central to this immunomodulatory function (Yoo and Mazmanian 2017; Wang et al. 2022). By contrast, the SP controls epithelial secretion, local blood flow, and barrier maintenance (Yoo and Mazmanian 2017; Sharkey and Mawe 2023). Its neurons modulate mucosal immunity by triggering goblet and Paneth cell secretions, facilitating antigen translocation to dendritic cells, and integrating signals from immune mediators such as mast cell‐derived serotonin (Yoo and Mazmanian 2017). The ENS utilizes a broad spectrum of neurotransmitters and modulators allowing it to dynamically coordinate motility, secretory activity, immune responses, and vascular tone across the gastrointestinal tract in a highly integrated and adaptive manner across different gut regions (Fleming et al. 2020; Montanari et al. 2023; Sharkey and Mawe 2023).

Enteric glia outnumbers neurons by approximately three‐ to fivefold, these cells distributed not only within ganglia but also along nerve fibers and in the lamina propria (Fleming et al. 2020; Sharkey and Mawe 2023). Resembling astrocytes, enteric glial cells (EGCs) regulate gut physiology by modulating neurotransmission, maintaining epithelial barrier integrity, and mediating immune responses (Sharkey and Mawe 2023). Their processes often extend beyond enteric ganglia into the surrounding muscle and mucosal layers. In response to inflammation or injury, EGCs become reactive, a process known as enteric gliosis, characterized by proliferation and release of pro‐inflammatory mediators (Bubeck, Becker, and Patankar 2023; Sharkey and Mawe 2023). The ENS also communicates with the CNS by detecting luminal chemical and mechanical stimuli through intrinsic sensory neurons and interneurons, which relay information to the brain via vagal and spinal afferents (Carabotti et al. 2015; Fung and Vanden Berghe 2020). These pathways transmit signals to the nucleus tractus solitarius (NTS), allowing the CNS to respond to gut perturbations, including inflammatory or pathogenic insults. Disruption of this axis contributes not only to GI conditions such as irritable bowel syndrome (IBS), but also to neurological and neurodegenerative disorders (NDs; Bubeck, Becker, and Patankar 2023; Montanari et al. 2023; Sharkey and Mawe 2023). Among these, Parkinson's disease (PD) provides the strongest evidence for ENS involvement in central pathology (Svensson et al. 2015; Stokholm et al. 2016; Kim et al. 2019; Borghammer et al. 2022).

**FIGURE 2:**
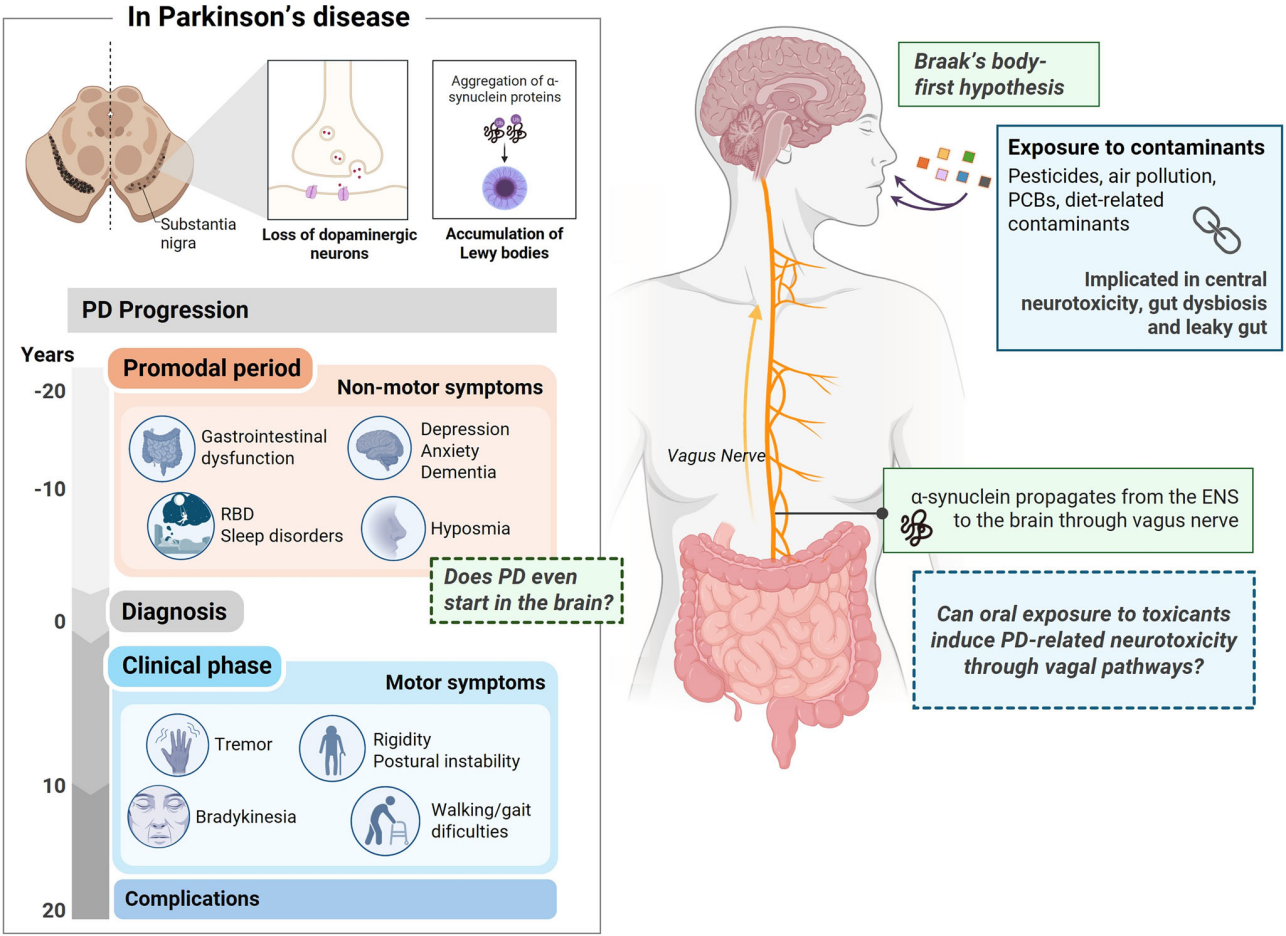
Gut–brain axis and Parkinson's disease: the role of the ENS and environmental/dietary toxicants. Parkinson's disease (PD) is characterized by dopaminergic neurodegeneration in the substantia nigra and the accumulation of α‐synuclein (α‐syn) aggregates, leading to hallmark motor symptoms, but also preceded by decades of nonmotor features such as gastrointestinal dysfunction, hyposmia, and sleep disorders. Increasing evidence supports Braak's body‐first hypothesis, which proposes that PD pathology may originate in peripheral sites such as the enteric nervous system (ENS) or olfactory bulb, before propagating to the brain via autonomic pathways, particularly the vagus nerve. Environmental and dietary toxicants, including pesticides, air pollutants, polychlorinated biphenyls (PCBs), have been implicated as major risk factors for PD. Within the gut, exposure to such toxicants may promote microbiota dysbiosis, increased intestinal permeability, inflammation, and enteric neurotoxicity, ultimately facilitating α‐syn misfolding and spread to the central nervous system (CNS). This framework highlights the ENS as both a vulnerable target of food contaminants and a potential initiation site for PD‐related neurodegeneration.

PD is a progressive neurodegenerative disease characterized by dopaminergic neuronal loss in the substantia nigra and the accumulation of misfolded α‐synuclein (α‐syn) aggregates, leading to hallmark motor symptoms such as tremor, bradykinesia, and rigidity (Dugger and Dickson 2017; Balestri et al. 2024; Dorsey and Bloem 2024; Figure 2). Beyond the classical motor symptoms, many patients experience prodromal nonmotor features, including constipation, anosmia, and autonomic dysfunction, often appearing decades before diagnosis (Braak, Del Tredici, et al. 2003a; Kalia and Lang 2015; Tansey et al. 2022). PD is also one of the fastest growing brain disorder worldwide, with a 61% increase in age‐standardized prevalence and an approximately 10% increase in age‐standardized disability‐adjusted life years (DALYs) between 1990 and 2021, a trend that cannot be fully explained by population aging or improved diagnostic practices (G. B. D. Dementia Forecasting Collaborators 2022; Ben‐Shlomo et al. 2024; G. B. D. Nervous System Disorders Collaborators 2024; Dorsey and Bloem 2024). Increasing evidence indicates that PD is a multifactorial disorder arising from complex interactions between genetic susceptibility, ageing, and environmental exposures (Morris et al. 2024). Environmental contributors, including certain pesticides, industrial solvents like trichloroethylene, and air pollution, are increasingly recognized as major drivers of PD risk (Borghammer et al. 2022; Ben‐Shlomo et al. 2024; Dorsey and Bloem 2024; Dorsey et al. 2024). Agricultural workers chronically exposed to organophosphates and other pesticides exhibit significantly higher PD incidence, and long‐term exposure to urban air pollution has also been associated with elevated risks of NDs (Shan et al. 2023). These observations support Braak's “body‐first” hypothesis, which proposes that PD pathology may originate outside the brain, with certain toxicants triggering α‐syn misfolding in peripheral neurons, particularly within the ENS or olfactory bulb, initiating a cascade that ascends toward the brain via autonomic pathways such as the vagus nerve (Braak, Rub, et al. 2003b; Borghammer et al. 2022; Velucci et al. 2025). Clinical and pathological evidence supports this framework: aggregated and phosphorylated α‐syn has been detected in the myenteric and SPs of PD patients years before disease onset (Braak et al. 2006; Beach et al. 2010; Stokholm et al. 2016), and large epidemiological cohorts suggest that truncal vagotomy reduces PD risk (Borghammer et al. 2015; Svensson et al. 2015; B. Liu, Fang, et al. 2017). While not definitive, these findings indicate that peripheral sites such as the ENS may be critical initiation points for neurodegeneration.

Preclinical PD models frequently rely on pesticides such as rotenone or paraquat to induce dopaminergic neurodegeneration and α‐syn pathology, phenotypes that are relevant for PD research (Lal et al. 2024). These toxicants induce gastrointestinal inflammation, enteric neurodegeneration, and α‐syn aggregation that subsequently propagate to the brain via the vagus nerve, recapitulating hallmark PD features consistent with transneuronal disease initiation (i.e., the progressive spread of pathological proteins or signals across neuron's synapses, leading to stepwise neurodegeneration; Kim et al. 2019). While these models often employ high doses or exposure routes not reflective of typical human exposure (i.e., osmotic pumps, intraperitoneal route), they consistently demonstrate that gut‐mediated pathways can participate in neurodegenerative processes triggered by neurotoxicants (Cresto et al. 2023; Montanari et al. 2023).

Nevertheless, the contribution of chronic, cumulative exposure to neurotoxicants remains insufficiently characterized, particularly for low‐dose mixtures acting over long latency periods, a scenario consistent with human dietary exposure (Deepika et al. 2020). Diet is the primary route of exposure to a broad range of food contaminants (FCs), such as heavy metals, pesticides, processing‐related compounds (e.g., acrylamide), mycotoxins, plasticizers (e.g., bisphenols, phthalates), and emerging environmental pollutants including microplastics and nanoplastics (MNPs). Unlike acute poisoning, consumers are chronically exposed to low levels of various FCs through daily food intake; even individuals with balanced, healthy diets ingest trace levels of these contaminants (Ramos et al. 2024). Although confounding variables and exposure heterogeneity complicate causal inference, the dietary exposome holds significant potential as a modifiable risk factor for neurodegeneration (Shan et al. 2023; Dorsey and Bloem 2024; Lefevre‐Arbogast, Chaker, et al. 2024). Several of the referred FCs (e.g., heavy metals, acrylamide, bisphenols, and mycotoxins) have been reported to exert neurotoxic effects and contribute to intestinal barrier disruption, gut inflammation, and microbiota dysbiosis processes that are mechanistically relevant to GBA signaling (Cresto et al. 2023; Kulcsarova et al. 2023; Porru et al. 2024; Yang et al. 2024).

Despite being the first and most direct neural structure exposed to ingested contaminants, the ENS remains a critical yet underexplored target of FC‐induced neurotoxicity. Research to date has been mainly focused on how FCs shape gut microbiota and influence brain health, overlooking the ENS as a direct communication channel to the brain. Thus, this systematic review synthesizes the current evidence on the effects of FCs in the ENS via dietary‐relevant exposure routes and clarifies the ENS's potential role as mediator of contaminant‐induced central neurotoxicity.

## 2 Methods

### 2.1 Search Strategy

**FIGURE 3:**
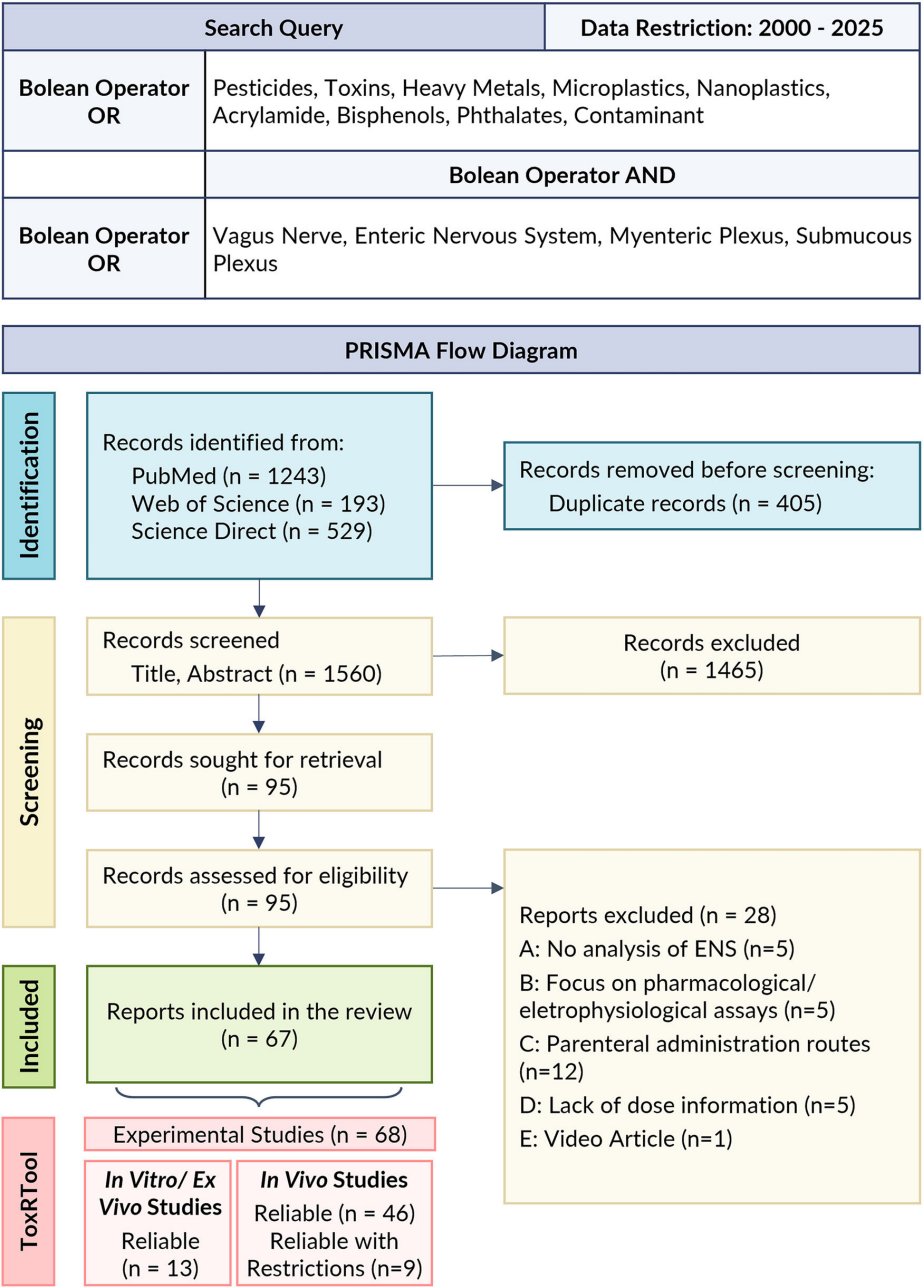
PRISMA flow diagram summarizing the systematic literature search and selection process. Included studies were further assessed for methodological reliability using ToxRTool, with separate scoring for in vitro/ex vivo and in vivo models (Page, McKenzie, et al. 2021a; Page, Moher, et al. 2021b).

The search strategy included keywords to capture relevant FCs classes and ENS‐related terms based on preliminary results. Initial searches including specific contaminant classes, namely, perfluoroalkyl and polyfluoroalkyl substances (PFAS), nitrosamines, heterocyclic aromatic amines, dioxins, and flame retardants yielded no eligible studies and were therefore excluded from the final query. Regarding the ENS, the term “gut–brain axis” was initially explored but was excluded from the final query due to its frequent association with studies focusing on microbiota‐related dysbiosis and CNS outcomes, without addressing ENS role. Figure 3 depicts the final query applied across PubMed, Web of Science, and Science Direct. For PubMed, Medical Subject Headings (MeSH) terms and free‐text keywords were employed to enhance sensitivity and specificity. Full search syntax, including database‐specific terms and filters, is available in Table S1.

### 2.2 Eligibility Criteria

This systematic review follows the Preferred Reporting Items for Systematic Reviews and Meta‐Analyses (PRISMA) guidelines (Page, McKenzie, et al. 2021a; Page, Moher, et al. 2021b). Eligibility criteria for study selection were defined using the PECO (population, exposure, comparator, outcomes) framework as depicted in Table 1. Eligible studies must examine the effects of FCs on the ENS using in vitro or ex vivo models, through enteral FC exposure in animal models, or by investigating the ENS's role in FC‐induced neurotoxicity.

**TABLE 1:** PECO eligibility criteria for study inclusion.

PECO format	Inclusion criteria	Exclusion criteria
Population	‐ Mammalian in vitro, ex vivo, and in vivo models	‐ Nonmammalian vertebrate models (e.g., *Cyprinus carpio*, *Danio rerio*)
Exposure	‐ Exposure to food contaminants (e.g., heavy metals, pesticides, mycotoxins, acrylamide, bisphenols, micro‐/nanoplastics), either alone or in combination with dietary components (e.g., lectin, inulin) or relevant elements for ND pathology (e.g., α‐synuclein) ‐ Enteral/gastrointestinal routes in animal models	‐ No exposure to food contaminants (e.g., pure physiology) or exposure to nontarget substances (e.g., drugs or agonists) ‐ Parenteral administration routes (e.g., intraperitoneal or intravenous) in animal models due to lack of relevance to human dietary exposure ‐ Lack of dose information (e.g., missing mg/kg/day or nonconvertible concentrations from water and diet intake)
Comparison	‐ Control group (e.g., vehicle or group without FCCs)	‐ No control group
Outcomes	‐ Structural, functional, molecular, or biochemical endpoints in the ENS (e.g., neuronal viability, neurotransmitters, histology) and the role of ENS and vagal communication in modulating FCC‐toxicity (e.g., models involving vagotomy procedures)	‐ No analysis of ENS (e.g., studies focused exclusively on CNS, microbiota or unrelated organ systems) ‐ Pharmacological or electrophysiological ENS assessment

Given the complex physiology of the human ENS, only studies conducted in mammalian models were included. Eligible studies must be original, peer‐reviewed research articles published in English between January 2000 and January 21 2026.

### 2.3 Selection Process

All retrieved records were imported into EndNote 21.5 (Clarivate Analytics, Philadelphia, PA, USA) for reference management. Duplicate entries were identified and removed using both automated detection and manual verification. Only entries from 2000 onward were retained. Papers published in any other language than English, nonprimary research articles, such as review articles, opinion articles, commentaries, book chapters or conference communications, abstracts only, notes, theoretical studies, editorials, in silico analyses, preprints or video articles were excluded.

Records title, abstracts, or full‐texts were then reviewed to exclude studies that were (1) focused primarily on environmental or vertebrate ecotoxicology, (2) investigated chemical compounds outside the scope of the defined FC classes, (3) solely studied FCs effects on gut microbiota or nonrelevant systems such as the respiratory, cardiovascular, or CNS without ENS endpoints, or (4) were ENS physiological studies without target compound exposure, or with an electrophysiology or pharmacology focus. Given the dietary relevance of this review, for the final selection, full‐texts were reviewed to exclude (5) studies employing parenteral administration routes (e.g., osmotic pumps, intraperitoneal, or intravenous injections), often used in models of PDs as these methods bypass the GI tract and therefore do not reflect realistic human exposure scenarios to diet‐related contaminants, and (6) articles lacking explicit dosing information (e.g., without standardized units such as mg/kg body weight/day) or fail to provide sufficient data to convert dietary or drinking water concentrations (e.g., ppm or mg/kg feed) into actual intake doses. Full‐text screening was carried out independently by two reviewers. Discrepancies were resolved by consensus, with the involvement of a third reviewer.

### 2.4 Data Collection Process and Synthesis

Data were collected from the studies’ main text, tables, figures, and supporting information. The following variables were collected: bibliographic information (title, authors, year of publication); contaminant characteristics (chemical name, chemical class, purity, source, and methods used for micro‐/nanoplastics structural characterization); model specifications (species and strain, details on in vitro cell lines or primary cells isolated from animals); and exposure parameters, including dose, administration route, frequency, and duration. Methodological descriptors such as assay type, analytical techniques, control group design, in vitro models, and IC_50_ values from cytotoxicity assays were collected when reported. Nomenclature of origin research articles was maintained whenever possible.

Following the PRISMA glossary of terms, a *study* was defined as the evaluation of a given FC within the same experimental design or animal cohort, even when data were distributed across multiple reports. Individual reports may also include more than one study if they assessed distinct FCs or employed different biological systems (in vitro and in vivo). For in vivo studies, variations such as exposure duration or animal characteristics (e.g., α‐synuclein‐transfected or vagotomized models) were considered as treatment groups within the same study. The same principle was applied to in vitro and ex vivo experiments, except when biologically distinct preparations were used (e.g., EGCs vs. primary mixed cultures), which were treated as separate case studies. Findings from in vitro and ex vivo studies were categorized based on the main cellular mechanisms involved, including oxidative stress, mitochondrial dysfunction, apoptosis, inflammation, autophagy, and others. For in vivo studies, ENS‐related outcomes were extracted, and when available, additional data on CNS effects, intestinal function, and other parameters such as microbiota composition were also collected. Outcomes on the ENS included both direct assessments (e.g., immunohistochemical or molecular analysis of ENS markers) and indirect evidence from vagotomy experimental procedures. Some research articles focused only on the evaluation of ENS, frequently reuse of the same animal cohorts and exposure protocols across multiple studies, differing mainly in specific neuropeptides or neurotransmitters and the gastrointestinal regions examined. Relevant data were extracted from the text of the paper, tables or graphs using WebPlotDigitalizer (version 5.2, Automeris). To enable consistent cross‐study comparison and synthesis of FC‐induced neurochemical alterations in the ENS, a secondary data processing step was applied. Neuronal subpopulations were found predominantly quantified using immunohistochemistry or immunofluorescence, with changes typically expressed as the proportion of marker‐positive neurons relative to the total neuronal population. To standardize outcomes and support comparative analysis, these values were recalculated as fold differences between average treatment and control groups. Square heatmap matrix plots were created in Python 3.12 to display fold‐change values in immunoreactivity relative to control animals, where color hue encodes the direction and magnitude of change (blue for downregulation, red for upregulation), and square size reflects the relative abundance of the neuronal subpopulation under analysis within the respective gut region and plexus under control conditions.

### 2.5 Data Reliability Assessment

The methodological quality of the included studies was assessed using the Toxicological data Reliability Assessment Tool (ToxRTool), developed by the European Commission's Joint Research Centre (Schneider et al. 2009). ToxRTool provides a standardized framework for assessing the reliability of toxicological data, comprising 18 criteria for in vitro and 21 criteria for in vivo studies. Ex vivo studies were classified and evaluated using the in vitro criteria. Although some criteria is ultimately left to the evaluator's judgment, the ARRIVE guidelines were consulted to ensure scoring consistency (Percie du Sert et al. 2020). Studies were subsequently categorized according to the Klimisch classification system: Category 1 (reliable without restrictions) was assigned to in vitro studies scoring between 15–18 and in vivo studies scoring between 18–21; Category 2 (reliable with restrictions) to in vitro studies scoring 11–14 and in vivo studies scoring 13–17; and Category 3 (not reliable) was assigned to studies scoring below 11 (in vitro) or below 13 (in vivo), or to any study that failed to meet one or more of the designated *red criteria*, regardless of total score.

## 3 Results

### 3.1 Study Selection

The PRISMA flowchart in Figure 3 overviews the study selection process for this systematic review. Database searches initially yielded 1965 records, of which 1560 remained after duplicate removal. Titles and abstracts screening excluded 1465 records based on predefined criteria, including automatic exclusions such as language or publication type, environmental‐related studies, use of nontarget compounds, focus on nonrelevant systems (e.g., respiratory, cardiac, or central nervous systems), studies limited to physiological or pharmacological exploration of the vagus nerve or ENS without toxicological exposure, and other unrelated topics. After initial screening, 95 articles were retained for full‐text evaluation, of which 28 were excluded for not meeting the criteria. In total, 67 reports were included in this systematic review and subsequently assessed for reliability using ToxRTool.

### 3.2 Methodological Quality Study Assessment

The methodological quality of the included reports was evaluated separately for in vitro and in vivo studies through using ToxRTool (Schneider et al. 2009). All mandatory criteria were fulfilled across the evaluated studies. In vitro/ex vivo studies yielded ToxRTool scores ranging from 15 to 17, with all 13 studies classified as reliable without restrictions (Category 1; Table S2). Fifty‐five in vivo studies scored between 16 and 21, with 46 studies considered reliable (Category 1) and nine studies deemed reliable with restrictions (Category 2; Table S3). For porcine studies, which reported various outcomes from the same experimental design in separate publications (e.g., different enteric neuronal subpopulations or GI tract regions examined across multiple articles), quality appraisal was performed collectively per experimental setup to avoid redundancy and ensure consistency.

### 3.3 Synthesis of Results

Data from the included studies are summarized in tables and discussed in the following sections. For clarity, studies outcomes were grouped into three categories: (i) in vitro and ex vivo models, summarized in Table 2; (ii) in vivo studies focusing on local ENS outcomes, addressing neuronal and glial changes, neurochemical remodeling, gut barrier integrity, and GI inflammation or histopathology, presented in Table 3; and (iii) in vivo studies evaluating ENS–CNS interactions, including assessments of vagal communication, α‐synuclein pathology, and central nervous system alterations, compiled in Table 4.

**TABLE 2:** Summary of experimental conditions, methodological approaches, and outcomes of FCs exposure using in vitro and ex vivo cellular models of the enteric nervous system.

Study	Compound and treatment regimen	Biological model	Methods	Molecular and cellular outcomes
**Pesticides**				
Diss et al. (2016)	Paraquat Conditions: ‐ PQ (20 mM) ‐ Untreated control Duration: 60 min	Distal colon segments (C57BL/6 male mice, 3–4‐month‐old)	Colonic motility: pellet motility tracking, colonic migrating motor complexes (CMMC) recordings, electrical field stimulation (EFS) Oxidative stress (MDA assay), protein analysis (WB: TNFα, complex I), enzymatic activity (complex I assay).	↓Colonic transit: ↓ CMMC amplitude and coordination, ↓ pellet motility; ↑ resting tension, ↓ EFS‐induced relaxation Impaired nitrergic signaling: ↓ NO release n.s. alterations in oxidative stress (MDA), inflammation (TNFα), or mitochondrial complex I activity
Pan‐Montojo et al. (2012)	Rotenone Conditions: ‐ 10–100 nM ‐ Untreated control Duration: 24–48 h	Primary enteric neurons expressing mCherry‐α‐syn (C57BL/6J mice, 1–6‐day‐old) Coculture of mCherry–α‐synuclein expressing enteric neurons and non‐neuronal cells	Genetic manipulation: Lentiviral transduction with mCherry‐α‐syn Immunohistochemistry: α‐syn, TH, PGP9.5 Live‐cell imaging	↑ α‐synuclein release from enteric neurons Coculture: Non‐neuronal cells accumulated α‐syn inclusions in a dose‐dependent manner (∼2–5×) Inclusions localized to smooth muscle actin‐positive cells, showing uptake by non‐neuronal intestinal cells No inclusions were observed in non‐neuronal cells cultured without neurons (neuronal origin confirmed)
Pan‐Montojo et al. (2012)	Rotenone Conditions: ‐ 0.1–5 µM ‐ Untreated control Duration: 24–48 h	Primary sympathetic neurons (C57BL/6J mice, 1–6‐day‐old)	Immunohistochemistry/WB: TH, β‐tubulin, α‐syn Exosomes: TEM with immunogold labeling; flow cytometry	↑ Extracellular α‐synuclein release detected in exosomal and nonexosomal fractions; rotenone increased exosome number/field (stimulated exocytosis) ROT increased number of exosomes per field, suggesting stimulated exocytosis.
Pan‐Montojo et al. (2012)	Rotenone Conditions: ‐ 10–100 nM ‐ Untreated control Duration: 24–48 h	Enteric–sympathetic cocultures (mCherry–α‐synuclein expressing enteric neurons with sympathetic TH+ neurons)	Cell culture: Enteric neurons plated on PLL‐coated beads; cocultured with sympathetic neurons either directly or via Campenot chambers (directional neurite growth Immunohistochemistry: α‐syn, TH, PGP9.5	Cell‐to‐cell transference: Sympathetic neurons internalized mCherry–α‐synuclein released by enteric neurons. Axonal transport: α‐syn was retrogradely transported from neurites to soma, where it accumulated.
Arnhold et al. (2016)	Rotenone Conditions: ‐ ROT (10 nM, 100 nM, 1 µM, 5 µM) ‐ Vehicle Control (0.05% ethanol) Duration: 48, 96, 144 h	Sympathetic neurons (NMRI or C57BL/6J mice, 2–6‐day‐old)	Viability/Death: Bright‐field imaging, Adenylate kinase release assay Immunohistochemistry: β‐tubulin III, TH, α‐syn Markers: α‐syn by WB, qPCR and flow cytometry, TH by WB, qPCR GAPDH)	Dose‐dependent neurite degeneration Lewy body‐like α‐synuclein inclusions colocalizing with TH ↓ α‐synuclein and TH protein and mRNA levels → potentially linked to neurite degeneration Sympathetic neurons more resistant to rotenone than dopaminergic neurons (significant death only ≥ 1 µM C57BL/6, ≥ 5 µM NMRI)
Sharrad et al. (2017)	Rotenone Conditions: ‐ ROT (1, 10 µM) ‐ Untreated control Duration: 1, 2, 4 days	Ex vivo guinea pig ileum intestinal segments (adult, 230–400 g)	Immunohistochemistry: α‐syn (total, fibrils, oligomers); colabeling with ChAT, NOS, TH Imaging: quantification of neuronal cells	↑ α‐syn oligomers and fibrils (dose and time‐dependent) Concentration‐ and time‐dependent loss of cholinergic (ChAT‐IR) neurons (necrosis; ↑autofluorescence)
Guan et al. (2017)	Rotenone Conditions: ‐ 0.01–50 µM (RNA‐seq at 0.3 µM) ‐ Untreated control Duration: 48 h	Primary enteric neurons (small intestine of SD rats, 4‐day‐old)	Viability: CCK‐8 assay Transcriptomics: RNA‐seq; GO and KEGG pathway analysis; qPCR validation	Viability: ↓ to 69% at 0.21 µM, 25.8% at 0.52 µM 75 DEGs (45 ↑ / 30 ↓) linked to neurodevelopment, neurogenesis, neuronal differentiation and stimuli response Functional enrichment in MAPK, Wnt, TLR, Ras signaling pathways ↓NNAT, µgT8 (neuronal growth, nerve injury); ↑MGP, SERPINE2, ADAMTS1, NPY expression levels
Virga, Capps, and Vohra (2018)	Rotenone Conditions: ‐ 10 µM ROT ‐ Vehicle control Duration: 24, 48 h	Immunoselected p75NTR+ enteric neural crest cells (embryonic gut of CD1 mice, E12.5) Primary mixed enteric culture (embryonic gut of CD1 mice, E12.5)	Genetic manipulation: Lentiviral overexpression (cytNmnat1, Bcl‐xl, DsRed‐Mito, GFP reporters) Mitochondrial function: ATP levels (MgGreen‐AM assay), mitochondrial dynamics (time‐lapse imaging of fission/fusion, kymograph analysis of motility) Neuronal structure: Immunofluorescence staining (Tuj1, TH, Hoechst); neurite fragmentation counts, neuronal soma survival	Immunoselected neurons: more sensitive (24 h, neurites ↓ ∼80%, soma ∼59%; at 48 h, neurites ↓∼99%, soma 90%)Mixed culture: at 24 h, neurites ↓ ∼51%, soma ∼26%; at 48 h, neurites ↓ ∼83%, soma ∼55%Both models: ↓ Mitochondrial ATP levels, motility, and dynamics Neuritic and somal degeneration: cytNmnat1‐overexpression protected neurites only, while Bcl‐xL‐overexpression protected both neurites and somata (mitochondrial apoptotic pathways)
Miyazaki et al. (2019)	Rotenone Conditions: ‐ ROT (1, 2.5, 5 nM) for 48 h ‐ Caffeic acid (CA; 10 or 25 µM) or chlorogenic acid (CGA; 25 µM) for 24 h and cotreatment w/ rotenone (1, 2.5, 5 nM) for 48 h ‐ Untreated control	Primary enteric neuronal and glial cocultures (embryonic intestine of SD rats, E15)	Immunohistochemistry: β‐tubulin III, GFAP, MT‐1,2	ROT: dose‐dependent enteric neuronal loss (↓ β‐tubulin III); ↓ MT‐1,2 levels in enteric glia (GFAP^+^) CA and CGA pre‐/coexposure: ineffective at restoring neuronal signals at 1 or 2.5 nM ROT, showing neuroprotective effects only at 5 nM ROT
**Heavy metals**				
Ghaisas et al. (2021)	Manganese Conditions: ‐ MnCl_2_·4H_2_O (1, 3, 10, 30, 100, 300, 1000 µM) ‐ Untreated control Duration: 24 h	Rat enteric glial cell line (EGC ATCC, CRL‐2690)	Viability: MTS assay Mitochondria: morphology and mass (MitoTracker Red/Green), circularity, aconitase activity, OCR and ATP (Seahorse), mitochondrial superoxide (MitoSOX) Neurotransmission: [^3^H]‐glutamate uptake Cell death: SYTOX Green assay, caspase‐3 activity	Viability: IC_50_ = ∼5 µM Mitochondrial Dysfunction: ↓ Mitochondrial mass (10%–60% loss at 1–30 µM Mn), ↓ Aconitase activity, ↑ mitochondrial superoxide production, ↑circular morphology and ↓ATP‐linked respiration (100 µM Mn) Functional impairment: ↓ Glutamate uptake (∼10% at 5–10 µM and > 50% at 100 µM) Apoptosis: ↑ caspase‐3 activity, SYTOX staining at ≥ 300 µM Mn
Ghaisas et al. (2021)	Manganese Conditions: ‐ MnCl_2_·4H_2_O (1, 3, 10, 30, 100, 300, 1000 µM) ‐ Untreated control Duration: 24 h	Mouse primary enteric mixed culture (intestine of C57BL/6 mice, E15)	Viability: MTS assay Markers: Inflammation (TNFα, iNOS by qPCR); Neuronal/glial (PGP9.5, GFAP) and metal transporters (DMT1, Fpn) by WB Function: smooth muscle activity (contractions in long‐term cultures, video analysis)	Viability: IC_50_ = ∼98 µM Inflammation: ↑iNOS expression (36‐fold), ↑TNFα (threefold) Neuronal/glial markers: ↓PGP9.5, trend ↓GFAP Metal transporters: n.s. changes in DMT1 Fpn Spontaneous contractions in primary culture gradually reduced after 100 µM Mn treatment for 24h
**Acrylamide**				
Lourenssen, Miller, and Blennerhassett (2009)	Acrylamide Conditions: ‐ ACR (0.01–12 mM) ‐ Untreated control Duration: 24, 96, 144 h	Primary culture of rat myenteric neurons, glia, and smooth muscle (small intestine of SD rats, 4–11‐day‐old)	Viability/death: Calcein‐AM/PI, HuD immunostaining, cleaved caspase‐3, annexin V, caspase inhibition (zVAD, DEVD) Neuronal function: Immunocytochemistry (HuD, SNAP‐25, PGP9.5, βIII‐tubulin, nNOS, VAChT); [^3^H]‐choline uptake and K^+^‐stimulated [^3^H]‐ACh release Neuronal structure: Axon counts	Axonal Damage: ∼30% axon loss at 0.5–2.0 mM without neuron loss; severe loss at 4 mM Cell Death: No loss at 1 mM (24 h); significant loss (∼40%) at 4 mM primarily via necrosis Functional Impairment: ↓ ACh release (≥ 0.5 mM), ↓ SNAP‐25 and syntaxin, while nNOS/VAChT proportions unchanged Axon number recovered/regenerated by 96h postexposure (1–2 mM), however ACh release remained suppressed
**Toxins**				
Reale et al. (2021)	Pectenotoxin Okadaic acid Conditions: ‐ PTX2 (50, 100, 180, 260 nM) ‐ OA (100, 150, 200, 320 nM) ‐ Untreated vehicle control (5% MeOH) Duration: 24 h	Tri‐culture transwell model: Caco‐2/HT29‐MTX (3:1, 23‐day‐differentiated) in AP compartment Rat enteric glial cell line (EGC ATCC, CRL‐2690) in BL compartment	Viability: Neutral Red uptake assay (Caco‐2/HT29‐MTX, EGC) Barrier integrity: TEER, LY permeability, ZO‐1 immunofluorescence Inflammation: ELISA (IL‐8 release) Gene expression: RT‐qPCR (inflammatory, tight junction, transporters, mucins, gliomediators) Toxin transport: LC–MS/MS quantification	In PTX2 groups: Caco‐2/HT29‐MTX: ↓ Viability (∼20% at 50 nM); ↓ Barrier integrity (↓64%–83% TEER at 180, 260 nM; ↑ LY permeability; ↓ ZO‐1); ↑IL‐8 release in AP and mRNA expression (dose‐dependent), ↑mRNA CLDN4, MUC13; no PTX2 detected in BL after 24 transport EGC: No viability effect; neurite retraction and cell body shrinkage (> 180 nM); mild glial response (↑ IL‐6; no change in GDNF/iNOS) In OA groups: Caco‐2/HT29‐MTX: ↓ Viability (∼20% at 100 nM); ↓ Barrier integrity (↓38%–87% TEER at 200, 320 nM; ↑ LY permeability; ↓ ZO‐1); ↑↑ IL‐8 release (dose‐dependent, up to 14‐fold in AP levels and sixfold in mRNA); ↑CLDN4, MUC13, ABCC5, ABCB1 expression; partial OA transport (8%–29% OA in BL) EGC: ↓ Viability (∼28% at 320 nM); No morphological alterations; glial response ↑ GDNF protein (1.5‐fold, 150 nM) and mRNA (3.5‐fold, 175 nM); ↑ BDNF (3.8‐fold mRNA)
Brand et al. (2019)	Patulin (purified from *P. coprobium* extract) Conditions: ‐ PAT (0.5, 10 µM) ‐ Untreated vehicle control (1% DMSO) Duration: 24 h	Mouse primary enteric neurons (intestines of C57B6/J OlaHsd mice, 2–3‐month‐old)	Viability: CellTiter‐Glo Oxidative stress/mitochondria: ROS, calcium influx, glucose metabolism (fluorescence‐based assays) Neuronal structure: Neurite outgrowth (crystal violet solution)	Viability: ↓ ∼46% at 10 µM Structural alterations: ↓ Neurite mass (∼70%) at 0.5 µM (subtoxic) Cellular dysfunction: ↓ Glucose content; ↑ Membrane depolarization (Ca^2^^+^ influx)
Dabrowski et al. (2025)	Aflatoxin B1 Apicidin Aurofusarin Beauvericin Brevianamide‐F Cyclo‐(L‐Pro‐L‐Tyr) Deoxynivalenol Emodin Enniatins Fumonisin B1 Moniliformin Ochratoxin A Patulin Tryptophol Zearalenone Conditions: ‐ Mycotoxins (individually tested up to 100 µM) ‐ Untreated vehicle control (10% ethanol or 1% DMSO^a^) Duration: 48 h	Rat enteric glial cell line (EGC ATCC, CRL‐2690)	Viability: Resazurin assay Oxidative stress: ROS (DCFDA assay) Membrane integrity: Propidium iodide Apoptosis: Caspase‐3/7 (fluorescence assay)	Antiproliferative Effect (IC50): AFB1 = FB1 = ZEN = BRV‐F = CYCLO = MON = TRPT (no effect) < AFN (79.51 µM) < EMO < OTA < PAT < API < BEA < ENN B < ENN A1 < ENN A < ENN B1 < DON (0.19 µM) Viability (CC50): AFN = AFB1 = FB1 = BRV‐F = CYCLO = EMO = MON = TRPT (no effect) < API (59.59 µM) < PAT < ZEN < OTA < DON < ENN B1 < BEA < ENN A < ENN A1 < ENN B (0.72 µM) Oxidative stress: ↑ ROS production at 48 h for DON, PAT, ZEN and starting at 12 h for OTA at CC50 levels Loss Membrane Integrity/Apoptosis: Time‐dependent effects for DON, OTA, PAT, ZEN, AP, ENNs and BEA at CC50 levels
**Nanoplastics**				
Liang et al. (2025)	PS‐NPs (50 nm, spherical, nonfluorescent, FITC or PE‐labeled PS) Conditions: ‐ PS‐NPs (200 µg/mL) ‐ A53T α‐syn (5, 10, 15 µM) ‐ PS‐NPs + A53T α‐syn ‐ Untreated control Duration: 24 h	Mouse enteric glial cell line	Viability: CCK‐8 assay Oxidative stress/mitochondria: ROS, mitochondrial Ca levels, NO analysis, MMP test, lysosomal permeability (flow cytometry analysis) Senescence: β‐Galactosidase staining Markers: qPCR (GFAP, S100β), ELISA (IL‐1β, TNFα, IL‐6, COX‐2), metabolomic (untargeted screening)	In PS‐NPs group: ↓ Viability (81 ± 11%) ↑ Oxidative stress (↑ROS), mitochondrial dysfunction (↓ MMP and ↑calcium influx) and lysosomal damage ↑ Glial activation and inflammation (↑ NO production, ↑ IL‐1β, TNF‐α, IL‐6, COX‐2 levels) Shift in metabolite profile linked to neurodegeneration Coexposure (PS‐NPs + A53T α‐syn) exacerbated all effects

Abbreviations: ACh, acetylcholine; ABCB1 and ABCC5, ATP‐binding cassette transporters; AP, apical compartment; A53T α‐syn, alpha‐synuclein with A53T mutation associated with PD; Bcl‐xl, B‐cell lymphoma‐extra‐large; BDNF, brain‐derived neurotrophic factor; BL, basolateral; CCK8, Cell Counting Kit‐8; ChAT^+^, choline acetyltransferase–positive neurons; CMMC, colonic migrating motor complex; CLDN4, claudin‐4; cytNmnat1, cytoplasmic nicotinamide mononucleotide adenylyltransferase 1; DF, dietary factors; DEG, differentially expressed gene; DMT1, divalent metal transporter 1; EFS, electrical field stimulation; FITC, fluorescein isothiocyanate; Fpn, ferroportin; GDNF, glial cell line–derived neurotrophic factor; GFAP, glial fibrillary acidic protein; IL‐6 and IL‐8, interleukin‐6 and interleukin‐8; IR, immunoreactivity; iNOS, inducible nitric oxide synthase; LY, lucifer yellow; MAPK, mitogen‐activated protein kinase; MGP, matrix Gla protein; MMP, mitochondrial membrane potential; MT‐1,2, metallothionein‐1 and ‐2; MTS, 3‐(4,5‐dimethylthiazol‐2‐yl)‐5‐(3‐carboxymethoxyphenyl)‐2‐(4‐sulfophenyl)‐2H‐tetrazolium; MUC13, mucin 13; nNOS, neuronal nitric oxide synthase; NR assay, neutral red assay; NPY, neuropeptide Y; n.s., not significant; PARP, poly(ADP‐ribose) polymerase; PE, phycoerythrin; PGP9.5, protein gene product 9.5; PS‐NPs, polystyrene nanoparticles; ROS, reactive oxygen species; SD, Sprague Dawley; SERPINE2, serpin family E member 2; SNAP‐25, synaptosomal‐associated protein 25; SYTOX, SYTOX Green; TEER, transepithelial electrical resistance; TLR, Toll‐like receptor; TNF‐α, tumor necrosis factor alpha; µgT8, UDP glycosyltransferase 8; VAChT, vesicular acetylcholine transporter; ZO‐1, zonula occludens‐1; arrows (↑/↓), indicate increase or decrease, respectively.

^a^See **Table** **S4** for specific solvents used for each mycotoxin.

**TABLE 3:** Summary of treatment regimen and key findings of animal studies investigating the effects of FCCs on the enteric nervous system.

Study	Treatment regimen	Enteric neuronal‐related effects	GI effects
**Pesticides**			
Darwiche et al. (2017)	**Chlorpyrifos** Model: Wistar rats’ offspring (male; 60‐day‐old) Exposure: Oral gavage via maternal exposure until early adulthood (GD1 → PND60) CPF1 – 1 mg/kg bw/day CPF5 – 5 mg/kg bw/day Control (rapeseed oil)	Ileum: ↓AChE activity in gut (about −30% in CPF1, −59% in CPF5)	Excessive NO production in ileum (CPF5): ↓Ileum contraction, reversed by NOS inhibitor L‐NAME ↑iNOS mRNA levels (3.7‐ and 3.6‐fold in CPF1, CPF5) and protein (1.55x CPF1 n.s.; 1.75x CPF5) ↓Circular/longitudinal muscle layers in ileum (CPF1, CPF5) Normal intestinal transit time Other findings: CPF5: ↓bw at birth; CPF1/5: ↓bw at PND60
Pupim et al. (2023)	**Malathion** Model: Wistar rats (male; 25‐day‐old) Exposure: Oral gavage for 40 days M10 – 10 mg/kg bw/day M50 – 50 mg/kg bw/day Control (0.9% saline solution)	↓ BuChE activity in colon (−35% in M10: −46% in M50) Proximal colon SP: n.s. total neurons, ChAT, nNOS‐IR neurons; neuronal cell body hypertrophy/atrophy in M10/M50, ↓ChAT ratio in M50; respectively; MP: n.s alterations in total neurons, ChAT, nNOS‐IR neurons; M10 induce atrophy Distal colon SP: n.s. total neurons; neuronal cell body hypertrophy in M10/M50; MP: ↓total neurons, ↓nNOS neurons in M10; M10‐induced atrophy; M50‐induced hypertrophy in ChAT neurons	M10: Mucosa atrophy M50: ↑ Fecal mass and water content; mucosa hypertrophy and ↑crypt depth In both groups: ↑ Stool number and size ↓ Goblet cells Dose‐dependent colonic dysmotility: impaired coordination, altered contractile force and disrupted transit patterns
Nanni et al. (2022)	**2,4‐Dichlorophenoxyacetic acid** Model: Wistar rats (male, 60‐day‐old) Exposure: Oral gavage for 15 days 5 mg/kg bw/day Control (distilled water)	Colon: ↑ Density myenteric neurons (80.1 ± 31.9 neurons/mm^2^ vs. 73.4 ± 11.3 neurons/mm^2^ control) No alterations in nitrergic (NADPH‐d^+^) neuron density or morphology	No alterations in colon area
Bulc, Calka, and Palus (2023), Palus et al. (2024), Palus, Karpiesiuk, and Jana (2025)	**Glyphosate** Model: Danish Landrace pigs (female; 8 weeks) Exposure: Oral (gelatine capsules) for 28 days LD – 0.05 mg/kg bw/day HD – 0.5 mg/kg bw/day Control (empty gelatine capsules)	Colon: ↑nNOS‐IR, VIP‐IR, GAL‐IR, PACAP‐IR neurons in HD group (Figure S1) Small intestine: dose‐dependent ↑GAL‐IR neurons across all plexus (highest in ileum) Jejunum: dose‐dependent ↑CART‐IR, CGRP‐IR, nNOS‐IR, but ↓VChAT‐IR neurons across all plexus GALR1‐3 receptors expression: ↓duodenum in LD (0.2 and 0.3‐fold decreased for GALR2, GALR3) and HD (0.1‐fold for GALR1); n.s. in jejunum; ↑ileum (up to 5.2‐fold and 3.3 for LD, HD, respectively)	SOD2 mRNA levels: ↑ duodenum in HD; ↓ jejunum, ileum in LD (0.8, 0.4‐fold, respectively); n.s. alterations in SOD1
Arnhold et al. (2016)	**Rotenone** Model: C57BL/6J mice (8‐week‐old) Exposure: Oral gavage for 4 months 5 mg/kg bw/day, 5 days/week Control (2% carboxymethylcellulose and 1.25% chloroform)	Colon: ↓ Dopaminergic sympathetic neurites ↑ α‐syn inclusions	Intestinal function: ↓ Stool output (hypomotility)
Schaffernicht et al. (2021)	**Rotenone** Model: C57BL/6J mice (1‐year‐old) Exposure: Oral gavage for 2 and 4 months 5 mg/kg bw/day, 5 days/week Control (2% carboxymethylcellulose and 1.25% chloroform)	Duodenum, jejunum, colon: ↑TH (duodenum, colon); ↓TH (jejunum) only after 2 months exposure ↓Cholinergic (ChAT‐IR) neurons at 2 and 4 months exposure	Intestinal function: Alterations in intestinal physiological response to carbachol, dopamine and EFS related to ↓cholinergic /noradrenergic input
McQuade et al. (2021)	**Rotenone** Models: WT mice (female, 12‐week‐old) A53T mice (female, 12‐week‐old; transgenic human A53T α‐syn model) Exposure: Oral gavage for 28 days 30 mg/kg bw/day Control (1.25% (v/v) chloroform and 1% (w/v) carboxymethyl cellulose)	Colon: WT ROT – ↓ Hu+ neuron density (↓ ∼30%), ↑ Hu+ nuclear translocation (∼24%), no change in nNOS proportion or soma size A53T ROT – ↑ Hu+ nuclear translocation (∼24%), no change in nNOS or soma size	Intestinal function: WT ROT – n.s. changes in fecal pellet output, bead expulsion time, or fecal water content A53T ROT – ↓ FPO (∼50% reduction), ↓ bead expulsion time (faster expulsion, ∼58 s vs. ∼108 s in controls), no change in fecal water content
**Heavy metals**			
Ghaisas et al. (2021)	**Manganese** (MnCl_2_·4H_2_O) Model: C57BL/6 mice (male, 8–10‐week‐old) Exposure: Oral gavage for 30 days 15 mg/kg bw/day Control (water)	Colon: ↑ GFAP expression, ↑iNOS ↓ Mitochondrial protein DJ‐1 levels No alterations in enteric neurons (PGP9.5^+^) → selective enteric toxicity	Inflammation: ↑ TNFα (2.8‐fold) and ↑ iNOS (2.6‐fold) expression Loss Mn homeostasis: ↓trend Fpn protein levels, n.s alterations in Slc30a10 (mRNA), DMT1 protein levels, ↑Mn content in colon tissue Intestinal function/structure: ↓PAS staining (mucus), ↓goblet cells in colon, longer intestinal transit times (n.s.) Other findings: No changes in gut microbiome composition (↑ trend Gammaproteobacteria at 20 days Mn); Altered fecal metabolites (fatty acids, a.a, and sugars)
**Acrylamide**			
Palus, Bulc, and Calka (2018a), Palus, Makowska, and Calka (2018b), Palus and Calka (2019), Palus, Makowska, and Calka 2019a; Palus et al. (2019b), Palus, Bulc, and Calka (2020a, 2020b), Bulc, Calka, and Palus (2022), Karpiesiuk, Calka, and Palus (2023)	**Acrylamide (ACR)** Model: Danish Landrace pigs (female; 8‐week‐old) Exposure: Oral (gelatine capsules) for 28 days LD – 0.5 µg/kg bw/day HD – 5 µg/kg bw/day Control (empty gelatine capsules)	Stomach (Figure S2): ↑nNOS‐IR, CGRP‐IR, SbP‐IR, GAL‐IR, CART‐IR, VIP‐IR, VAChT‐IR neurons in gastric MP (cardia, corpus, pylorus) and SP of corpus region ↑GAL‐IR in both groups and only detected in cardia submucosa ↑GAL/VIP, GAL/nNOS and GAL/CART colocalization in all gastric plexus (∼1.1–1.4‐fold increase for HD group) Small intestine: ↑PACAP‐IR, VAChT‐IR, CGRP‐IR, VIP‐IR, CART‐IR, GAL‐IR, SbP‐IR neurons in both groups ↑nNOS‐IR in duodenum, but ↓nNOS‐IR in jejunum and ileum plexus	Inflammation: Stomach – Leukocyte infiltration and mucosal inflammation Ileum – ↑ proinflammatory cytokines (IL‐1β, IL‐6, TNF‐α; significant in HD group)
**Toxins**			
Rissato et al. (2020)	**Deoxynivalenol (DON)** Model: Wistar rats (male, 21‐day‐old) Exposure: Oral (experimental diet containing 0.2, 0.75, 1.75, or 2 mg DON/kg chow) for 42 days 0.0145, 0.0584, 0.141, 0.157 mg/kg bw/day Control	Jejunum: No alteration in total neuronal density and nNOS, ChAT, NADH‐d^+^ subpopulations in MP ↓gliocyte area, neuronal body area	Oxidative stress: No alterations in SOD, GST activity, levels of nonprotein sulfhydryl groups (GSH), and lipidic hydroperoxides Other findings: No impact on food consumption, bw or intestinal area
Makowska et al. (2017), Makowska, Obremski, and Gonkowski (2018), Rychlik et al. (2020)	**T‐2 toxin** Model: White Polish pigs (female; 8‐week‐old) Exposure: Oral (gelatine capsules) for 42 days 12 µg/kg/day Control (empty gelatine capsules)	Stomach/Duodenum: ↑VIP‐IR, ↑CART‐IR, ↑nNOS‐IR neurons Descending Colon: ↑CART‐IR, ↑CGRP‐IR neurons; n.s. alterations in CGRP/VAchT‐IR colocalization (Figure S3)	—
Kras et al. (2022)	***Fusarium verticillioides* extract (100 mg/mL; 73% FB1/27% FB2)** Model: Wistar rats’ offspring (maternal exposure) Exposure: Intragastric route for 21 days (PGD7 to PND28) F60—60 mg FBs/kg bw/day F90—90 mg FBs/kg bw/day Control (0.9% saline solution)	Duodenum: ↑VIP‐IR neurons (1.3x, 1.5x for FB60, FB90) and GAL‐IR neurons (1.3x, 1.4x for FB60, FB90) in MP No morphologic alterations	Intestinal structure: FB90 – ↑ villus width (but not number or height) and crypts density (but not depth and width) FB60/90 – no differences the duodenum sublayers thickness
Rudyk et al. (2020)	***Fusarium verticillioides* extract (75% FB1 182 µg/kg, 25% FB2 59.5 µg/kg)** Model: Wistar rats (male, 5‐week‐old) Exposure: intragastric administration for 21 days 90 mg/kg/day Control (0.9% saline solution)	Duodenum, Jejunum: ↓size of jejunal plexus and duodenal MP	Intestinal structure: Villus atrophy, crypt hyperplasia Other findings: Hepatotoxicity (↑ serum ALT, AST)
Sousa et al. (2014)^a^	***Fusarium verticillioides* extract (∼75% FB1, 25% FB2 ratio)** Model: Wistar rats (male, 21‐day‐old) Exposure: Oral (experimental diet) for 15 or 42 days F1 – 103.30 ± 12.25 µg FBs/kg bw/day F3 – 311.20 ± 40.89 µg FBs/kg bw/day Control – 17.11 ± 2.13 µg FBs/kg bw/day.	Jejunum: No alterations in morphological organization of MP n.s differences in total or nitrergic neuronal populations but reduction of area	Other findings: No impact on food consumption or body weight
Sousa et al. (2022)^b^	***Fusarium verticillioides* extract (∼80% FB1, 20% FB2 ratio)** Model: Wistar rats (male, 21‐day‐old) Exposure: Oral (experimental diet) for 42 days F1 – 130.80 ± 44.31 µg FBs/kg bw/day F4 – 439.00 ± 134.60 µg FBs/kg bw/day Control(experimental diet without fungi extract)	Jejunum: No alterations in nitrergic (NADPH‐d^+^ neurons) but reduction in size ↓metabolic activity of myenteric neurons	Other findings: No impact on food consumption or body weight
Makowska et al. (2017)	**Zearalenone (ZEN)** Model: White Polish pigs (female; 8‐week‐old) Exposure: Oral (gelatine capsules) for 42 days 6 µg/kg/day Control (empty gelatine capsules)	Descending colon: ↑CGRP‐IR neurons (up to 1.7‐fold in MP) ↑CGRP‐IR colocalization with nNOS, SbP, GAL, CART; n.s. alterations in CGRP/VAchT colocalization	—
**MNPs**			
Galecka and Calka (2024a, 2024b); Galecka, Szyrynska, and Calka (2024)	**PET‐MPs** (various sizes; average 160 µm) Model: Petrain × Duroc pigs (female; 8‐week‐old) Exposure: Oral (gelatine capsules) for 28 days LD – 0.01 g/animal/day HD – 0.5 g/animal/day Control (empty gelatine capsules)	Small intestine (Figure S4): ↓nNOS‐IR and VAChT‐IR and VIP‐IR neurons ↑CART‐IR only in duodenum; ↑GAL‐IR in duodenum and jejunum; ↑SbP‐IR in jejunum and ileum LD group induced relevant downregulation of intestinal VIP‐IR neurons	Duodenum: villus shortening (LD, HD), ↓ mucosa, ↑ muscularis (HD), epithelial loss, mucus build‐up Jejunum: villus atrophy, epithelial damage, eosinophil infiltration, hyperemia (more severe in HD)
Augustyniak et al. (2025)	**PS‐NPs‐NH_2_** (25 nm) Model: Rat (14‐day‐old) Exposure: Intragastric administration for 21 days 1 mg/kg bw/day Control (0.9% saline solution)	Jejunum: ↓ s100b and ↓ cspg4 mRNA expression, indicating enteric glial and glia‐like cell impairment No significant changes in map2 expression, suggesting preserved enteric neuronal marker levels Evidence for selective ENS support cell vulnerability rather than neuronal loss	Nanoplastic accumulation in epithelial and subepithelial layers Epithelial alterations: ↓ fabp2 (enterocytes), ↓ chga (enteroendocrine cells), ↑ lgr5 (stem cells), ↑ lyz1 (Paneth cells) Mucus/barrier response: ↑ muc1, ↑ cldn1, ↑ ocln (adaptive/compensatory), no change in muc2, cldn2, cdh1 Inflammation: ↑ Il1b and ↑ tnf (mRNA and protein) Oxidative stress: ↑ protein carbonylation, mitochondrial ultrastructural damage, ↓ CAT and SOD1 activity Metabolomic shifts consistent with barrier dysfunction, altered energy metabolism, and inflammatory remodeling
**Bisphenols**			
Makowska, Lepiarczyk, and Gonkowski (2022), Makowska, Calka, and Gonkowski (2023), Makowska, Fagundes, and Gonkowski (2023), Makowska and Gonkowski (2023, 2024)	**Bisphenol A (BPA)** **Bisphenol S (BPS)** Model: CD1 mice (male and female; 3‐month‐old) Exposure: Oral (drinking water) for 3 months LD BPA or LD BPS – 5 mg/kg bw/day HD BPA or HD BPS – 50 mg/kg bw/day Control (water)	Stomach, jejunum: ↓VAChT‐IR neurons under both BPA and BPS exposure Colon: region with more pronounced neurochemical changes (↑ VAChT‐IR, nNOS‐IR, CART‐IR, VIP‐IR, SbP‐IR, GAL‐IR neurons) under both BPA and BPS exposure Alterations were dose‐dependent, but significant effects observed even at low dose, particularly for LD BPS in the colon (Figure S5)	Intestinal structure: n.s. colonic histopathological alterations
Makowska et al. (2025)	**Bisphenol A (BPA)** **Bisphenol S (BPS)** Model: CD1 mice (male and female; adult) Exposure: Oral (drinking water) for 15 days 50 mg/kg bw/day of BPA or BPS Control (water)	Colon: ↓VAChT‐IR myenteric neurons and Ano‐1 positive interstitial cells of Cajal (neuromuscular but not ganglionic level) under both BPA and BPS exposure No alterations in enteric glia (S100β‐positive)	
Szymanska, Calka, and Gonkowski (2018), Szymanska and Gonkowski (2018), Szymanska, Makowska, and Gonkowski (2018), Szymanska and Gonkowski (2019), Gonkowski et al. (2020), Makowska and Gonkowski (2020), Szymanska et al. (2020), Makowska et al. (2021), Makowska and Gonkowski (2022)	**Bisphenol A** Model: Petrain x Duroc pigs (female; 8‐week‐old) Exposure: Oral (gelatine capsules) for 28 days LD – 0.05 mg/kg bw/day HD – 0.5 mg/kg bw/day Control (empty gelatine capsules)	↓VAChT‐IR neurons across stomach, small intestinal and colon plexus Small Intestine: Duodenum – ↓VIP‐IR, VAChT‐IR, CART‐IR, GAL‐IR neurons Jejunum – ↓ nNOS‐IR and VAChT‐IR neurons Ileum – ↓ VAChT‐IR, VIP‐IR neurons; ↑ nNOS‐IR, CART‐IR, SbP‐IR, GAL‐IR neurons Colon: ↓ VAChT‐IR neurons; ↑ NRG1‐IR and colocalization with nNOS, VIP, SbP, GAL; Caecum: ↓total neurons; ↑ calbindin‐D28k‐IR neurons (Figure S6)	Inflammation: Ileum (HD) – ↑IL‐1β, TNF‐α, Peyer's patch hypertrophy, mucosal infiltration

Given the relevance of gut–brain communication, reported effects on the central nervous system (CNS), intestinal function, and others such as microbiota are also included.

Abbreviations: α‐syn, α‐synuclein; AChE, acetylcholinesterase; AgRP, Agouti‐related peptide; ARC, arcuate nucleus; BuChE, butyrylcholinesterase; bw, body weight; CART, cocaine‐ and amphetamine‐regulated transcript; CAT, catalase; CGRP, calcitonin gene‐related peptide; CMMC, colonic migrating motor complex; DMT1, divalent metal transporter 1; DMV, dorsal motor nucleus of the vagus; DOPAC, 3,4‐dihydroxyphenylacetic acid; FBs, fumonisins; Fpn, ferroportin; GAL, galanin; GALR, galanin receptor; GD, gestational day; GFAP, glial fibrillary acidic protein; GST, glutathione S‐transferase; HD, high group; IL, interleukin; iNOS, inducible nitric oxide synthase; IR, immunoreactivity; ISP, inner submucosal plexus; LD, low dose; L‐DOPA, levodopa; L‐NAME, N‐nitro‐L‐arginine methyl ester; MP, myenteric plexus; mPGES‐1, microsomal prostaglandin E synthase‐1; NADH‐d+, nicotinamide adenine dinucleotide diaphorase‐positive; NO, nitric oxide; nNOS, neuronal nitric oxide synthase; NPY, neuropeptide Y; Nrf2, nuclear factor erythroid 2‐related factor 2; NRG1, neuregulin 1; n.s., not significant; NTS, nucleus tractus solitarius; OSP, outer submucosal plexus; PACAP, pituitary adenylate cyclase‐activating polypeptide; PAS, periodic acid–Schiff; PGP9.5, protein gene product 9.5; PND, postnatal day; POMC, pro‐opiomelanocortin; SbP, substance P; Slc30a10, solute carrier family 30 member 10; SNpc, substantia nigra pars compacta; SOD, superoxide dismutase; SP, submucosal plexus; TH, tyrosine hydroxylase; *T*_max_, time to maximum concentration; UCP‐2, uncoupling protein 2; UCV, unilateral cervical vagotomy; VChAT, vesicular choline acetyltransferase; VIP, vasoactive intestinal peptide.

^a^ Sousa et al. (2014) experimental diet without fungi extract (F0) had 0.159 mg FB1/kg diet and FB2 n.d.; fungi extract was added to F1 diet (0.996 mg FBs/kg) and F3 diet (2.819 mg/kg) at ∼75% FB1/25% FB2.

^b^ Sousa et al. (2022) fungi extract was added to F1 diet (1.129 mg FBs/kg) and F3 diet (3.850 mg FBs/kg) at ∼80% FB1, 20% FB2.

**TABLE 4:** Summary of in vivo studies assessing the effects of food contaminants (FCs) on the enteric nervous system (ENS) and central nervous system (CNS).

Study	Treatment regimen	ENS effects	CNS effects	Other and GI effects
**Pesticides**				
Anadon et al. (2006)	**λ‐cyhalothrin (λ‐CYH)** Model: Wistar rats (adult male) Exposure: Single oral gavage 20 mg/kg bw	λ‐CYH concentrations were higher in nervous tissues compared to plasma (25.12 ± 1.83 µg/g in MP vs. 15.65 ± 1.78 µg/g in plasma) Prolonged elimination half‐lives in neural tissues suggests preferential retention and accumulation in the nervous system (T₁/_2_β: 22.71 ± 1.79 h in MP)	λ‐CYH found in brain (11.8–24.1 µg/g), highest in hypothalamus T₁/_2_β: 34.82 ± 1.97 h in hypothalamus	Slow absorption (*T*_max_ 2.69 h) High oral bioavailability (67%)
Naudet et al. (2017)	**Paraquat (PQ)** Models: TgHuA53T mice (male/female, 8‐week‐old, overexpression of mutant human α‐syn) C57Bl/6 mice (female, 8‐week‐old; 18–22 g) Exposure: Oral (drinking water) for 6–8 weeks 10 mg/kg bw/day Untreated control	TgHuA53T mice: Earlier and sustained ↑ in pSer129 α‐syn levels (vs. TgHuA53T control mice) ↑ Enteroglia activation (GFAP^+^) detected from 6 weeks of PQ exposure C57Bl/6 mice: Phosphorylated α‐syn not detected in the ENS of PQ‐treated mice ↑ Enteroglia activation (GFAP^+^) → glial activation independent of α‐syn aggregation	TgHuA53T mice: No locomotor deficits No effect on phosphorylated α‐syn expression in the brain, including the DMV C57Bl/6 mice: no locomotor deficits	TgHuA53T mice: n.s. alterations in bw or survival
Anselmi et al. (2018)	**Paraquat (PQ)** Model: Sprague‐Dawley rats (male; unknown age) Nonoperated animals Truncal vagotomy before P + L treatment (V/P + L) Exposure: Oral gavage for 7 days P—1 mg/kg bw/day L—0.05% lectin (*P. sativum*) P + L group Control (1% sucrose)/Sham control Recovery for 2 or 4 weeks before analysis	P + L group: Accumulation of pSer129 α‐syn in myenteric neurons Truncal Vagotomy (V/P + L): Accumulation of pSer129 α‐syn in myenteric neurons Vagotomy blocked α‐syn spread to the brain	P + L group: pSer129 α‐syn present in ChAT^+^ and TH^+^ neurons of the DMV and SNpc Impaired motor performance (recovery with L‐DOPA) P, L and V/P + L groups: No pSer129 α‐syn accumulation in brain No motor impairments	Gastric motility: impaired in P + L group but not in P, L, V/P + L groups
Tasselli et al. (2013)	**Rotenone (ROT)** Model: C57BL6N mice (male, 8–10‐weeks‐old) Exposure: Oral gavage for 28 days 30 mg/kg bw/day Control (4% CHCl_3_, 0.5% CMC)	No change in total, ChAT^+^, or nNOS^+^ neurons in the jejunum, ileum, or proximal colon ↓ α‐syn expression in jejunum, not ileum or colon	∼30% loss of TH^+^ neurons in SNpc ↓ Dendritic density Motor impairment	Intestinal function: Normal epithelial permeability ↓ Fecal output; Normal gastric emptying, transit time, and bead latency Other findings: Stable bw and food intake; high mortality within first 10 days
Pan‐Montojo et al. (2010)	**Rotenone (ROT)** Pilot study: C57BL/6J mice (8‐weeks‐old); Exposure: 2.5, 5, 10, 20 mg/kg bw/day via oral gavage; Duration: 7 days Main study: C57BL/6J mice (1‐year‐old) Exposure: Oral gavage 5 days/week for 1.5 or 3 months 5 mg/kg/day Control (4% CMC, 1.25% CHCl_3_)	At 1.5 months: ↑ α‐syn in duodenum/ileum ENS with diffuse somatic and extra‐somatic distribution ↑ Total α‐syn surface and inclusions number At 3 months: Larger α‐syn inclusions (> 6 µm) in 20% of ganglia Less α‐syn inclusions but ↑ size and α‐syn aggregates Persistent detection of pSer129 α‐syn Enteric gliosis	Pilot study: ROT (≤ 10 mg/kg) not detected in brain At 1.5 months: DMV – α‐syn in ChAT^+^ neurons, lipofuscin, GFAP^+^ gliosis, MHC II^+^ microglia IML – α‐syn in 2% ChAT^+^ neurons; no neuron loss At 3 months: SNpc – ↑ intracellular α‐syn, large inclusions; ↓ dopaminergic (TH^+^) neuron loss, partial TH downregulation DMV and IML – sustained α‐syn pathology without cell loss; no α‐syn detected in cortex, striatum, or cerebellum	Pilot study: ROT (≤ 5 mg/kg) not detected in blood → ruled out system toxicity
Pan‐Montojo et al. (2012)	**Rotenone (ROT)** Model: C57Bl/6J mice (1‐year‐old) Nonoperated animals (NORT) Hemivagotomized (HRT) Sympathectomized (SRT) Exposure: Oral gavage for 2 or 4 months 50 mg/kg bw/day; 6 days/week Control/Sham controls	NORT: ↑ α‐syn in ChAT+ neurons in the IML HRT/SRT: prevented α‐syn accumulation in disconnected regions; nerve resection blocked transneuronal spread of α‐syn and delayed PD‐like progression	NORT: progressive α‐syn accumulation in DMV and SN, dopaminergic cell death in SNpc, ↓ motor performance (rotarod); HRT/SRT: delayed α‐syn increase in still‐connected regions; HRT prevented dopaminergic cell death in ipsilateral SNpc	
Ahn et al. (2020)	**Rotenone (ROT)** Models: Human SNCA‐null transgenic mice (3‐month‐old) Human SNCA‐overexpressing mice Nonoperated animals Hemivagotomized (HMV) Exposure: Oral gavage overexpressing mice 50 mg/kg bw ROT, 6 days/week for 2 or 4 months Colonic injection of α‐Syn N103/Tau N368 preformed fibrils (PFFs) PFFs colonic injection + ROT exposure	Rotenone triggered AEP activation in colon, cleavage of α‐Syn (N103) and Tau (N368), pS129 α‐Syn accumulation, and TH loss in enteric neurons Colon‐inoculated PFFs spread along the vagus nerve to DMV; hemivagotomy blocked pathology Constipation, reduced stool moisture, prolonged GI transit, shortened colon length	Dopaminergic neuron loss in SN (TH+ neurons), α‐Syn pS129 inclusions, Tau AT8 pathology Pathology propagated from gut → DMV → SN/striatum → LC/HC via vagus nerve AEP KO abolished α‐Syn/Tau cleavage, dopaminergic loss, and motor deficits Intrastriatal PFFs induced widespread α‐Syn pS129 pathology in SN, cortex, amygdala, OB Behavioral impairments: motor deficits (rotarod, grid), cognitive decline (Morris water maze, novel object recognition), depression‐like behavior (tail suspension)	GI dysfunction: constipation, delayed transit, colon shortening Stronger pathology with α‐Syn N103/Tau N368 PFFs vs. FL or single fibrils
**Toxins**				
Girardet et al. (2011)	**Deoxynivalenol (DON)** Models: DBA/1lac J mice (male, adult): Nonoperated animals Unilateral cervical vagotomized (UCV) POMC‐Tau‐Topaz GFP transgenic mice Exposure: Single oral exposure 12.5 mg/kg DON Control/Sham control Note: DON group also received 6.25, 12.5, 25 mg/kg for 24 h food intake measurement	UCV group: vagotomy did not alter DON‐induced cFos expression in brainstem → DON anorexigenic effect does not rely on vagal afferent signaling	DBA/1lac J mice Appetite‐related markers – ↑cFos expression in brainstem and diencephalon (after 3 h) POMC mice: ↑ cFos neuronal expression in NTS, ARC ↑ cFos/nesfatin 1 ↑cFos/TH+ neurons in brainstem (15%) ↑POMC, CART, MC4R mRNA in hypothalamus (n.s alterations in NPY, AgRP) → selective activation of anorexigenic pathways	DBA/1lac J mice: Anorexic response (dose‐dependent ↓ night‐time food intake)
Gaige et al. (2014)	**T‐2 toxin** Model: C57Bl6 mice (adult male) Nonoperated animals Animals subjected to unilateral cervical vagotomy (UCV) Exposure: Single oral gavage 0.5, 2, 5 mg/kg bw (food intake studies) 2 mg/kg bw Control (0.2%–2% DMSO) Evaluation throughout 24‐h‐post‐treatment	UCV group: Vagotomy partially attenuated c‐Fos activation in the brainstem	Appetite‐related markers: ↓ brainstem NPY at 3 h ↑ hypothalamic AgRP (agouti‐related peptide; orexigenic) at 24 h → related with food intake rebound ↑ cFos expression in brain stem, hypothalamic nuclei and central amygdala (after 3 h 2 mg/kg T2); ↑ cFos/nesfatin‐1 colocalization Neuroinflammation: ↑ COX‐2, CAT hypothalamus and DVC ↑ 1.6‐fold TNFα in hypothalamus No alterations in IL‐1B, IL‐6 mPGES‐1, Nrf2, UCP‐2, and SOD‐2 mRNA Microglia: no alterations in GFAP, vimentin, Iba‐1 expression ↓ Locomotor activity at 12 h postexposure (dose‐dependent)	Anorexic response: ↓ night‐time food intake (dose‐dependent) ↓ Core body temperature, blood glucose levels and energy expenditure Inflammation: ↑IL‐1B, ↑IL‐6, ↑TNFα, COX‐2, mPGES‐1 expression in liver and spleen ↑IL‐1B plasmatic levels
**MNPs**				
Liang et al. (2025)	**PS‐NPs** (60 nm, spherical, FITC or PE‐labeled or nonfluorescent PS) Model: C57BL/6J mice (male, 13‐weeks‐old) Exposure and Duration per group: Group A – 100 mg/kg bw # oral gavage FITC‐PS NPs for 15 days Group B – gastrointestinal injections of PS‐NPs (200 µg), A53T αS (12.5–150 µg) w/ or wot PS‐NPs (200 µg) Group C – same as B; 3 months postinjection follow‐up gavage of PS‐NPs (2 mg/kg bw, every other day, up to 3 months)	Group A: Confirmation of FITC−PS NPs penetration into the intestinal mucosa and localization within the myenteric and submucosal plexuses Group B: PS‐NPs distributed across the intestinal wall and reached the ENS PS‐NPs + A53T αS coexposure enhanced α‐syn retention and promoted its spread toward the DVC	Group B (3 months postinjection): Anxiety‐like behavior and motor deficits in αS and αS + PS groups, but not with PS‐NPs alone Exacerbation of these effects after reinitiating oral PS‐NPs (group C) Group C: At 4.5 months: ↓ Motor performance only in αS and αS + PS‐NPs groups At 5.5 months: ↓↓ Motor performance in all groups (PS‐NPs + A53T αS more pronounced vs. αS alone; no differences in spatial memory At 6 months: ↓ Dopaminergic neurons in SNpc in all groups;	Group B: ↓ Fecal pellet output in A53T αS‐treated groups (±PS‐NPs); PS‐NPs alone had no effect PS‐NPs ± A53T αS induced apoptosis in the upper duodenum and inflammatory cell infiltration
Li et al. (2024)	**PS‐NPs** (100 nm) Model: C57BL/6J mice offspring (male; maternal exposure) Exposure: Oral gavage (mother) for 32 days 50 µg/day (DGD0 to PND14) Control	Gene expression: altered expression in calcium signaling, neuroactive ligand–receptor interaction, and glutamatergic synapse pathways → developmental reprogramming of the gut–brain axis KEGG Analysis: enrichment of 7 dopamine‐related and 2 serotonin‐related pathways, indicating broad disruption of enteric neurotransmitter signaling	No deficits in offspring locomotion, anxiety behavior, learning and memory of offspring Hippocampal histology showed ↑ nuclear pyknosis, vacuolization, and intercellular spaces in the dentate gyrus Neuroinflammation: ↑ microglial density (no change in neuron count); 1.43‐fold ↑ *TNF‐α* Neurotransmitters imbalance: Disrupted tryptophan and tyrosine metabolism; ↑ dopamine (2.05‐fold), ↓ DOPAC (0.73‐fold)	Impaired intestinal barrier integrity, ileal inflammation (↑ ROS in mucosa, ↓villus height, ↓mucin protein, ↓ *ZO‐1* expression) Gut microbiota: Compositional shift toward a pro‐inflammatory, mucin‐degrading profile ↓ *Patescibacteria*, *Candidatus_Saccharimonas*, ↑ *Verrucomicrobiota*, *Akkermansia*;
Lee et al. (2022)	**PS‐MPs** (2 µm; red fluorescent)Model: C57BL/6J mice (male/ female, adult)Nonoperated animals Subjected to truncal vagotomy Exposure: Oral gavage Group A – 0.008 mg/g bw/twice wk for 4 weeksGroup B – 0.016 mg/g bw/twice wk for 8 weeks Group C – 0.008 mg/g bw/twice wk for 4 weeks, followed by truncal vagotomy and continued PS‐MPs 4‐week exposure Control/Sham control	Group C: memory deficits caused by PS‐MPs were prevented → vagal signaling mediates PS‐MPs‐induced hippocampal dysfunction	Group A: Early molecular alterations: male mice showed ↓ *Egr1*, *Arc* (hippocampus) Synaptic proteins unchanged No behavioral deficits Group B: No alterations in anxiety and sociability Male‐specific hippocampal memory impairment Immediate early gene expression: ↓*Arc*, *Egr1*, *cFos* (dentate gyrus, CA2/3) Synaptic proteins: ↑ *GluA1*, ↓ *Arc* Neuroinflammation: ↑*Iba1*, *TNF‐α*, *IL‐1β* ↑ BBB permeability; PS‐MPs in brain	Group B: ↓ Colon length PS‐MPs detected in the liver

**Abbreviations**: α‐syn, α‐synuclein; AChE, acetylcholinesterase; AgRP, Agouti‐related peptide; ARC, arcuate nucleus; BuChE, butyrylcholinesterase; bw, body weight; CART, cocaine‐ and amphetamine‐regulated transcript; CAT, catalase; CGRP, calcitonin gene‐related peptide; CMMC, colonic migrating motor complex; DMT1, divalent metal transporter 1; DMV, dorsal motor nucleus of the vagus; DOPAC, 3,4‐dihydroxyphenylacetic acid; FBs, fumonisins; Fpn, ferroportin; GAL, galanin; GALR, galanin receptor; GD, gestational day; GFAP, glial fibrillary acidic protein; GST, glutathione S‐transferase; HD, high group; IL, interleukin; iNOS, inducible nitric oxide synthase; IR, immunoreactivity; ISP, inner submucosal plexus; LD, low dose; L‐DOPA, levodopa; L‐NAME, N‐nitro‐L‐arginine methyl ester; MP, myenteric plexus; mPGES‐1, microsomal prostaglandin E synthase‐1; NADH‐d+, nicotinamide adenine dinucleotide diaphorase‐positive; NO, nitric oxide; nNOS, neuronal nitric oxide synthase; NPY, neuropeptide Y; Nrf2, nuclear factor erythroid 2‐related factor 2; NRG1, neuregulin 1; n.s., not significant; NTS, nucleus tractus solitarius; OSP, outer submucosal plexus; PACAP, pituitary adenylate cyclase‐activating polypeptide; PAS, periodic acid–Schiff; PGP9.5, protein gene product 9.5; PND, postnatal day; POMC, pro‐opiomelanocortin; SbP, substance P; Slc30a10, solute carrier family 30 member 10; SNpc, substantia nigra pars compacta; SOD, superoxide dismutase; SP, submucosal plexus; TH, tyrosine hydroxylase; Tmax, time to maximum concentration; UCP‐2, uncoupling protein 2; UCV, unilateral cervical vagotomy; VChAT, vesicular choline acetyltransferase; VIP, vasoactive intestinal peptide.

### 3.4 Characteristics of the Included Studies

The 67 included reports were published between 2006 and 2025, with over half published from 2020 onward, reflecting a growing and recent interest in the topic. While 54 reports investigated a single FC, eight assessed two FCs, one assessed 18 mycotoxins, and four a mycotoxin mixture. From the 67 reports included, a total of 49 studies were identified, each defined as the exposure to a given FC within an animal cohort/experimental design, as detailed in Section 2.4.

#### 3.4.1 Food Contaminants and Additional Experimental Factors

**FIGURE 4:**
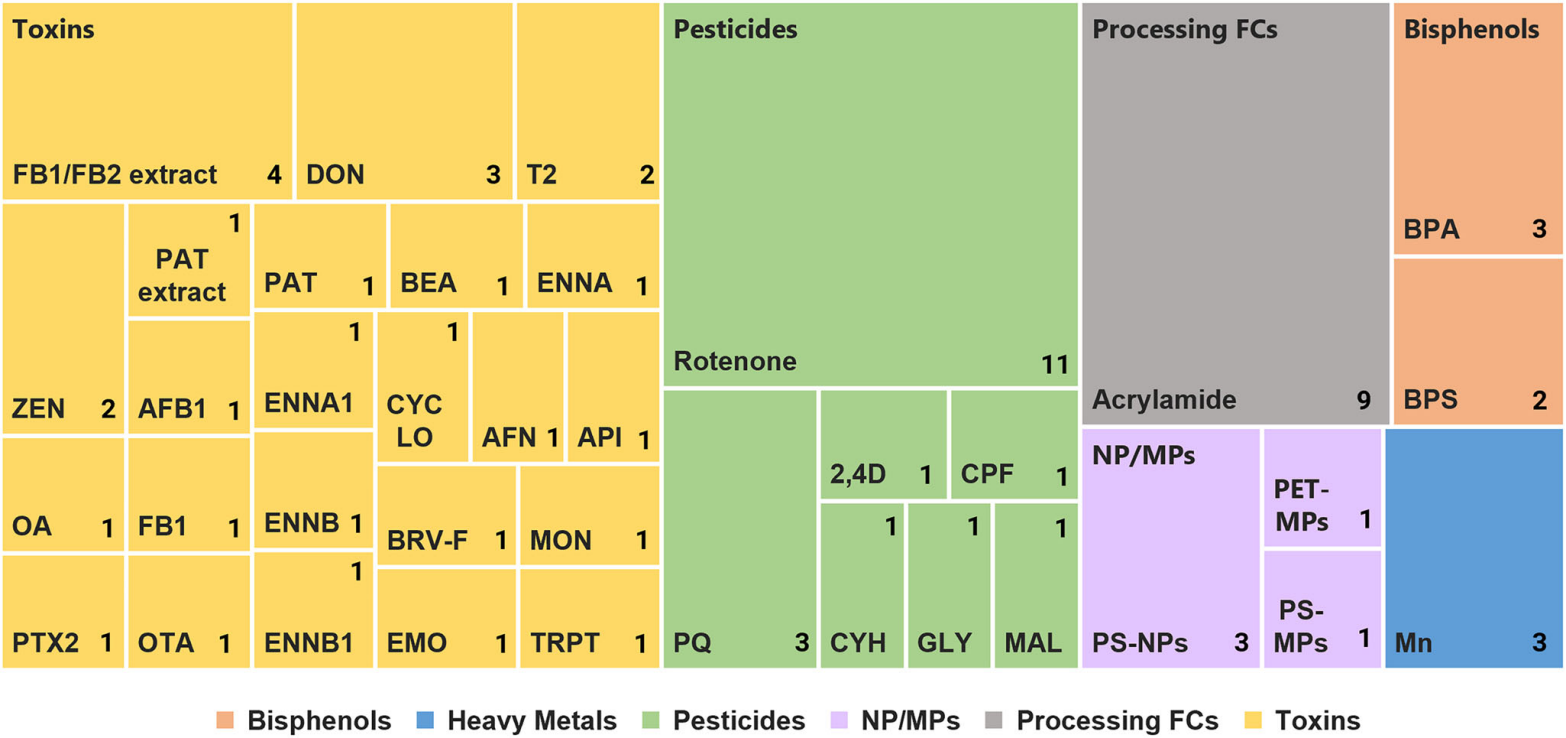
Treemap plot showing the distribution food contaminants (FCs) evaluated in the included studies. FCs are color‐coded by class: pesticides (green), bisphenols (orange), acrylamide (gray), toxins and fungal extracts (yellow), plastics (purple), and heavy metals (blue). The number is each square corresponds to the frequency of studies addressing that compound. 2,4‐D, 2,4‐dichlorophenoxyacetic acid; AFB1, aflatoxin B1; API, apicidin; AFN, aurofusarin; BEA, beauvericin; BPA, bisphenol A; BPS, bisphenol S; CYCLO, Cyclo‐(L‐Pro‐L‐Tyr); CPF, chlorpyrifos; CYH, cyhalothrin; DON, deoxynivalenol; EMO, emodin; ENN, enniatin; FB1/FB2, fumonisin B1 and B2; GLY, glyphosate; MAL, malathion; MON, moniliformin; OA, okadaic acid; OTA, ochratoxin A; PAT, patulin; PET‐MPs, polyethylene terephthalate microplastics; PQ, paraquat; PS‐MPs, polystyrene microplastics; PS‐NPs, polystyrene nanoplastics; PTX2, pectenotoxin‐2; TRPT, tryptophol; T2, T‐2 toxin; ZEN, zearalenone.

A total of 21 unique test compounds from six distinct FC classes were included. Pesticides represented the largest group, including rotenone, paraquat, glyphosate, 2,4‐dichlorophenoxyacetic acid, chlorpyrifos, λ‐cyhalothrin, and malathion. Bisphenols were represented by bisphenol A and bisphenol S. Acrylamide was the only processing contaminant assessed, while manganese represented heavy metals. Toxins comprised mycotoxins and marine biotoxins. The mycotoxin group included T‐2 toxin, deoxynivalenol, zearalenone, patulin and (commercial standard and derived from fungal extract), fumonisin B1, mixtures of fumonisins B1 and B2 (FB1/FB2, derived from fungal extract or culture medium), as well as ochratoxin A, beauvericin, enniatins A, A1, B, and B1, moniliformin, aurofusarin, emodin, apicidin, cyclo‐(L‐Pro‐L‐Tyr), and tryptophol. Marine toxins included okadaic acid and pectenotoxin‐2. Finally, plastic‐related contaminants comprised polystyrene micro‐ and nanoplastics (PS‐MNPs) and polyethylene terephthalate microplastics (PET‐MPs). The overall distribution of FCs studied is illustrated in a treemap plot (Figure 4). Test compounds were obtained from diverse sources, including commercially available analytical standards, extracts from fungal cultures, or in some cases were not specified, with varying levels of physicochemical characterization across studies (Table S4).

In addition, some studies examined interactions between FCs and dietary factors or biochemical modulators. For instance, coffee polyphenols (caffeic acid, chlorogenic acid) were tested against rotenone‐induced ENS toxicity in mice (Miyazaki et al. 2019). Paraquat was coadministered with dietary lectins in rats, hypothesized to enhance uptake and propagation of neurotoxic signals via enteric nerves (Anselmi et al. 2018). Lectins are carbohydrate‐binding proteins commonly found in uncooked legumes and grains, capable of interacting with gut epithelial and neuronal membranes, penetrating the intestinal barrier, and potentially facilitating the translocation of coadministered substances (Anselmi et al. 2018). The impact of PS‐NPs was evaluated in coexposure with A53T α‐syn, a mutant form of α‐syn that enhances the propensity of protein misfolding and aggregation, in a rat EGC line and in C57BL/6J mice via gastrointestinal injections (Liang et al. 2025).

#### 3.4.2 Experimental Models and Experimental Design

In total, 15 in vitro and two ex vivo studies (Table 2), and 34 in vivo studies (Tables 3 and 4), were identified across the included reports.

##### 3.4.2.1 In Vitro and Ex Vivo Models

Primary cell cultures were the most frequently used model type (Lourenssen, Miller, and Blennerhassett 2009; Pan‐Montojo et al. 2012; Arnhold et al. 2016; Guan et al. 2017; Virga, Capps, and Vohra 2018; Brand et al. 2019; Miyazaki et al. 2019; Ghaisas et al. 2021), followed by cell line‐based models (Ghaisas et al. 2021; Reale et al. 2021; Dabrowski et al. 2025; Liang et al. 2025) and ex vivo preparations (Diss et al. 2016; Sharrad et al. 2017). The contaminants investigated in these systems included acrylamide, manganese, paraquat, rotenone, PS‐NPs, pectenotoxin‐2, okadaic acid, and 18 mycotoxins. Across models, rotenone was the most frequently tested contaminant, while manganese was unique in being evaluated in both neurons and glia.

Primary cultures comprised either neuron‐enriched preparations or mixed enteric cultures containing neurons and glia, as well as sympathetic neurons isolated from embryonic or juvenile rodent gut tissue (Lourenssen, Miller, and Blennerhassett 2009; Pan‐Montojo et al. 2012; Arnhold et al. 2016; Guan et al. 2017; Virga, Capps, and Vohra 2018; Brand et al. 2019; Miyazaki et al. 2019; Ghaisas et al. 2021). Virga et al. (2018) compared rotenone toxicity in immunoselected p75NTR^+^ enteric neural crest‐derived neurons and mixed embryonic enteric cultures from CD1 mice, and further employed lentiviral overexpression of cytNmnat1 (cytosolic nicotinamide mononucleotide adenylyltransferase 1, an inhibitor of axon degeneration) and Bcl‐xl (B‐cell lymphoma‐extra large, an antiapoptotic protein) in primary enteric neurons, thereby enabling mechanistic interrogation of FC‐induced neurotoxicity. Pan‐Montojo et al. (2012) established primary cultures of neonatal enteric neurons, transduced with lentiviral mCherry‐α‐syn, and used coculture systems with non‐neuronal cells or sympathetic neurons (direct contact and Campenot chambers) to model disease‐relevant cell–cell transfer (Pan‐Montojo et al. 2012).

Cell line studies focused exclusively on EGCs, derived either from rat (ATCC, CRL2690; Ghaisas et al. 2021; Reale et al. 2021; Dabrowski et al. 2025) or mouse (Liang et al. 2025). EGCs were used in transwell‐based model designed to mimic in vivo intestinal‐glial interactions (Reale et al. 2021). This system incorporated a differentiated coculture of Caco‐2 and HT29‐MTX cells (3:1 ratio, 23 days postseeding) forming an epithelial monolayer on the insert, with rat EGCs cultured in the basolateral compartment and exposed indirectly via apical toxin application, simulating luminal exposure and epithelial‐mediated signaling (Reale et al. 2021).

Ex vivo organotypic gut preparations, preserving physiological architecture, were employed using mouse distal colon (Diss et al. 2016) and guinea pig ileum (Sharrad et al. 2017).

###### 3.4.2.1.1 Exposure Conditions and Outcomes Assessed in Cellular Models

Most studies applied dose–response treatments (often spanning nanomolar to millimolar concentrations) and assess short‐term effects, typically after 24 h of exposure (with some extending to 48 h or multiple days). Endpoints assessed in cellular models fell into six main categories: (i) viability and cell death, (ii) mitochondrial and redox function, (iii) neuronal and glial morphology, (iv) inflammatory and molecular signaling, (v) neurotransmission, and (vi) barrier and tissue‐level functions. Methodological details and specific markers for each study are provided in Table 2.

##### 3.4.2.2 Animal Models

Pigs, rats, and mice were the main species used across the included studies (Tables 3 and 4). Porcine models included Danish Landrace, Pietrain × Duroc, and White Polish pigs, while rodent models comprised Wistar and Sprague‐Dawley rats. Mouse studies predominantly used standard laboratory strains such as C57BL/6J, C57BL/6N, CD1, and DBA/1lacJ, as well as several transgenic lines. Within this group, α‐syn transgenic models were applied to investigate PD‐related mechanisms. SNCA‐overexpressing (SNCA‐OVX) mice, often compared with SNCA‐null counterparts, carry the human SNCA transgene and exhibit age‐related dopaminergic neuron loss and impaired motor coordination (Ahn et al. 2020). A53T α‐syn mice (B6; C3‐Tg‐Prnp/SNCA*A53T/83Vle/J) progressively develop motor deficits from 8 to 15 months of age, with widespread α‐syn inclusions in the spinal cord, brainstem, cerebellum, and thalamus, closely resembling familial α‐synucleinopathies (Naudet et al. 2017; McQuade et al. 2021). In addition to α‐syn models, POMC‐Tau‐Topaz GFP mice (B6.Cg‐Tg(Pomc‐MAPT/Topaz)1Rck/J) were employed to study anorexia‐related mechanisms (Girardet et al. 2011; Gaige et al. 2014). These mice express a reporter construct fusing tau (MAPT) with Topaz GFP under the pro‐opiomelanocortin (POMC) promoter, which drives expression in neurons that generate anorexigenic peptides regulating appetite and energy balance. This allows highly specific visualization of POMC neurons via GFP fluorescence.

###### 3.4.2.2.1 Exposure Conditions

All included in vivo studies employed enteral exposure routes, predominantly through food, drinking water, capsule‐based administration (porcine studies) or oral gavage. Only one study reported gastrointestinal injections, applied alone or in combination with gavage. Full details on exposure routes and administration protocols for each animal study are provided in Table S4.

**FIGURE 5:**
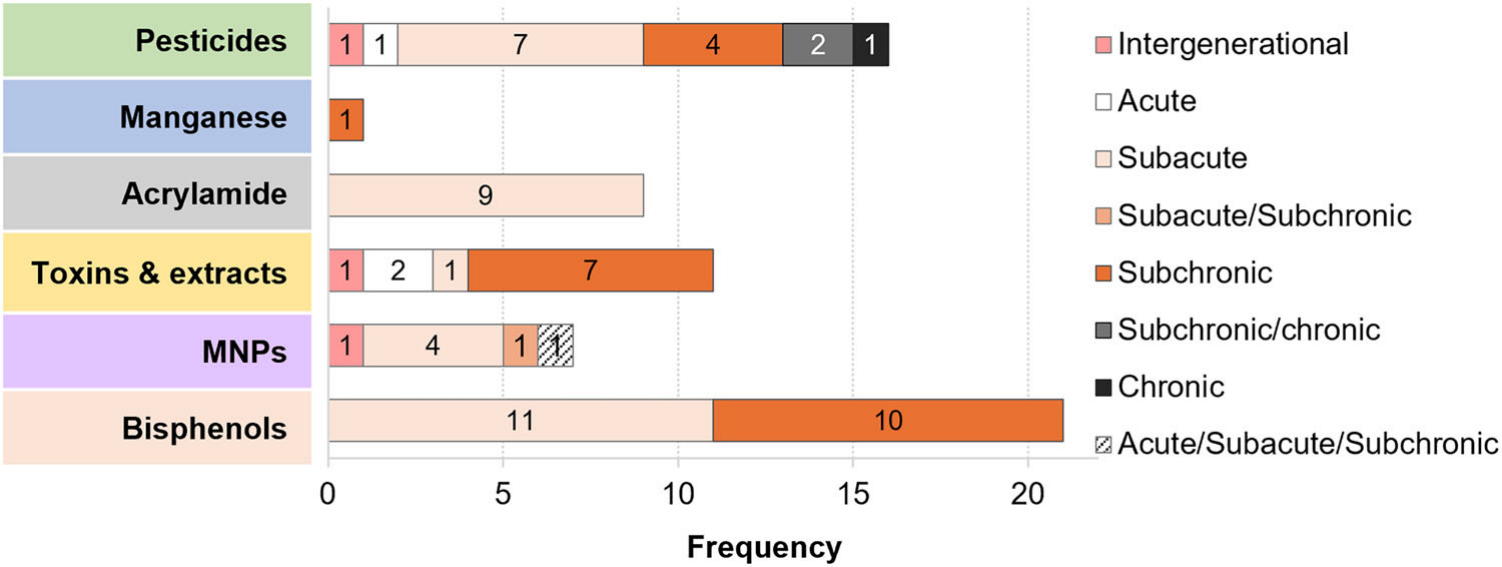
Distribution of FC evaluations according to the duration of exposure in animal studies. Bars represent frequency counts, categorized by duration of exposure/type: intergenerational (maternal exposure with outcome assessment in offspring), acute (24 h), subacute (up to 28 days), subchronic (28–90 days), and chronic (> 90 days) exposure.

Regarding exposure duration, most studies employed short‐ to medium‐term protocols (Figure 5). Subacute designs (≤ 28 days) were the most common (*n* = 32), followed by subchronic studies (> 28–90 days, *n* = 22). A small number of studies applied mixed‐duration protocols, allowing evaluation of the temporal progression and persistence of FC‐induced ENS alterations (Tables 3 and 4). Intergenerational and early‐life exposures were also reported, with offspring outcomes assessed following maternal exposure to chlorpyrifos, FBs extracts, and PS‐NPs. Among contaminant classes, pesticides displayed the broadest distribution across exposure durations.

###### 3.4.2.2.2 Outcomes Assessed in Animal Models

The outcomes reported across the included in vivo studies were heterogeneous, encompassing both (i) effects of FCs on the ENS and (ii) investigations addressing ENS–CNS communication.

A total of 22 studies specifically evaluated local ENS outcomes, primarily in porcine models, but also in mice and rats (Table 3). The FCs assessed included acrylamide, bisphenol A, bisphenol S, PET‐MPs, PS‐NPs, manganese, rotenone, glyphosate, chlorpyrifos, 2,4‐dichlorophenoxyacetic acid, malathion, FB1/FB2 extracts, T‐2 toxin, zearalenone, and deoxynivalenol. Full details of the endpoints and target regions examined in these studies are provided in Table S5. These studies targeted several GI regions, most frequently the small intestine (duodenum, jejunum, ileum), but also the stomach and large intestine, with emphasis in the colon. Most studies applied double immunofluorescence staining to quantify alterations in neuronal subpopulations following subacute or subchronic FC exposure. The pan‐neuronal marker PGP9.5 was routinely used as reference, while markers of interest included tyrosine hydroxylase (TH), PACAP, galanin (GAL), nNOS, VIP, cocaine‐ and amphetamine‐regulated transcript (CART), calcitonin gene‐related peptide (CGRP), substance P (SP), and vesicular acetylcholine transporter (VAChT). Several studies from shared porcine cohorts examined distinct neurochemical markers or gut regions, and their results were harmonized for comparison (see Figures S1–S6 for square matrix plots; data processing followed the approach described in Section 2.4). Additional endpoints included neuropeptide colocalization, NADPH‐d histochemistry, histopathological evaluations, and quantification of nerve structures using morphometric or image‐based analyses. At the molecular level, studies employed Western blotting to assess proteins such as iNOS, TNF‐α, ChAT, muscarinic, and dopamine receptors, while ELISA quantified cytokines including IL‐1β, TNFα, and IL‐6. TH and α‐syn protein were also assessed through immunohistochemistry (Arnhold et al. 2016). Gene expression profiling targeted neurotransmission‐ and inflammation‐related genes such as GALR1‐3, SOD1/2, and nitric oxide synthetase (iNOS; Darwiche et al. 2017; Ghaisas et al. 2021; Palus et al. 2024). In addition, biochemical and enzymatic assays measured acetylcholinesterase (AChE; Darwiche et al. 2017) and butyrylcholinesterase activity (Pupim et al. 2023), oxidative stress markers (Rissato et al. 2020), and serum enzymes including aspartate aminotransferase (AST), alanine aminotransferase (ALT), and γ‐glutamyltransferase (GGT). Some studies extended analyses beyond the ENS, incorporating gut microbiota profiling (taxonomic abundances, fecal metabolite analysis; Ghaisas et al. 2021) and functional assays of gut motility, such as stool collection, intestinal transit, fecal pellet output and bead expulsion.

A subset of 12 studies evaluated both ENS and CNS endpoints to investigate gut–brain communication (Table 4). Tested FCs included rotenone, paraquat, *λ*‐cyhalothrin (toxicokinetic study), deoxynivalenol, T‐2 toxin, and PS‐MNPs. Surgical manipulations were applied in some experimental designs (Pan‐Montojo et al. 2012; Gaige et al. 2014; Anselmi et al. 2018; Ahn et al. 2020; Lee et al. 2022). Comparisons between vagotomized and nonvagotomized animals enabled evaluation of vagal signaling in FC‐related toxicity, including PD‐related outcomes (e.g., for rotenone, Pan‐Montojo et al. 2012; Ahn et al. 2020; paraquat, Anselmi et al. 2018; and PS‐MPs, Lee et al. 2022) and anorexia‐related outcomes (for deoxynivalenol, Girardet et al. 2011; T‐2, Gaige et al. 2014). Unilateral approaches included hemivagotomy, in which one vagal branch is transected while the contralateral branch remains intact (Pan‐Montojo et al. 2012; Ahn et al. 2020), and unilateral cervical vagotomy, performed at the neck level to interrupt vagal fibers before thoracic and abdominal branching (Girardet et al. 2011; Gaige et al. 2014). Truncal (subdiaphragmatic) vagotomy was also employed, involving complete transection of both anterior and posterior vagal trunks below the diaphragm, thereby interrupting all abdominal vagal connections (Anselmi et al. 2018; Lee et al. 2022). In addition, sympathectomy, involving removal or lesion of sympathetic ganglia or fibers innervating the GI tract, was applied in direct comparison with hemivagotomy following rotenone exposure, thereby allowing evaluation of the relative contributions of sympathetic versus vagal pathways in mediating ENS–CNS communication (Pan‐Montojo et al. 2012). Other studies simultaneously assessed GI and brain endpoints without surgical intervention (Anadon et al. 2006; Pan‐Montojo et al. 2010; Tasselli et al. 2013; Naudet et al. 2017; X. Li et al. 2024; Liang et al. 2025).

Assessed endpoints are summarized in Table S6, which compiles the parameters analyzed across studies investigating ENS–CNS communication. Assessed endpoints included histological and immunohistochemical analysis of the CNS, particularly the dorsal motor nucleus of the vagus (DMV), intermediolateral nucleus (IML), and substantia nigra pars compacta (SNpc). Behavioral assays such as rotarod performance, open‐field activity, and gait analysis (Pan‐Montojo et al. 2010, 2012; Tasselli et al. 2013; Anselmi et al. 2018; Ahn et al. 2020; Lee et al. 2022; Liang et al. 2025) were often combined with CNS endpoints, including dopaminergic neuron counts assessed by TH immunostaining, α‐syn detection (Pan‐Montojo et al. 2010, 2012; Tasselli et al. 2013; Anselmi et al. 2018; Liang et al. 2025) and markers of neuroinflammation in the midbrain and brainstem (Lee et al. 2022; X. Li et al. 2024). Anorexia‐related endpoints were also examined in some studies, including c‐Fos immunohistochemistry, double immunofluorescent labeling of c‐Fos/NUCB2/nesfatin‐1, and gene expression analyses targeting neuropeptidergic and inflammatory pathways (AgRP, CART, MC4‐R, NPY, POMC, IL‐1β, IL‐6, TNF‐α, COX‐2, mPGES‐1; Girardet et al. 2011; Gaige et al. 2014). Some studies also included evaluation of α‐syn accumulation in the ENS (Pan‐Montojo et al. 2010, 2012; Tasselli et al. 2013; Naudet et al. 2017; Anselmi et al. 2018; Ahn et al. 2020; Liang et al. 2025), gut motility assays (e.g., intestinal transit, stool or pellet output, and contractility; Pan‐Montojo et al. 2012; Tasselli et al. 2013; Anselmi et al. 2018; Ahn et al. 2020; X. Li et al. 2024; Liang et al. 2025), and gut microbiota analysis (X. Li et al. 2024). Finally, a subset of studies directly assessed the presence and biodistribution of FCs, using HPLC (Pan‐Montojo et al. 2010; Ahn et al. 2020), Raman spectroscopy (Lee et al. 2022), or fluorescent labeling (Liang et al. 2025).

## 4 Discussion

**FIGURE 6:**
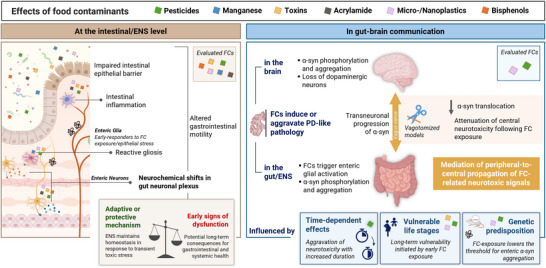
Overview of the effects of food contaminants (FCs) on the enteric nervous system (ENS) and gut–brain communication. Schematic summary of experimental evidence describing the impact of FC exposure at the intestinal/ENS level and in gut–brain interactions. Left panel: At the intestinal level, FCs impair epithelial barrier integrity, induce inflammation, and alter gastrointestinal motility. Enteric glia act as early responders to epithelial or toxic stress, undergoing reactive gliosis, while enteric neurons exhibit neurochemical remodeling within the gut plexuses. These responses may reflect adaptive or protective mechanisms to maintain homeostasis under transient stress or, when persistent, early signs of dysfunction with potential long‐term consequences for gastrointestinal and systemic health. Right panel: In gut–brain communication studies, FCs triggered enteric glial activation and α‐synuclein (α‐syn) phosphorylation and aggregation in the ENS, promoting propagation of α‐syn pathology to the brain through the vagus nerve. Vagotomy models showed reduced central neurotoxicity, supporting the role of vagal pathways in the peripheral‐to‐central transmission of FC‐related neurotoxic signals. The development or severity of these effects is influenced by exposure duration, life stage, and genetic susceptibility. Color codes denote contaminant classes evaluated in the included studies.

To synthesize current evidence on the effects of FCs on ENS and to evaluate the ENS's role in mediating FC‐induced central neurotoxicity, the following discussion is structured around five key thematic domains identified across the reviewed literature: (1) FC‐induced modulation of enteric neuropeptides and GI function; (2) the anorexigenic effects of trichothecenes and the involvement of vagal pathways; (3) the role of EGCs as early responders to oral FC exposure; (4) the potential of FCs to trigger PD‐like pathology in both the ENS and the CNS via GBA; and (5) how ENS responses to FCs are shaped by experimental exposure conditions. Figure 6 provides a representative and integrative overview of the outcomes included, summarizing how FCs can disrupt intestinal and ENS homeostasis and how these local effects may propagate through vagal afferents to influence central neurotoxicity.

### 4.1 FC‐Induced Modulation of ENS Neuropeptides and Gut Function

The ENS exhibits remarkable neurochemical diversity, differentially expressed across neuron types and plexuses, reflecting the specialized functions of each subpopulation (Sharkey and Mawe 2023). These neurotransmitters and modulators enables the ENS to orchestrate gut motility patterns, fluid and electrolyte secretion, mucosal immunity, and microvascular tone in a highly integrated and adaptive manner across different gut regions (Fleming et al. 2020; Montanari et al. 2023; Sharkey and Mawe 2023). For example, GI motility depends on the synchronized interplay of enteric excitatory (cholinergic and SbP) and inhibitory (nitrergic, VIP, NPY, and GAL) neurons which together regulate smooth muscle contraction and coordinated gut motility (Sharkey and Mawe 2023). Exposure to FCs can modulate the neurochemical phenotype of enteric neurons without causing overt neuronal loss (Table 3). Animal studies enabled region‐ and plexus‐specific mapping of neurochemical alterations following FC exposure, summarized in Figures S1–S6.

#### 4.1.1 Pesticides

Cholinergic dysfunction is a hallmark of NDs (Cheng, Ulane, and Burke 2010; Chen et al. 2022). In rats, chlorpyrifos caused sustained inhibition of AChE activity (Darwiche et al. 2017), likely leading to overstimulation of muscarinic receptors, which activates the NF‐κB signaling pathway and induces the overexpression of iNOS (Darwiche et al. 2017; Table 3). The subsequent increase in NO production contributes to a reduction in intestinal smooth muscle contractility via the iNOS–NO axis (Darwiche et al. 2017). Similarly, rotenone reduced cholinergic neurons levels in guinea pig ileum segments (Sharrad et al. 2017; Table 2). Glyphosate exposure at the acceptable daily intake (ADI) level (0.5 mg/kg/day; European Food Safety Authority et al. 2023), increased inhibitory and modulatory neuronal populations in the colon, while the small intestine showed a dose‐dependent rise in GAL‐immunoreactive neurons, most pronounced in the ileum. Jejunal plexuses exhibited increased CART‐, CGRP‐, and nNOS‐positive neurons alongside reduced cholinergic neurons, indicating a shift toward inhibitory and sensory signaling (Bulc, Calka, and Palus 2023; Palus et al. 2024; Palus, Karpiesiuk, and Jana 2025; Table 3 and Figure S1). Although these studies did not assess the effect in gut microbiota, related studies show that even at low doses (1 mg/kg/day) approximating the US ADI level, chronic exposure to glyphosate disrupts gut microbial composition and function, reduces beneficial taxa, impairs short‐chain fatty acids (SCFA)‐related metabolic pathways, and promotes intestinal inflammation (J.B. Liu et al. 2022; Lehman et al. 2023). Given GAL's anti‐inflammatory and microbiota‐modulating roles, its upregulation may reflect an adaptive response to glyphosate‐induced oxidative stress (Brunner et al. 2021).

#### 4.1.2 Acrylamide

In primary myenteric cultures, acrylamide (≥ 0.5 mM; approximately 39 µg/mL) induced axonal damage and suppressed ACh release, yet without altering the proportions of nitrergic (nNOS^+^) or cholinergic (VAChT^+^) neurons, even at 4 mM (Lourenssen, Miller, and Blennerhassett 2009; Table 2). Interestingly, while axon numbers showed signs of regeneration, ACh release remained persistently suppressed, indicating a longer lasting impact on cholinergic neurotransmission than on gross neuronal structure (Lourenssen, Miller, and Blennerhassett 2009). Across animal studies, acrylamide has demonstrated neurotoxic effects across a wide dose range (0.5–50 mg/kg bw), notably impairing the cholinergic system by reducing AChE activity (EFSA 2015; Kopanska et al. 2022). In mice, intraperitoneal administration of 20–40 mg/kg bw significantly decreased AChE activity in both the hypothalamus and intestinal smooth muscle (Kopanska et al. 2017). In humans, chronic occupational exposure to high levels of acrylamide has been consistently associated with sensorimotor neuropathies, including symptoms such as numbness, muscle weakness, cognitive dysfunction, and distal axonal degeneration (Pennisi et al. 2013; Rajeh 2024). The mechanism involves the disruption of cytoskeletal integrity through the downregulation of β‐actin and β‐tubulin, essential proteins for maintaining neuronal structure and axonal transport (Zhao, Zhang, and Deng 2022; Rajeh 2024). This cytoskeletal destabilization contributes to cumulative axonal degeneration and terminal neuropathy (Rajeh 2024). The EFSA CONTAM Panel established a benchmark dose lower confidence limit (BMDL10) of 0.43 mg/kg bw/day for non‐neoplasic effects, based on evidence of sciatic nerve axonal degeneration in rats exposed to acrylamide over 2 years (National Toxicology 2012; EFSA 2015). However, such clinical manifestations are largely attributed to high‐dose exposures, and their relevance to human dietary intake remains limited. Notably, the porcine studies included in this review employed a maximum dose of 5 µg/kg bw/day ‐ 86 times lower than the BMDL10 for non‐neoplasic effects (Palus, Bulc, and Calka 2018a; Palus, Makowska, and Calka 2018b; Palus and Calka 2019; Palus, Makowska, and Calka 2019a; Palus et al. 2019b; Palus, Bulc, and Calka 2020a, 2020b; Bulc, Calka, and Palus 2022; Karpiesiuk, Calka, and Palus 2023; Table 3). Even at these low doses, the subacute acrylamide oral exposure increased expression of neuropeptides such as CART, CGRP, GAL, PACAP, SbP, VAChT, and VIP, along with a reduction in nNOS expression in the jejunum and ileum (Palus, Bulc, and Calka 2018a; Palus, Makowska, and Calka 2018b; Palus and Calka 2019; Palus, Makowska, and Calka 2019a; Palus et al. 2019b; Palus, Bulc, and Calka 2020a, 2020b; Bulc, Calka, and Palus 2022; Karpiesiuk, Calka, and Palus 2023; Figure S2). Additionally, elevated levels of pro‐inflammatory cytokines (IL‐1β, IL‐6, and TNF‐α) were observed in the ileal wall, suggesting that acrylamide may also promote local inflammation (Palus et al. 2019).

While high‐dose (above 1 mg/kg) ACR induces overt neuronal damage and motor deficits, low‐dose exposure (low µg/kg) appears to elicit a multilayered adaptive response. In Crohn's‐like colitis models, nNOS expression in the ENS is significantly reduced, especially in the presence of certain inflammatory cytokines (Winston, Li, and Sarna 2013). In contrast, PACAP exerts protective effects: PACAP deficient mice exhibit—more severe colitis with increased mortality, higher histologic damage, and elevated proinflammatory cytokines (IL‐1β, IL‐6) in the colon (Azuma et al. 2008). These adaptative responses may initially support mucosal defense by preserving epithelial integrity and promoting the clearance of luminal toxicants. However, chronic exposure could shift these responses into maladaptive outcomes, including GI dysmotility, persistent low‐grade inflammation, and neurotoxic GBA signaling. Other studies suggest that ACR may disrupt GBA function by increasing intestinal permeability, altering tight junction integrity, and promoting local and systemic inflammation through elevated and pro‐inflammatory cytokines (Tan et al. 2019). These changes at the intestinal level can impair blood–brain barrier function and contribute to neuroinflammation. Additionally, ACR‐induced gut dysbiosis, characterized by reduced beneficial bacteria and SCFA production, may further exacerbate neurotoxicity via microbiota‐CNS signaling pathways (Xu et al. 2025). Despite these mechanistic insights, human evidence linking dietary ACR exposure to neurotoxicity remains limited. Notably, chronic low‐level dietary ACR intake (∼0.27 µg/day) was associated with cognitive decline over a 4‐year period in a cohort study of elderly Chinese nonsmokers (Z.M. Liu, Tse, et al. 2017) and developmental disabilities in children (Meng et al. 2022).

#### 4.1.3 Microplastics/Nanoplastics

Subacute exposure to PET‐MPs in pigs provoked an overall decrease in cholinergic, nitrergic, as well as VIP neurons in the small, while in the ileum, SbP neurons were increased intestine (Galecka and Calka 2024a, 2024b; Galecka, Szyrynska, and Calka 2024; Figure S4). Histologically, intestinal layers exhibited mucosal thinning and epithelial desquamation, although no assessments of intestinal function or permeability were conducted. Emerging evidence links MNPs to intestinal barrier disruption, immune modulation, and microbiota alterations in experimental models (Agrawal et al. 2024). Additional human evidence has demonstrated the presence of PET‐MPs among the most common microplastics in fecal samples of IBD patients, with concentrations correlating positively with disease severity (Yan et al. 2022). PS‐NPs accumulate in the immature intestine of rats and disrupt epithelial homeostasis, as shown by altered lipid and amino‐acid metabolism and transcriptional changes in mucus‐ and tight‐junction‐related genes indicative of barrier stress (Augustyniak et al. 2025). These effects are accompanied by intestinal inflammation and oxidative damage, while at the ENS level PS‐NP exposure selectively downregulates enteric glial markers without affecting neuronal markers (Augustyniak et al. 2025).

#### 4.1.4 Bisphenols

BPA has recently been banned from food‐contact materials in the EU due to increasing evidence of endocrine and immunotoxic effects (European Commission 2024). In porcine models, BPA at doses of 0.05 mg/kg/day and 0.5 mg/kg/day (corresponding to the 2006 TDI and 10‐fold above it, respectively) caused a dose‐dependent reduction in cholinergic marker expression across enteric plexuses throughout the pig GI tract (Szymanska, Calka, and Gonkowski 2018; Szymanska and Gonkowski 2018; Szymanska, Makowska, and Gonkowski 2018; Szymanska and Gonkowski 2019; I. Gonkowski et al. 2020; Makowska and Gonkowski 2020; Szymanska et al. 2020; Makowska et al. 2021; Makowska and Gonkowski 2022; Figure S6). BPA (0.5 mg/kg/day) also triggered ileal inflammation, including Peyer's patch hypertrophy and mucosal immune cell infiltration, alongside decreased cholinergic and VIPergic neuron populations, and increased nNOS, CART, SP, and GAL expression. The former TDI was derived from a no observed adverse effect level (NOAEL) of 5 mg/kg/day based on multigeneration rodent studies. This dose, along with a lowest observed adverse effect level (LOAEL) of 50 mg/kg, was applied to CD1 mice. After 15 days exposure, both BPA and BPS reduced colonic cholinergic neurons, and interstitial cells of Cajal, without altering enteric glia density (Makowska et al. 2025). A similar reduction in cholinergic neurons was observed after 3 months exposure, although limited to the gastric and jejunal plexuses (Makowska, Lepiarczyk, and Gonkowski 2022; Makowska, Calka, and Gonkowski 2023a; Makowska, Fagundes, and Gonkowski 2023b; Makowska and Gonkowski 2023, 2024; Figure S5). Both bisphenols provoked a broad upregulation of all neuropeptides in the colon, and although neurochemical shifts were dose‐dependent, significant alterations were often observed even at the lower dose (0.05 mg/kg/day) of BPS. Although BPS was introduced as a safer alternative to BPA, it exerted comparable or even greater neuroactive effects. Regulatory assessments have identified a lowest NOAEL of 20 mg/kg bw/day for developmental toxicity and 60 mg/kg bw/day for general systemic toxicity, with no effects on fertility or developmental neurotoxicity observed at doses up to 180 mg/kg bw/day (FitzGerald et al. 2020). However, a growing body of experimental evidence indicates biological activity at substantially lower exposure levels, with adverse effects on neurobehavioral, reproductive, and metabolic endpoints reported at doses in the low µg/kg bw/day range (0.5–10 µg/kg bw/day; Beausoleil et al. 2022). Beyond inducing enteric modulation herein reviewed, evidence suggests bisphenols can alter intestinal permeability, decrease the mucus production, change the composition and diversity of gut microbiota, trigger the recruitment of immune cells or stimulate the production of cytokines (Zhu et al. 2024). A recent study demonstrated that chronic exposure to low doses of BPS (1.5 µg/day) led to marked gut microbiota dysbiosis and significant intestinal damage in mice, including epithelial erosion, crypt disorganization, and inflammatory infiltration (Y. Chi et al. 2024). This dose discrepancy is particularly relevant in light of toxicokinetic data showing that BPS exhibits substantially higher oral bioavailability than BPA, resulting in a greater proportion of the active, nonconjugated compound reaching systemic circulation (Gayrard et al. 2019).

#### 4.1.5 Toxins

Although extensively reviewed elsewhere (S. Gonkowski, Gajecka, and Makowska 2020), mycotoxins also induce distinct neurochemical remodeling within the ENS. Subchronic exposure to T‐2 toxin (12 µg/kg bw/day for 42 days) in pigs increased the proportion of neurons immunoreactive to VIP, CART, and CGRP across myenteric and SPs of the stomach, duodenum, and colon (Figure S3; Makowska et al. 2017; Makowska, Obremski, and Gonkowski 2018; Rychlik et al. 2020). These peptides regulate motility, secretion, vasodilation, and neuroimmune signaling, and their upregulation is consistent with a neuroprotective or anti‐inflammatory adaptation to T2‐induced mucosal stress (Makowska et al. 2017; Makowska, Obremski, and Gonkowski 2018; Rychlik et al. 2020). Similarly, zearalenone elevated CGRP, SP, VIP, and PACAP expression while reducing GAL immunoreactivity in porcine intestinal plexuses and modified colocalization patterns of CGRP with other transmitters (Makowska et al. 2017). In rodents, exposure to fumonisins (FB1/FB2) extracts or deoxynivalenol caused minor reductions in ganglionic or neuronal size at µg/kg doses (Sousa et al. 2014, 2022; Rissato et al. 2020), while changes in neuropeptide markers such as VIP and GAL were only observed at mg/kg exposure levels (Rudyk et al. 2020; Kras et al. 2022).

Globally, FCs consistently induced neurochemical shifts in different neuronal subpopulations of porcine and murine GI tract. Such alterations may reflect adaptive or protective mechanism by which the ENS maintains homeostasis in response to transient toxic stress. Alternatively, they could represent early signs of dysfunction with potential long‐term consequences for GI and systemic health. As most studies rely on short‐term exposures, more work is needed to clarify whether chronic or cumulative exposure leads to sustained neuropeptide dysregulation capable of impairing gut–brain communication or contributing to disease progression.

### 4.2 Trichothecenes Anorexigenic Effects and Vagal Involvement

Trichothecenes, a group of mycotoxins commonly found in contaminated grains, are well‐known for their anorexigenic (appetite‐suppressing) effects, which are mediated by both central and peripheral appetite‐regulating circuits (Terciolo et al. 2018). Evidence suggests that trichothecenes act through distinct mechanisms, engaging either central or vagally mediated pathways, as illustrated by deoxynivalenol and T‐2 toxin studies (Girardet et al. 2011; Gaige et al. 2014).

Acute oral exposure to deoxynivalenol (6.25–25 mg/kg) suppresses food intake by activating central anorexigenic circuits, notably the melanocortin system. This activation is evidenced by increased hypothalamic anorexigenic POMC and CART expression and brainstem c‐Fos activity (Girardet et al. 2011). Interestingly, this effect persists even after vagotomy, suggesting that this mycotoxin can act directly on the brain, possibly via circumventricular organs (Girardet et al. 2011). In contrast, the GBA seems to contribute toward T‐2 anorexigenic effects. Unlike deoxynivalenol, vagal deafferentation significantly attenuated T‐2‐induced c‐Fos expression in the ipsilateral NTS, indicating a critical role for vagal signaling in transmitting the peripheral inflammatory signal (Gaige et al. 2014). Additionally, T‐2 toxin‐induced anorexia in mice may be linked to gut microbiota changes, which can promote the upregulation of GI hormones, neurotransmitters, and pro‐inflammatory cytokines that further contribute to appetite suppression (Huang et al. 2024).

### 4.3 Enteric Glia Act as Early Responders to Oral FCs Exposure

Enteric glia are specialized support cells interspersed among ENS neurons (Sharkey and Mawe 2023; Gonzales and Gulbransen 2025). Once thought to be passive, they are now recognized as active homeostasis regulators responsible for maintaining the neuronal microenvironment, modulating immune responses and supporting the intestinal barrier through cellular crosstalk (Gonzales and Gulbransen 2025).

In this review, multiple FCs were found to induce reactive gliosis (proliferation and hypertrophy of glial cells) in the ENS, commonly evidenced by increased GFAP expression and inflammatory markers (Naudet et al. 2017; Ghaisas et al. 2021; Reale et al. 2021). Rat EGCs also engaged in crosstalk with the intestinal barrier in response to injury (Reale et al. 2021). For instance, the marine toxin PTX2 induced IL‐6 expression in EGCs despite no detectable translocation across the epithelial layer, suggesting an inflammatory glial response to epithelial stress (Reale et al. 2021). In contrast, okadaic acid exposure, which partially crossed the epithelium, stimulated GDNF and BDNF production, likely reflecting a compensatory glial mechanism aimed at supporting mucosal repair and preserving barrier integrity (Reale et al. 2021). Exposure to a broad panel of regulated and nonregulated food‐associated mycotoxins revealed that EGCs are a sensitive and previously under‐recognized target of dietary contaminants (Dabrowski et al. 2025). Specifically, DON, ENNs, BEA, and PAT reached calculated gastrointestinal concentrations, derived from NOAEL, BMDL10, or LOAEL values, that fell within or exceeded antiproliferative or cytotoxic concentration ranges measured in EGCs, indicating a plausible risk to enteric glial function at dietary exposure levels (Dabrowski et al. 2025). In vitro, EGCs were more sensitive to manganese than enteric neurons (Ghaisas et al. 2021), and in vivo Mn exposure led to glial activation and inflammatory response, along with colonic inflammation and mucus depletion (Ghaisas et al. 2021). Paraquat caused early enteroglial activation in both wild‐type and α‐syn transgenic (A53T) mice (Naudet et al. 2017). Notably, only the genetically predisposed mice have increased enteric α‐syn phosphorylation, whereas wild‐type animals showed no α‐syn abnormalities despite clear glial activation (Naudet et al. 2017). These findings suggest that while paraquat can trigger enteric glial reactivity, this response does not influence the development of α‐syn pathology.

However, α‐syn pathology appears to exacerbate the response of EGCs when combined with nanoplastics (Liang et al. 2025). PS‐NPs have been shown to accelerate amyloid aggregation of α‐syn in vitro, indicating a direct interaction that promotes pathological fibrillization (Liang et al. 2025). In EGCs, PS‐NPs induced oxidative stress, mitochondrial dysfunction, and gliosis (Liang et al. 2025). Notably, coexposure to PS‐NPs and mutant α‐syn (A53T) further amplified the glial inflammatory response, as evidenced by increased expression of S100B and elevated levels of key pro‐inflammatory mediators, namely, NO, IL‐6, TNF‐α, COX‐2, and IL‐1β (Liang et al. 2025). These findings suggest that nanoplastics not only enhance α‐syn aggregation but also synergistically amplify EGC‐mediated inflammation.

Colonic biopsies from PD patients reveal increased levels of proinflammatory cytokines such as IL‐6, IL‐1β, and TNFα, alongside gliosis markers (GFAP, S100B; Devos et al. 2013; Clairembault et al. 2014). This proinflammatory glial profile is prominent in prodromal stages and may contribute to intestinal inflammation and early GI symptoms. Although α‐syn aggregation and enteric glial activation frequently co‐occur in the gut of PD patients, current evidence indicates that enteric gliosis does not directly correlate with α‐syn accumulation (Montalban‐Rodriguez, Abalo, and Lopez‐Gomez 2024; Gonzales and Gulbransen 2025). Nevertheless, enteric glial activation in response to prolonged FC exposure may represent a key event in the early stages of PD. By promoting a chronic proinflammatory environment, compromising intestinal barrier function, and altering neuroimmune communication within the ENS, reactive glia may facilitate conditions that predispose the gut to dysfunction (Montalban‐Rodriguez, Abalo, and Lopez‐Gomez 2024; Gonzales and Gulbransen 2025).

### 4.4 FCs Induce PD‐Like Pathology Through Vagal Communication

PD is increasingly viewed as a condition with a substantial environmental etiology (Kalia and Lang 2015; Ben‐Shlomo et al. 2024). Mounting evidence implicates chronic exposure to chemical contaminants, meaning a long‐term constant or intermittent exposure which may have an impact on health over time, as a central driver behind its rising global incidence (Ben‐Shlomo et al. 2024; Dorsey and Bloem 2024).

Rotenone, a mitochondrial complex I inhibitor, is one of the most widely used toxicants to reproduce PD‐like pathology. In mixed enteric and neural crest‐derived cultures, rotenone (10 µM) caused early neurite degeneration preceding neuronal death, with neural crest‐derived cells being more vulnerable (Virga, Capps, and Vohra 2018). Transcriptomic profiling of primary rat enteric neurons identified 75 differentially expressed genes associated with MAPK, Wnt, Toll‐like receptor, and Ras signaling pathways (Guan et al. 2017). In embryonic rat enteric neuron‐glia cocultures, rotenone (1–5 nM) reduced β‐tubulin III and metallothionein expression, effects reversed by pretreatment with caffeic or chlorogenic acid, but only at the highest rotenone dose, suggesting a dose‐dependent neuroprotection role of polyphenols (Miyazaki et al. 2019). In guinea pig ileum cultures, rotenone promoted α‐syn fibril accumulation within cholinergic axons, leading to selective necrosis (Sharrad et al. 2017).

As reviewed by Innos and Hickey (2021), administration route, vehicle, and chemical stability critically determine whether rotenone exposure reproduces slowly progressive PD‐like pathology or merely acute toxicity. Chronic low‐dose oral gavage or subcutaneous minipump infusion recreate gut‐to‐brain α‐syn propagation and nigrostriatal dopamine loss, whereas bolus intraperitoneal or subcutaneous injections yield erratic exposure, high mortality and variable outcomes (Innos and Hickey 2021). Repeated oral gavage of rotenone at 5 mg/kg, 5 days per week, was described as capable of inducing progressive α‐syn phosphorylation and aggregation within the ENS, accompanied by dopaminergic and cholinergic neuron loss and prominent enteric gliosis, even though rotenone remained undetectable in plasma and brain homogenates (Pan‐Montojo et al. 2010; Arnhold et al. 2016; Schaffernicht et al. 2021). After 3 months, pathology subsequently spread to DMV, IML, and SNpc, culminating in dopaminergic cell death and motor impairment (Pan‐Montojo et al. 2010). The absence of systemic rotenone supports a trans‐neuronal, vagus‐mediated transfer of α‐syn pathology rather than direct central exposure, highlighting oral FC exposure as a plausible environmental driver of PD‐like disease. Conversely, high‐dose oral rotenone (30 mg/kg/day, 28 days) caused high mortality and central dopaminergic loss with motor deficits, while the ENS remained largely unaffected aside from reduced jejunal α‐synuclein and decreased fecal output (McQuade et al. 2021).

Paraquat, another pesticide with suspected neurotoxic potential, has been extensively studied and remains a regulatory concern. Although banned in several jurisdictions, including the European Union, it remains extensively used in the United States and many developing regions where regulatory oversight and protective measures are often inadequate (Darweesh, Vermeulen, and Bloem 2024). Current epidemiological evidence increasingly supports an association between paraquat exposure and PD risk (Tangamornsuksan et al. 2019; Paul et al. 2024), particularly in long‐term, high‐intensity exposure and younger onset cases (Paul et al. 2024).

In experimental research, paraquat is also widely used to model PD‐related neurodegeneration. Intraperitoneal injection is the most frequently employed route in rodent studies, repeated over several weeks or months, resulting in progressive dopaminergic neuron loss in the substantia nigra and motor impairments resembling PD (Minnema et al. 2014). However, paraquat's capacity to initiate a gut‐to‐brain propagation of α‐syn pathology appears more conditional compared to rotenone, depending on the presence of genetic or environmental facilitators. As mentioned previously, subchronic oral paraquat exposure (10 mg/kg bw/day) in WT mice provoked enteric glial activation without no pSer129 α‐synuclein accumulation in the ENS or brain (Naudet et al. 2017). In contrast, transgenic TgHuA53T mice, which spontaneously develop α‐syn pathology with age, showed accelerated and intensified ENS pSer129 α‐syn accumulation following the same paraquat regimen, along with pronounced gliosis, though still without CNS pathology or motor deficits (Naudet et al. 2017). This indicates that genetic susceptibility may lower the threshold for paraquat‐induced enteric α‐syn aggregation.

Remarkably, coexposure to paraquat and dietary lectins in rats, despite employing a shorter duration and lower dose regimen, substantially altered the pathological outcomes (Anselmi et al. 2018). Animals exposed to both agents developed phosphorylated α‐syn accumulation not only in myenteric neurons but also in cholinergic and dopaminergic neurons of the DMV and SNpc, accompanied by gastric dysmotility and motor impairments (Anselmi et al. 2018). These effects were not observed with paraquat or lectin alone, indicating a synergistic interaction, likely mediated by increased intestinal epithelial permeability and immune activation. Critically, subdiaphragmatic vagotomy abolished central α‐syn propagation, restricting pathology to the ENS and confirming vagal dependence for ascending transmission (Anselmi et al. 2018).

Emerging evidence indicates that plastic particles can act as environmental neurotoxicant capable of disrupting the GBA communication (Lee et al. 2022; Liang et al. 2025). Subchronic oral exposure to PS‐MPs in WT mice has been shown to induce dysregulation of immediate early gene (IEG) expression, hippocampal inflammation and impaired cognitive function (Lee et al. 2022). These central effects were abolished following subdiaphragmatic vagotomy (Lee et al. 2022). In mice injected intraduodenally with mutant α‐syn, coexposure to PS‐NPs enhanced α‐syn retention in the ENS and facilitated its propagation to the dorsal vagal complex (DVC; Liang et al. 2025). Behavioral assessments over 6 months revealed progressive anxiety‐like behavior, motor impairment, and dopaminergic neuron loss in the SNpc, particularly in coexposed animals. While PS‐NPs alone did not initiate α‐syn aggregation, they significantly amplified pathology and functional decline in predisposed animals.

By disrupting vagal communication, such interventions help establish whether gut‐derived signals are necessary for initiating CNS pathology, thereby clarifying the role of dietary neurotoxicant exposure in PD progression. Collectively, these findings align with Braak's “body‐first” hypothesis, indicating that FCs can initiate PD‐like pathology within the GI tract. The ENS not only manifests early pathological changes in response to FCs but also acts as a critical interface that mediates subsequent neurotoxic effects in the CNS.

### 4.5 ENS Responses to FCs Are Shaped by Experimental Exposure Conditions

The severity and nature of toxicological outcomes resulting from FC exposure are strongly influenced by both the duration and concentration of exposure. Most studies included in this review employed acute or subacute exposure protocols, limiting their ability to capture long‐term effects. In porcine models, short‐term (average 28 days) FC exposure has been shown to elicit adaptive responses in the ENS, underscoring its role as a key regulator of GI function under stress (Table 3). Nevertheless, acrylamide administered at doses up to 5 µg/kg bw/day (approximately 86‐fold below the BMDL10) and glyphosate up to 0.5 mg/kg bw/day (equivalent to the ADI) consistently induce enteric neurochemical remodeling, highlighting the ENS as a particularly sensitive peripheral target of dietary contaminant exposure that responds at low, risk‐assessment‐relevant doses in the absence of overt systemic toxicity.

For mycotoxins, maximum levels in foodstuffs and feedstuffs are established under EU and international frameworks (European Commission 2006b). Although not legally binding, a EU recommendation for piglet feed indicates that zearalenone should not exceed approximately 100 µg/kg to mitigate adverse effects in this species (European Commission 2006a). In the dataset reviewed here, a dietary concentration of 6 µg/kg ZEN elicited a modest increase in CRGP expression in the pig colon (Makowska et al. 2017). Nevertheless, EFSA's CONTAM set a TDI of 0.25 µg/kg bw/day (as ZEN equivalents) based on estrogenic effects in pigs (C. EFSA 2016a). Fumonisins are poorly absorbed across the intestinal epithelium in most species, with oral uptake below 4% of the administered dose (C. EFSA 2018). The EFSA CONTAM Panel (2018) adopted a group TDI of 1.0 µg/kg bw/day for FB1–FB4, derived from a BMDL10 of 0.1 mg/kg bw for hepatocytic effects in rodents (C. EFSA 2018). For FBs, exposure levels in experimental studies are highly variable, spanning approximately 0.1 mg/kg bw to 90 mg/kg bw, with no relevant effects at lower doses (Table 3).

The dosing regimens used for chlorpyrifos in the reviewed studies lack direct relevance for typical human dietary exposure, as they substantially exceed established toxicological reference points. Administration at 1–5 mg/kg bw/day (Darwiche et al. 2017) corresponds to approximately 10–50‐fold above the NOAEL of 0.1 mg/kg bw/day defined for AChE inhibition in both short‐ and long‐term studies in rats and dogs (European Food Safety Authority 2019).

Concerning the dosing regimens of rotenone (1–50 mg/kg bw) and paraquat (1–10 mg/kg bw), most of the reviewed studies employ neurotoxicant doses that substantially exceed realistic human exposure levels and established toxicological reference points. For rotenone, a chronic NOAEL of 0.375 mg/kg bw/day has been identified in rats, while for paraquat the lowest reported chronic NOAEL, derived from a 1‐year oral toxicity study in dogs, is 0.45 mg paraquat ion/kg bw/day (Food Safety Commission of Japan 2022). Accordingly, the doses used in these experimental paradigms exceed regulatory points of departure, indicating that they are primarily designed to induce overt neurotoxicity or disease‐like phenotypes rather than to model environmentally or dietary‐relevant human exposures. While these doses are known to reliably induce hallmark PD‐like pathology in the brain (Tieu 2011; Kulcsarova et al. 2023), they also cause changes in the ENS and gut, reflecting secondary consequences of central toxicity. These outcomes, while mechanistically informative, complicate the interpretation of whether the ENS serves as a primary site of FC toxicity or is secondarily affected following CNS involvement. This distinction is crucial in light of the body‐first hypothesis of PD, which proposes that the disease originates in peripheral sites, namely, the GI tract, and spreads centrally via structures such as the vagus nerve (Braak, Rub, et al. 2003b; Borghammer et al. 2022; Velucci et al. 2025). Given that the gut serves as the first point of contact with orally ingested neurotoxicants, understanding the mechanistic roles of the ENS and vagus nerve interference in mediating the effects of dietary FC exposure and PD, it is essential to use contaminant levels that do not directly induce CNS toxicity through enteral routes of administration. For example, Pan‐Montojo et al. (2010) showed that rotenone exposure was undetectable in systemic circulation below 5 mg/kg bw, and brain accumulation did not occur below 10 mg/kg ‐ suggesting those levels did not produce direct neurotoxicity (Pan‐Montojo et al. 2010). Based on these findings, the authors proceeded with their main experiments using a dose of 5 mg/kg bw (Pan‐Montojo et al. 2010). In contrast, another study assessed rotenone effects at a much higher dose of 30 mg/kg bw (Tasselli et al. 2013). This exposure level led to high mortality, with several animals dying within the first 10 days of treatment (Tasselli et al. 2013). At this dose, rotenone caused pronounced loss of dopaminergic neurons in the SNpc and significant motor deficits, while no alterations in total, cholinergic and nitrergic enteric neurons were evident (Tasselli et al. 2013).

Concerning MNPs, there is currently no specific legislation addressing MPs or NPs as FCs, and human risk assessment remains hindered by major data gaps. Within this context, the studies reviewed here applied PS‐MP doses ranging from 0.625 to 31.25 mg/kg bw, (0.625 and 31.25 mg/kg bw in porcine models (Galecka and Calka 2024a, 2024b; Galecka, Szyrynska, and Calka 2024), and 8 and 16 mg/kg bw in mice (Lee et al. 2022)), while PS‐NPs were administered orally at 1 mg/kg bw in rats (Augustyniak et al. 2025) or 2 mg/kg bw in mice (X. Li et al. 2024). Dietary assessments suggest that European seafood consumers may ingest several thousand MP particles annually, and bottled water consumption adds further exposure (up to 90,000 particles per year), as plastic particles can leach from packaging materials (Schwabl et al. 2019; Song et al. 2025). Human biomonitoring studies detected MPs in 15 different biological compartments including blood, liver, colon, placenta and breastmilk (Kutralam‐Muniasamy et al. 2023). The gut emerges as one of the regions with the most frequent detection and highest levels of MPs, with MPs routinely identified in stool samples averaging around 30 particles/g and detection rates often exceeding 95% of analyzed samples (Schwabl et al. 2019; Zuri, Karanasiou, and Lacorte 2023; Song et al. 2025). Comparable concentrations have also been reported in colon tissue, at approximately 28.1 particles/g (Ibrahim et al. 2021). Keeping in mind the limited number of mass‐based measurements and analytic limitations, recent human exposure estimates based on mass‐concentration measurements suggest that total estimated daily intake may range from 5.98 µg/kg bw/day to as high as 18.7 mg/kg bw/day, with a median of 70.3 µg/kg bw/day, corresponding to approximately 4.9 mg of plastic ingested daily by an average adult (Zuri, Karanasiou, and Lacorte 2023). Moreover, current analytical methods lack the sensitivity and standardization to robustly quantify NPs, although they potentially pose greater risks because their small size facilitates crossing of biological barriers and accumulation in tissues. Reflecting this knowledge gap, EFSA highlights the need for targeted research on the toxicokinetic and toxicity of plastic particles, including investigations of local effects in the GI tract and the potential degradation of MPs into NPs within the human digestive system, to support hazard characterization and future risk assessment (C. EFSA 2016b).

Although the literature is severely limited, available evidence suggests that FCs exert time‐dependent effects (Pan‐Montojo et al. 2010; Lee et al. 2022; Liang et al. 2025). In mice, chronic oral rotenone exposure (5 mg/kg, 5 ×/week) induces progressive α‐syn accumulation starting in the ENS and extending to the IML, DMV, and ultimately the SNpc (Pan‐Montojo et al. 2010). After 3 months, dopaminergic neuron loss and motor deficits are observed, whereas earlier stages show α‐syn pathology without cell loss (Pan‐Montojo et al. 2010). Similarly, subchronic oral PS‐MPs (2 µm) exposure in C57BL/6J mice leads to progressive hippocampal damage (Lee et al. 2022). Molecular changes (e.g., IEG dysregulation) appear by 4 weeks, followed by cognitive impairment, reduced synaptic gene expression, inflammation, and BBB permeability at 8 weeks (Lee et al. 2022). These findings are particularly relevant in the context of dietary exposure, which typically involves sustained, low‐dose ingestion over a lifetime, potentially resulting in cumulative neurodegenerative effects.

In the reviewed studies, FCs administered during gestation or lactation also induced significant neurochemical, morphological and functional changes in the developing GBA (Darwiche et al. 2017; Kras et al. 2022; X. Li et al. 2024). Chlorpyrifos exposure from gestation until early adulthood causes a shift in ENS neurotransmission from cholinergic to nitrergic signaling, linked to intestinal hypomotility (Darwiche et al. 2017). Maternal FB exposure leads to increased VIP and GAL expression without neuronal loss, indicating a compensatory response to early‐life stress (Kras et al. 2022). Maternal PS‐NPs exposure (50 µg/day until PND14) impairs intestinal barrier integrity, triggers ileal inflammation, and alters microbiota toward a pro‐inflammatory, mucin‐degrading profile (X. Li et al. 2024). ENS gene expression is disrupted, particularly in DA and serotonin (5‐HT) pathways, consistent with GBA reprogramming (X. Li et al. 2024). These alterations extend centrally, with observed neuroinflammation, cortical and hippocampal damage, and neurotransmitter imbalances in offspring, suggesting long‐term vulnerability initiated by early FC exposure (X. Li et al. 2024). Early‐life exposure, particularly during prenatal and postnatal development, represents a window of heightened vulnerability (Sarron et al. 2020). Human breast milk is a well‐established route for transferring persistent environmental pollutants from mother to infant, especially in the early postnatal phase when the infant's gut and immune systems are still immature. Many of the environmental chemicals detected in breast milk, including heavy metals, PCBs and organochlorine pesticides, originate from maternal dietary intake (Mead 2008). These contaminants can accumulate in maternal adipose tissue and be released into milk at higher concentrations during early lactation (particularly in colostrum) posing significant exposure risks during the infant's most sensitive developmental window. This stage involves a complex interplay between the gut microbiota, the mucosal immune system, and the neuroendocrine network (Sarron et al. 2020). Disruption by FCs during this phase can impair the proper programming of gut and neurodevelopmental function, potentially shifting health trajectories toward increased disease susceptibility (Sarron et al. 2020). Immunotoxicants like dioxin‐like PCBs and certain organochlorines have been linked to altered immune responses, endocrine disruption, and neurodevelopmental alterations (Z.H. Chi et al. 2023; Nermo et al. 2025). Recently, microplastic fragments were found in approximately 75% of human milk samples collected 1 week postpartum (Ragusa et al. 2022). Nevertheless, long‐term monitoring of 513 milk samples from Norwegian mothers offers some reassurance, as concentrations of key persistent pollutants such as β‐HCH, DDTs, PBDEs, and PCBs in breast milk have declined significantly (17% annually) over the past two decades, reflecting the effectiveness of international regulatory actions (Nermo et al. 2025). These findings collectively justify calls for intensified biomonitoring and preventive policy to protect early‐life chemical exposure.

## 5 Translation Applicability and Methodological Limitations of the Included Studies

In this systematic review, the quality assessment of the included studies using ToxRTool revealed some methodological shortcomings. A recurrent issue was the insufficient detail regarding the purity and origin of the tested substances. This limitation is particularly relevant for mycotoxins, which may be isolated from fungal cultures or acquired as purified analytical standards. In this review, mycotoxins derived from fungal culture media or uncharacterized extracts were categorized as fungal extracts. For example, Brand et al. (2019) report 68% patulin in a crude *P. coprobium* extract (data not included) yet did not provide the purity of the so‐called *purified patulin* derived from that same extract. Similarly, although FB1/FB2 ratios were reported for *F. verticillioides* extracts, comprehensive compositional analysis was lacking in some other studies (Sousa et al. 2014, 2022; Rudyk et al. 2020). Although FB1 and FB2 are the predominant metabolites produced by *F. verticillioides*, particularly by strain MRC 826, other fumonisins such as FB3 and FB4, sharing similar chromatographic properties with FB1, are also known secondary metabolites of this fungus (Gelderblom et al. 1992; Proctor et al. 2006). This raises concerns about the possible presence of additional mycotoxins that may remain undetected and confound toxicological interpretations. In repeated‐dose studies, missing ToxRTool scores were often attributed to the absence of analytical confirmation of the administered doses or a lack of contaminant stability testing. Insufficient information regarding housing or feeding conditions frequently contributed to scoring omissions.

Another identified issue relates to anatomical and physiological interspecies differences, which must be considered when interpreting outcomes and judging translational relevance. As reviewed by Sharkey and Mawe (2023), the core neurochemical subtypes and functional classes of enteric neurons are conserved across humans and commonly used laboratory species, but important differences exist in ENS organization and in the gastrointestinal tract exist (Sharkey and Mawe 2023). Beyond anatomy, emerging molecular and functional evidence supports the pig as a strong translational model. Comparative profiling of porcine and human myenteric ganglia across corresponding colonic regions identified highly conserved regional programs, and importantly, vagus nerve stimulation in pigs predominantly affected myenteric ganglia with regional responses that shared more than 96% of the conserved core transcriptional programs between pig and human (T. Li et al. 2023). For example, mechanosensitive enteric neurons were demonstrated in the ISP of both porcine and human colon using neuroimaging approaches, with comparable spike discharge to compressive and tensile stimuli and a similar neurochemical code (Filzmayer et al. 2020).

Rodents display markedly faster gastrointestinal transit, often completing passage from ingestion to defecation within hours, whereas human transit typically spans a day or longer. Pig transit times are closer to those observed in humans (Rose, Blikslager, and Ziegler 2022). When normalized to body weight, total gut length also differs substantially across species, being longest in pigs at approximately 24 cm/kg and shorter in humans at around 14 cm/kg (Hatton et al. 2015). Gut microbiota composition also diverges substantially between species, with marked differences between mice and humans. A large proportion of bacterial genera present in the mouse gut are not detected in humans, whereas pigs and humans share more similar dominant adult phyla, particularly Firmicutes and Bacteroidetes (Rose, Blikslager, and Ziegler 2022).

Thus, the included porcine studies provide high‐resolution mapping of ENS neurochemical remodeling in a system that closely mirrors human enteric anatomy and organization, supporting their translational relevance for identifying FC‐induced alterations at the plexus and neuronal subtype level. Nevertheless, the interpretation of differences in enteric neuronal subpopulations following FC exposure also requires caution. Several reports did not account for the baseline proportions of each neuronal subpopulation in control animals, giving equal weight to changes in low‐abundance neurochemical markers as to those that are highly represented. This may overstate the biological relevance of certain findings and complicates the interpretation of selective vulnerability within the ENS. Our approach to these studies ensured that such alterations were appropriately scaled, providing a more biologically meaningful interpretation of contaminant‐induced neurochemical changes (Figures S1–S6). Although this approach improves comparability and emphasizes potentially meaningful biological effects, it carries certain limitations. For example, the calculation of fold changes was based on reported group means, which limits the statistical precision.

Moreover, most included studies focused narrowly on enteric tissue endpoints without addressing functional consequences in gut physiology, microbiota, or CNS effects, leaving the propagation potential of FC‐induced ENS disturbances underexplored. Notably, this limitation applies uniformly to studies employing the porcine model, despite its close anatomical and physiological resemblance to the human ENS, representing a missed opportunity to leverage a highly translational system for integrated GBA investigations. A neuroscience‐focused review on pigs as translational models argues that pigs offer neuroanatomical advantages (gyrencephalic brain, feasibility of longitudinal work) but also emphasizes that further work is needed to strengthen validity for NDs research, including limitations in behavioral readouts and the relative immaturity of standardized porcine ND paradigms compared with rodents (Hoffe and Holahan 2019).

In the context of clinical PD, evidence linking ENS neurochemical remodeling to human pathology remains limited (O'Day et al. 2022; Templeton, Tobet, and Schwerdtfeger 2025). Across colonic biopsy and postmortem studies, neurochemical alterations have been reported in pathways that are mechanistically plausible for PD constipation, including catecholaminergic markers (e.g., TH and dopamine‐related enzymes) and neuropeptidergic signaling such as VIP, but results are inconsistent across cohorts and are often difficult to separate from constipation itself, sampling depth, and plexus‐specific vulnerability (O'Day et al. 2022; Templeton, Tobet, and Schwerdtfeger 2025). A critical synthesis of the human literature emphasizes that evidence for enteric neuron loss or subtype‐specific depletion in PD is heterogeneous, frequently underpowered, and variably controlled for constipation and other confounders, limiting inference on whether changes reflect degeneration, phenotypic switching, or compensatory remodeling (O'Day et al. 2022). Importantly, functional human data argue against a uniform ENS deficit, in routine duodenal biopsies, live imaging of submucosal neurons showed preserved calcium responses and mitochondrial membrane potential in PD compared with controls, suggesting that PD‐related GI symptoms are unlikely to be explained solely by submucosal dysfunction in the proximal small intestine and reinforcing the need to consider region‐ and layer specific mechanisms (including myenteric circuitry and extrinsic autonomic inputs; Desmet et al. 2017).

Only a limited number of studies assessed central neurotoxicity outcomes alongside FC‐related ENS effects (Table 4). Most focused on molecular or histopathological markers, including α‐synuclein accumulation (particularly pSer129‐α‐syn), dopaminergic endpoints such as TH immunoreactivity or neuron counts, and glial activation markers, and functional measures such as motor performance or locomotor activity (Table S6). Although these endpoints are relevant to human clinical pathology, translational frameworks consistently indicate that molecular and histopathological CNS markers alone, primarily demonstrate pathway perturbation rather than disease‐equivalent neurodegeneration or long‐term human risk (Deepika et al. 2020). Accordingly, animal FC studies should combine CNS pathology with fluid biomarkers and behavioral or physiological endpoints to provide substantially stronger evidence for human relevance than studies relying on single molecular readouts (Roberts et al. 2015). Because overt motor deficits in both humans and animal models arise only after substantial dopaminergic loss, functional endpoints are critical for detecting earlier, subclinical neurotoxicity, can be applied longitudinally, and are less invasive, consistent with OECD and EPA recommendations. Consensus efforts emphasize that fluid‐based biomarkers such as neurofilament‐L, GFAP, and Tau outperform tissue‐only markers in translational value, as they sensitively reflect nervous system injury, correlate with histopathology in nonclinical models, and can be monitored longitudinally in humans (Roberts et al. 2015; Vlasakova et al. 2023). Critically, acute or high‐dose paradigms commonly used in nonclinical testing may exaggerate effect sizes relative to environmentally relevant exposures, limiting their direct applicability to human neurodegenerative risk assessment (Deepika et al. 2020).

Finally, acknowledging the limitations of this systematic review is also essential for contextualizing the interpretation of the findings. FCs represent only one component of the broader human exposome, which encompasses a wide range of environmental, occupational, and lifestyle‐related chemical stressors that may interact across the life course. As such, the effects attributed to FCs in isolation should be interpreted with caution, as real‐world exposures typically involve complex mixtures and coexposures. Additionally, although several included studies explored the capacity of dietary factors to modulate FC‐induced effects, this aspect was not the primary focus of the review. The search strategy was therefore not explicitly designed to exhaustively capture the extensive literature on diet‐contaminant interactions, nutritional modifiers, or food matrix effects.

## 6 Research Gaps and Future Directions

A critical appraisal of the current evidence highlights several unresolved questions and significant research gaps, primarily centered around two major challenges: (1) the accurate translation of the complex and dynamic human exposome into research frameworks, and (2) the advancement of experimental models to investigate the ENS and GBA more effectively.

### 6.1 Translating the Human Chemical Exposome Into Research

Most available studies have focused on a narrow subset of FCs, often under acute or high‐dose exposure conditions. Given the pervasive, lifelong dietary exposure of the general population to low levels of multiple chemical contaminants (Sarron et al. 2020; Lefevre‐Arbogast, Chaker, et al. 2024), further research is necessary to determine whether chronic exposure levels consistently reach biologically relevant thresholds capable of inducing neurotoxicity, to elucidate the specific mechanisms involved and assess realistic mixtures toxicity. In fact, the effects of most well‐known FCs on the ENS remain largely unknown. While pesticide‐based PD models provide a proof‐of‐principle that exogenous compounds can selectively target and degenerate substantia nigra dopaminergic neurons and provoke α‐syn pathology, highlighting a causal role for environmental toxicants in PD pathogenesis (Kulcsarova et al. 2023), much less is known about their impact on the enteric and peripheral nervous systems (Kulcsarova et al. 2023). Similarly, one of the most pressing research gaps is the scarcity of studies evaluating the effects of heavy metals on ENS, despite their well‐established central neurotoxicity (Bakulski et al. 2020; Porru et al. 2024). Metals such as Pb, Hg, Cd, and As are linked to gut dysbiosis, which correlates with neuropsychological outcomes like anxiety, depression, and cognitive impairment (Porru et al. 2024). Additionally, several heavy metals can bind to α‐syn, promoting its misfolding, oligomer stabilization, and impaired clearance, processes implicated in PD progression (Carboni and Lingor 2015). Interestingly, comparable molecular disturbances have been described for PS‐NPs, suggesting that different FC classes may converge on similar toxicity pathways (Z. Liu et al. 2023; Liang et al. 2025).

Other widely distributed FCs in the food chain, such as ochratoxin A, aflatoxin B1, and citrinin—also recognized for their CNS toxicity—have an unexplored potential to modulate GBA signaling. Critically, most studies have assessed single‐compound exposures, leaving the effects of realistic mixtures of FCs on the ENS and GBA poorly understood. Recent studies are beginning to address this gap through exposomic and mixture‐based approaches. For instance, exposure to a complex mixture of 45 FCs simulating high‐consumer dietary intake markedly enhanced inflammatory and oxidative stress responses in in vitro intestinal models (Ramos et al. 2025). Similarly, realistic plasma‐derived chemical mixtures tested by Braun et al. (2024) demonstrated consistent neurotoxicity despite subthreshold levels of individual components. This is a clear demonstration of the “something from nothing” effect, whereby compounds considered inactive in isolation provoke significant bioactivity when combined. It is particularly relevant in the context of dietary exposure, where long‐term ingestion of complex FC mixtures may initiate or exacerbate gut and brain dysfunction via the GBA.

Another important factor to consider when translating the human chemical exposome into research is dietary compounds, which act as the matrix vector for FC ingestion and may serve as critical modulators of FC‐induced effects. A recent study linked high‐fat dietary patterns to an increased risk of dementia, particularly when coupled with elevated exposure to environmental contaminants such as perfluoroalkyl substance and PFAS, flame retardants, mycotoxins, and nitrites (Lefevre‐Arbogast, Duquenne, et al. 2024). Beyond overall dietary patterns, the physicochemical characteristics of the food matrix itself strongly influence FC stability, release, and bioaccessibility during digestion, thereby shaping internal exposure levels. Food composition, structure, and processing can alter contaminant partitioning, interaction with macronutrients, and gastrointestinal solubilization, leading to exposure profiles that differ substantially from those predicted using isolated compounds (Aguilera 2019; Shahidi and Pan 2022). Other nutritional components such as polyphenols, dietary fiber, and probiotics have demonstrated the ability to counteract contaminant‐induced gut dysbiosis, oxidative stress, and systemic inflammation (Maloney and Lahiri 2016; Miyazaki et al. 2019; Agnihotri and Aruoma 2020). More research is needed to determine the extent to which diet can modify the neurotoxic outcomes of FC exposure.

### 6.2 Advances in ENS and GBA Experimental Models

The ENS engages in continuous bidirectional communication with the intestinal epithelium, muscle layers, and immune system, thereby regulating epithelial secretion and barrier function, motility, immune responses, and microbial composition (Montanari et al. 2023; Llorente 2024). Neurotransmitters such as ACh, 5‐HT, and VIP are central to these interactions. Understanding these complexes signaling networks is critical, as ENS dysfunction is implicated in various conditions, including GI disorders, enteric neuropathies, and NDs. Animal models, particularly rodents and pigs, remain the cornerstone of ENS research due to their ability to preserve the native multicellular architecture and dynamic interactions of the enteric microenvironment (Sharkey and Mawe 2023). Despite the recognized translational value of pig models for ENS research (Sharkey and Mawe 2023), there is still a major research gap, as studies limited their focus to local neurochemical or structural endpoints without evaluating functional consequences at the gut and brain level.

Moreover, current research in rodent models has focused on assessing toxicant sensitivity in knockout or transgenic animals carrying rare, highly penetrant PD‐causing mutations, for example, double transgenic mice expressing mutant α‐syn (Montanari et al. 2023). However, these models poorly represent idiopathic PD, which is increasingly understood as the result of complex gene–environment interactions. Common genetic variants, such as polymorphisms in membrane transporters, detoxification enzymes, or dopamine metabolism‐related genes, are thought to modulate individual susceptibility to environmental exposures. These models may not show severe brain damage, but they can help model prodromal PD, such as changes in behavior, gut function and GBA signaling. This approach would better reflect how the disease likely develops in most people and provide more relevant insight into how long‐term FCs exposures contribute to PD (Cannon and Greenamyre 2013).

Despite the irreplaceable value of in vivo models, species‐specific differences in enteric neurochemistry, neuronal subtypes, and gut physiology constrain their translational relevance to human health (Zhang et al. 2024). Moreover, according to the European Environmental Agency, over 80% of the chemicals used in the EU lack comprehensive hazard and exposure data, with over 3600 detected in human samples, and critical data gaps exist for many prioritized substances (Geueke et al. 2025). These limitations underscore the need for complementary experimental models and integrated testing approaches capable of efficiently screening the large number of potentially hazardous molecules. In this context, in vitro high‐throughput models emerge as essential tools to better and more efficiently assess human health risks.

Current in vitro research on the ENS still relies predominantly on primary cultures of enteric neurons and glia from rodents, owing to their accessibility and established protocols. However, these models suffer from the absence of standardized isolation and purification procedures, limited cell yield, and pronounced species‐specific differences that restrict their translational applicability. These limitations underscore the need to develop human‐based in vitro systems offering stable and scalable platforms to study ENS responses to environmental‐relevant stimuli. Recently, a human EGC line has been developed, providing a reproducible tool to study glial responses to environmental stressors (Zanoletti et al. 2023). A more advanced approach involves the use of human‐induced pluripotent stem cells (hiPSC) to derive enteric neural crest‐like progenitors that differentiate into mature enteric neurons, expressing nNOS, VIP, ChAT, calretinin, and capable of electrophysiological activity and functional integration in organotypic cocultures (W. Li et al. 2018). These neurons formed functional networks and demonstrated integration and survival upon transplantation into mouse gut tissue (W. Li et al. 2018). Additionally, efforts have been made to create more physiologically accurate models for studying neuroimmune, neuroepithelial, and neuromicrobiome interactions in disease states (Llorente 2024). Coculture systems mimicking the intestinal microenvironment, such as Caco‐2 epithelial cells with enteric glia or neurons, or myenteric and submucosal neurons for integration with intestinal organoids enable the investigation of epithelial–neural crosstalk under toxicological conditions (Miyazaki et al. 2019; Maruyama et al. 2023). Emerging bioengineered platforms, such as microfluidic gut–brain organ‐on‐chip systems, are incorporating ENS components, alongside intestinal epithelial and vascular elements (Zhang et al. 2024), and even microbial components to replicate gut–brain communication (Sedrani et al. 2023). For example, the neuroHuMiX model integrates hiPSC‐derived enteric neurons with epithelial and microbial components in separated compartments that allows to investigate how gut microbes influence enteric neurons through soluble signaling (Sedrani et al. 2023). Advances in microphysiological in vitro models have also contributed to the study of PD. Trapecar et al. (2021) developed a multiorgan system, 3XGLB, which integrates human gut, liver, and brain microphysiological units with circulating immune cells to investigate PD‐related mechanisms (Trapecar et al. 2021; Zhang et al. 2024), and even microbial components to replicate gut–brain communication (Sedrani et al. 2023). The model showed that gut‐derived SCFAs and immune signaling enhance brain cell maturation and promote PD‐related gene expression and inflammation, highlighting the role of gut–brain–immune interactions in PD pathogenesis.

## 7 Conclusions

Findings from both in vitro and in vivo studies reveal that exposure to FCs (pesticides, manganese, acrylamide, toxins, micro‐ and nanoplastics and bisphenols) induce marked neurochemical remodeling of the ENS and trigger enteric glial activation. In animal models, these changes are frequently accompanied by functional disturbances, including altered GI motility, low‐grade inflammation, and compromised epithelial barrier integrity, suggesting a coordinated disruption of gut homeostasis.

In addition to well‐established neurotoxicants such as rotenone and paraquat, emerging contaminants like PS‐MNPs have also been shown to induce or aggravate PD‐like pathology in the gut, via enteric gliosis and increase in α‐syn phosphorylation and aggregation, particularly when genetic predispositions exists. Moreover, studies using vagotomized animals report a significant reduction in α‐syn translocation and attenuated central neurotoxicity following FC exposure, supporting the hypothesis that the ENS mediates peripheral‐to‐central propagation of FC‐related neurotoxic signals. Collectively, these findings reinforce the mechanistic plausibility of ENS and vagal involvement in FC‐induced brain pathology, consistent with the body‐first model of neurodegeneration. Within the constraints of the current evidence, these findings are consistent with the notion that PD may, in part, be initiated at peripheral sites and modulated by environmental exposures.

Despite these advances, current evidence is largely derived from short‐term or high‐dose studies, limiting their relevance to real‐world human exposure scenarios. In this way, future research should prioritize long‐term, low‐dose exposure studies that better mimic realistic dietary intake patterns, to determine whether chronic FC exposure can trigger sustained ENS dysfunction and contribute to disease progression. Integrating high‐throughput derived experimental toxicity data, human epidemiological data, postmortem enteric tissue analysis, and microbiota profiling will be essential to validate these mechanisms and determine the broader public health implications.

## Author Contributions

**Helena Ramos**: methodology, investigation, writing – original draft, data curation, validation, visualization, formal analysis. **Ana Margarida Araújo**: conceptualization, investigation, methodology, data curation, visualization, validation, writing – review and editing. **Isabel M. P. L. V. O. Ferreira**: project administration, resources, supervision, conceptualization, funding acquisition, writing – review and editing. **Miguel A. Faria**: conceptualization, resources, supervision, writing – review and editing, methodology, investigation, validation, project administration.

## Conflicts of Interest

The authors declare no conflicts of interest.

## Supporting information




**Supporting Information**: crf370448‐sup‐0001‐SuppMat.docx
