# Supplementary material for: Enteric Nervous System Damage by Food Contaminants: A Pathway to Neurodegeneration?
Source: Compr Rev Food Sci Food Saf. 2026 Apr 9;25:e70448. doi: 10.1111/1541-4337.70448 (PMC13066547; doi:10.1111/1541-4337.70448)
Supplement: Supplementary file 1 — Supporting Information: crf370448‐sup‐0001‐SuppMat.docx [file CRF3-25-e70448-s001.docx]

**Supplementary Materials**

**Table S1.** Search queries applied in PubMed, Web of Science and Science Direct in 21 January 2026.

| Database | Search queries |
| --- | --- |
| **PubMed** | ("pesticidal"[All Fields] OR "pesticides"[Pharmacological Action] OR "pesticides"[Supplementary Concept] OR "pesticides"[All Fields] OR "pesticide"[All Fields] OR "pesticides"[MeSH Terms] OR ("toxine"[All Fields] OR "toxins, biological"[Supplementary Concept] OR "toxins, biological"[All Fields] OR "toxin"[All Fields] OR "toxins, biological"[MeSH Terms] OR ("toxins"[All Fields] AND "biological"[All Fields]) OR "biological toxins"[All Fields] OR "toxins"[All Fields]) OR ("metals, heavy"[Supplementary Concept] OR "metals, heavy"[All Fields] OR "heavy metals"[All Fields] OR "metals, heavy"[MeSH Terms] OR ("metals"[All Fields] AND "heavy"[All Fields]) OR ("heavy"[All Fields] AND "metals"[All Fields])) OR ("microplastics"[Supplementary Concept] OR "microplastics"[All Fields] OR "microplastic"[All Fields] OR "microplastics"[MeSH Terms]) OR ("nanoplastic"[All Fields] OR "nanoplastics"[All Fields]) OR ("acrylamide"[Supplementary Concept] OR "acrylamide"[All Fields] OR "acrylamide"[MeSH Terms] OR "acrylamides"[MeSH Terms] OR "acrylamides"[All Fields]) OR ("bisphenol a"[Supplementary Concept] OR "bisphenol a"[All Fields] OR "bisphenol"[All Fields] OR "bisphenolic"[All Fields] OR "bisphenols"[Supplementary Concept] OR "bisphenols"[All Fields] OR "bisphenols"[MeSH Terms]) OR ("phthalic acids"[Supplementary Concept] OR "phthalic acids"[All Fields] OR "phthalate"[All Fields] OR "phthalic acid"[Supplementary Concept] OR "phthalic acid"[All Fields] OR "phthalic acids"[MeSH Terms] OR ("phthalic"[All Fields] AND "acids"[All Fields]) OR "phthalates"[All Fields]) OR ("contaminant"[All Fields] OR "contaminants"[All Fields] OR "contaminate"[All Fields] OR "contaminated"[All Fields] OR "contaminates"[All Fields] OR "contaminating"[All Fields] OR "contamination"[All Fields] OR "contaminations"[All Fields] OR "contaminative"[All Fields] OR "contamined"[All Fields])) AND ("vagus nerve"[MeSH Terms] OR ("vagus"[All Fields] AND "nerve"[All Fields]) OR "vagus nerve"[All Fields] OR ("enteric nervous system"[MeSH Terms] OR ("enteric"[All Fields] AND "nervous"[All Fields] AND "system"[All Fields]) OR "enteric nervous system"[All Fields]) OR ("myenteric plexus"[MeSH Terms] OR ("myenteric"[All Fields] AND "plexus"[All Fields]) OR "myenteric plexus"[All Fields]) OR ("submucous plexus"[MeSH Terms] OR ("submucous"[All Fields] AND "plexus"[All Fields]) OR "submucous plexus"[All Fields])) |
| **Web of Science** | (pesticides OR toxins OR heavy metals OR microplastics OR nanoplastics OR acrylamide OR bisphenols OR phthalates OR contaminant) AND (vagus nerve OR enteric nervous system OR myenteric plexus OR submucous plexus) (All Fields) |
| **Science Direct** | Searches were separated due to the limitation of 8 boolean operators in one search query and duplicates were screened in Endnote. Search query was applied in Title, abstract or author-specified keywords section.  Search 1: (pesticides OR toxins OR heavy metals OR microplastics OR acrylamide OR bisphenols OR phthalates OR contaminant) AND (vagus nerve)  Search 2: (pesticides OR toxins OR heavy metals OR microplastics OR acrylamide OR bisphenols OR phthalates OR contaminant) AND (enteric nervous system)  Search 3: (pesticides OR toxins OR heavy metals OR microplastics OR acrylamide OR bisphenols OR phthalates OR contaminant) AND (myenteric plexus)  Search 4: (pesticides OR toxins OR heavy metals OR microplastics OR acrylamide OR bisphenols OR phthalates OR contaminant) AND (submucous plexus)  Search 5: (nanoplastics) AND (vagus nerve OR enteric nervous system OR myenteric plexus OR submucous plexus) |

**Table S2**. Quality assessment of the 13 *in vitro* and *ex vivo* included studies using the ToxRTool (Schneider et al. 2009). Red-shaded criteria indicate mandatory ToxRTool questions required for a study to be considered reliable.

| ***In Vitro/ Ex Vivo* Study** | **Test substance identification** | | | | **Test system characterization** | | | **Study design description** | | | | | | **Study results documentation** | | | **Plausibility of study design and data** | | **Klimlisch**  **Score** | **Risk of bias** |
| --- | --- | --- | --- | --- | --- | --- | --- | --- | --- | --- | --- | --- | --- | --- | --- | --- | --- | --- | --- | --- |
|  | **Q1** | **Q2** | **Q3** | **Q4** | **Q5** | **Q6** | **Q7** | **Q8** | **Q9** | **Q10** | **Q11** | **Q12** | **Q13** | **Q14** | **Q15** | **Q16** | **Q17** | **Q18** |  |  |
| Ghaisas, 2021 (Ghaisas et al. 2021) | 1 | 0 | 1 | 1 | 1 | 1 | 1 | 1 | 1 | 1 | 1 | 1 | 1 | 1 | 1 | 1 | 1 | 1 | 17 | Reliable without restrictions |
| Lourenssen, 2009 (Lourenssen, Miller, and Blennerhassett 2009) | 1 | 0 | 1 | 1 | 1 | 1 | 0 | 0 | 1 | 1 | 1 | 1 | 1 | 1 | 1 | 1 | 1 | 1 | 15 | Reliable without restrictions |
| Virga,  2018 (Virga, Capps, and Vohra 2018) | 1 | 0 | 1 | 1 | 1 | 1 | 1 | 0 | 1 | 1 | 1 | 1 | 0 | 1 | 1 | 1 | 1 | 1 | 15 | Reliable without restrictions |
| Brand, 2019  (Brand et al. 2019) | 1 | 0 | 1 | 0 | 1 | 1 | 1 | 0 | 1 | 1 | 1 | 1 | 1 | 1 | 1 | 1 | 1 | 1 | 15 | Reliable without restrictions |
| Liang, 2025  (Liang et al. 2025) | 1 | 1 | 1 | 1 | 1 | 1 | 0 | 0 | 1 | 1 | 1 | 1 | 1 | 1 | 1 | 1 | 1 | 1 | 16 | Reliable without restrictions |
| Guan, 2017  (Guan et al. 2017) | 1 | 0 | 0 | 1 | 1 | 1 | 1 | 0 | 1 | 1 | 1 | 1 | 1 | 1 | 1 | 1 | 1 | 1 | 15 | Reliable without restrictions |
| Miyazaki, 2013 (Miyazaki et al. 2019) | 1 | 0 | 1 | 1 | 1 | 1 | 1 | 1 | 1 | 1 | 1 | 1 | 1 | 1 | 1 | 1 | 1 | 1 | 17 | Reliable without restrictions |
| Reale, 2021  (Reale et al. 2021) | 1 | 0 | 1 | 1 | 1 | 1 | 1 | 1 | 1 | 1 | 1 | 1 | 1 | 1 | 1 | 1 | 1 | 1 | 17 | Reliable without restrictions |
| Diss, 2016  (Diss et al. 2016) | 1 | 0 | 0 | 1 | 1 | 1 | 1 | 0 | 1 | 1 | 1 | 1 | 1 | 1 | 1 | 1 | 1 | 1 | 15 | Reliable without restrictions |
| Arnhold, 2016 (Arnhold et al. 2016) | 1 | 0 | 1 | 1 | 1 | 1 | 1 | 1 | 1 | 1 | 1 | 1 | 0 | 1 | 1 | 1 | 1 | 1 | 16 | Reliable without restrictions |
| Pan-Montojo, 2012 (Pan-Montojo et al. 2012) | 1 | 0 | 1 | 1 | 1 | 1 | 1 | 1 | 1 | 1 | 1 | 1 | 1 | 1 | 1 | 1 | 1 | 1 | 17 | Reliable without restrictions |
| Sharrad, 2017 (Sharrad et al. 2017) | 1 | 0 | 0 | 1 | 1 | 1 | 1 | 1 | 1 | 1 | 1 | 1 | 1 | 1 | 1 | 1 | 1 | 1 | 16 | Reliable without restrictions |
| Dąbrowski, 2025  (Dabrowski et al. 2025) | 1 | 1 | 1 | 1 | 1 | 1 | 1 | 1 | 1 | 1 | 1 | 1 | 1 | 1 | 1 | 1 | 1 | 1 | 1 | Reliable without restrictions |

**Table S3**. Quality assessment of the *in vivo* included studies using the ToxRTool (Schneider et al. 2009). Red-shaded criteria indicate mandatory ToxRTool questions required for an *in vivo* study to be considered reliable.

| ***In Vivo* Study** | **Test substance identification** | | | | **Test system characterization** | | | | | **Study design description** | | | | | | | **Study results documentation** | | | **Plausibility of study design and data** | | **Klimlisch** | **Risk of bias** |
| --- | --- | --- | --- | --- | --- | --- | --- | --- | --- | --- | --- | --- | --- | --- | --- | --- | --- | --- | --- | --- | --- | --- | --- |
|  | **Q1** | **Q2** | **Q3** | **Q4** | **Q5** | **Q6** | **Q7** | **Q8** | **Q9** | **Q10** | **Q11** | **Q12** | **Q13** | **Q14** | **Q15** | **Q16** | **Q17** | **Q18** | **Q19** | **Q20** | **Q21** |  |  |
| Darwiche, 2017 (Darwiche et al. 2017) | 1 | 1 | 1 | 1 | 1 | 1 | 1 | 1 | 1 | 1 | 1 | 1 | 1 | 1 | 0 | 0 | 1 | 1 | 1 | 1 | 1 | 19 | Reliable without restrictions |
| Li, 2024 (Li et al. 2024) | 1 | 0 | 1 | 1 | 1 | 1 | 1 | 1 | 1 | 1 | 1 | 1 | 1 | 1 | 1 | 1 | 1 | 1 | 1 | 1 | 1 | 20 | Reliable without restrictions |
| Pupim, 2023 (Pupim et al. 2023) | 1 | 0 | 1 | 1 | 1 | 1 | 1 | 1 | 1 | 1 | 1 | 1 | 1 | 1 | 0 | 0 | 1 | 1 | 1 | 1 | 1 | 18 | Reliable without restrictions |
| Gaige, 2014 (Gaige et al. 2014) | 1 | 0 | 1 | 1 | 1 | 1 | 1 | 1 | 1 | 1 | 1 | 1 | 1 | 1 | 1 | 1 | 1 | 1 | 1 | 1 | 1 | 20 | Reliable without restrictions |
| Liang, 2025 (Liang et al. 2025) | 1 | 0 | 1 | 1 | 1 | 1 | 1 | 1 | 0 | 1 | 1 | 1 | 1 | 1 | 0 | 0 | 1 | 1 | 1 | 1 | 1 | 17 | Reliable with restrictions |
| Anadón, 2006 (Anadon et al. 2006) | 1 | 1 | 1 | 1 | 1 | 1 | 1 | 1 | 1 | 1 | 1 | 1 | 1 | 1 | 1 | 1 | 1 | 1 | 1 | 1 | 1 | 21 | Reliable without restrictions |
| Rissato, 2020 (Rissato et al. 2020) | 1 | 0 | 0 | 1 | 1 | 1 | 1 | 1 | 1 | 1 | 1 | 1 | 1 | 1 | 0 | 0 | 1 | 1 | 1 | 1 | 1 | 17 | Reliable with restrictions |
| Ghaisas, 2021 (Ghaisas et al. 2021) | 1 | 0 | 1 | 1 | 1 | 1 | 1 | 1 | 1 | 1 | 1 | 1 | 1 | 1 | 0 | 0 | 1 | 1 | 1 | 1 | 1 | 18 | Reliable without restrictions |
| Sousa, 2014 (Sousa et al. 2014) | 1 | 0 | 1 | 1 | 1 | 1 | 1 | 1 | 1 | 1 | 1 | 1 | 1 | 1 | 0 | 0 | 1 | 1 | 1 | 1 | 1 | 18 | Reliable without restrictions |
| Nanni, 2022 (Nanni et al. 2022) | 1 | 0 | 1 | 1 | 1 | 1 | 1 | 1 | 1 | 1 | 1 | 1 | 1 | 1 | 1 | 0 | 1 | 1 | 1 | 1 | 1 | 19 | Reliable without restrictions |
| Lee, 2022 (Lee et al. 2022) | 1 | 0 | 1 | 1 | 1 | 1 | 1 | 1 | 1 | 1 | 1 | 1 | 1 | 1 | 0 | 0 | 1 | 1 | 1 | 1 | 1 | 18 | Reliable without restrictions |
| Augustyniak, 2025 (Augustyniak et al. 2025) | 1 | 0 | 1 | 0 | 1 | 0 | 0 | 1 | 0 | 1 | 1 | 1 | 1 | 1 | 1 | 0 | 1 | 1 | 1 | 1 | 1 | 15 | Reliable with restrictions |
| Sousa, 2022 (Sousa et al. 2022) | 1 | 0 | 1 | 1 | 1 | 1 | 1 | 1 | 1 | 1 | 1 | 1 | 1 | 1 | 0 | 0 | 1 | 1 | 1 | 1 | 1 | 18 | Reliable without restrictions |
| Girardet, 2011 (Girardet et al. 2011) | 1 | 0 | 1 | 1 | 1 | 1 | 1 | 1 | 1 | 1 | 1 | 1 | 1 | 1 | 1 | 1 | 1 | 1 | 1 | 1 | 1 | 20 | Reliable without restrictions |
| Anselmi, 2018 (Anselmi et al. 2018) | 1 | 0 | 1 | 1 | 1 | 0 | 1 | 1 | 1 | 1 | 1 | 1 | 1 | 1 | 0 | 0 | 1 | 1 | 1 | 1 | 1 | 17 | Reliable with restrictions |
| Tasseli, 2013 (Tasselli et al. 2013) | 1 | 0 | 0 | 1 | 1 | 1 | 1 | 1 | 0 | 1 | 1 | 1 | 1 | 1 | 1 | 0 | 1 | 1 | 1 | 1 | 1 | 17 | Reliable with restrictions |
| Pan-Montojo, 2010 (Pan-Montojo et al. 2010) | 1 | 0 | 1 | 1 | 1 | 0 | 1 | 1 | 0 | 1 | 1 | 1 | 1 | 1 | 1 | 1 | 1 | 1 | 1 | 1 | 1 | 18 | Reliable without restrictions |
| Arnhold, 2016 (Arnhold et al. 2016) | 1 | 0 | 0 | 1 | 1 | 0 | 1 | 1 | 0 | 1 | 1 | 1 | 1 | 1 | 1 | 0 | 1 | 1 | 1 | 1 | 1 | 17 | Reliable with restrictions |
| Schaffermicht, 2021 (Sharrad et al. 2017) | 1 | 0 | 0 | 1 | 1 | 1 | 1 | 1 | 0 | 1 | 1 | 1 | 1 | 1 | 1 | 0 | 1 | 1 | 1 | 1 | 1 | 18 | Reliable without restrictions |
| Naudet, 2017 (Naudet et al. 2017) | 1 | 0 | 1 | 1 | 1 | 1 | 1 | 1 | 0 | 1 | 1 | 1 | 1 | 1 | 1 | 1 | 1 | 1 | 1 | 1 | 1 | 19 | Reliable without restrictions |
| Rudyk, 2020 (Rudyk et al. 2020) | 1 | 0 | 1 | 0 | 1 | 1 | 1 | 1 | 0 | 1 | 1 | 1 | 1 | 1 | 1 | 0 | 1 | 1 | 1 | 1 | 1 | 17 | Reliable with restrictions |
| Kras, 2022 (Kras et al. 2022) | 1 | 0 | 1 | 0 | 1 | 0 | 1 | 1 | 0 | 1 | 1 | 1 | 1 | 1 | 1 | 0 | 1 | 1 | 1 | 1 | 1 | 16 | Reliable with restrictions |
| BPs-exposed pigs(Szymanska, Calka, and Gonkowski 2018; Szymanska and Gonkowski 2018; Szymanska, Makowska, and Gonkowski 2018; Szymanska and Gonkowski 2019; Gonkowski et al. 2020; Makowska and Gonkowski 2020; Szymanska et al. 2020; Makowska et al. 2021; Makowska and Gonkowski 2022) | 1 | 0 | 1 | 1 | 1 | 1 | 1 | 1 | 0 | 1 | 1 | 1 | 1 | 1 | 1 | 0 | 1 | 1 | 1 | 1 | 1 | 18 | Reliable without restrictions |
| BPs-exposed mice (Makowska, Lepiarczyk, and Gonkowski 2022; Makowska, Calka, and Gonkowski 2023; Makowska, Fagundes, and Gonkowski 2023; Makowska and Gonkowski 2023, 2024) | 1 | 0 | 1 | 1 | 1 | 1 | 1 | 1 | 0 | 1 | 1 | 1 | 1 | 1 | 1 | 0 | 1 | 1 | 1 | 1 | 1 | 18 | Reliable without restrictions |
| BPs-exposed mice (Makowska et al. 2025) | 1 | 0 | 1 | 1 | 1 | 1 | 1 | 1 | 1 | 1 | 1 | 1 | 1 | 1 | 1 | 1 | 1 | 1 | 1 | 1 | 1 | 19 | Reliable without restrictions |
| PET-MPs exposed pigs qa(Galecka and Calka 2024a, 2024b; Galecka, Szyrynska, and Calka 2024) | 1 | 0 | 1 | 1 | 1 | 1 | 1 | 1 | 0 | 1 | 1 | 1 | 1 | 1 | 1 | 0 | 1 | 1 | 1 | 1 | 1 | 18 | Reliable without restrictions |
| ZEN/T2-exposed pigs (Makowska et al. 2017; Makowska, Obremski, and Gonkowski 2018; Rychlik et al. 2020) | 1 | 0 | 0 | 1 | 1 | 1 | 1 | 1 | 0 | 1 | 1 | 1 | 1 | 1 | 0 | 0 | 1 | 1 | 1 | 1 | 1 | 16 | Reliable with restrictions |
| ACR-exposed pigs (Palus, Bulc, and Calka 2018; Palus, Makowska, and Calka 2018; Palus and Calka 2019; Palus, Makowska, and Calka 2019; Palus et al. 2019; Palus, Bulc, and Calka 2020a, 2020b; Bulc, Calka, and Palus 2022; Karpiesiuk, Calka, and Palus 2023) | 1 | 1 | 1 | 1 | 1 | 1 | 1 | 1 | 0 | 1 | 1 | 1 | 1 | 1 | 0 | 0 | 1 | 1 | 1 | 1 | 1 | 18 | Reliable without restrictions |
| GLY-exposed pigs (Bulc, Calka, and Palus 2023; Palus et al. 2024; Palus, Karpiesiuk, and Jana 2025) | 1 | 1 | 1 | 1 | 1 | 1 | 1 | 1 | 0 | 1 | 1 | 1 | 1 | 1 | 0 | 0 | 1 | 1 | 1 | 1 | 1 | 18 | Reliable without restrictions |

**Table S4**. Summary of test compound characteristics, sources, and administration details across included studies.

| **Test Compound Reference** | **Source** | **Characteristics/Details** | **Purity** | **Administration Details** |
| --- | --- | --- | --- | --- |
| Manganese  (Ghaisas et al. 2021) | Sigma-Aldrich | MnCl₂·4H₂O | n.r. | Oral gavage in mice   - Diet with known low Mn concentration (75 mg/kg) - Dose/concentration measured as MnCl₂·4H₂O - Vehicle: water |
| Acrylamide  (Palus, Bulc, and Calka 2018; Palus, Makowska, and Calka 2018; Palus and Calka 2019; Palus, Makowska, and Calka 2019; Palus et al. 2019; Palus, Bulc, and Calka 2020a, 2020b; Bulc, Calka, and Palus 2022; Karpiesiuk, Calka, and Palus 2023) | Sigma-Aldrich | - | >99% | Oral administration in pig   - Gelatine capsules in morning feed |
| Acrylamide  (Lourenssen, Miller, and Blennerhassett 2009) | Sigma-Aldrich | - | n.r. | *In vitro* model   - Vehicle: unknown |
| PET-MPs  (Galecka and Calka 2024b, 2024a; Galecka, Szyrynska, and Calka 2024) | Good Fellow Cambridge Ltd (Huntingdon, UK)  Cat. no. ES306031/1; powder form | Shape: Heterogenous (spherical, fibrous, irregular)  Particle size: Various sizes (7.6 - 416.9 um; predominant 158.5 um)  Analysis: laser diffraction analysis, SEM analysis | n.a. | Oral administration in pig   - Gelatine capsules in morning feed |
| PS-NPs  (Liang et al. 2025) | MREDA (Beijing, China) | FITC or PE-labeled or nonfluorescent PS (50 mg/mL, 50 nm)  Shape: spherical  Particle size: 60 nm  Zeta-potential (mV) in ultrapure H₂O: -41.1± 1.5  Analysis: TEM, dynamic light scattering, FTIR, ^1^H-NMR | n.a. | Oral gavage/gastrointestinal injections in mice   - Vehicle: PBS |
| PS-NPs  (Li et al. 2024) | Shangai Huge Biotecnology Co., Ltd. (Shanghai, China) | Purchased as a dispersion (10% w/v)  Shape: spherical  Particle size: 100 nm  Zeta-potential (mV) in ultrapure H₂O: − 37.30 ± 1.72  Analysis: TEM, Raman spectroscopy, dynamic light scattering (to assess colloidal stability) | n.a. | Oral gavage in mice   - Vehicle: sterile Milli-Q water - Volume: 0.25 mL/animal - Concentration: 200 µg/mL |
| PS-NPs  (Augustyniak et al. 2025) | Lab261 (Palo Alto, CA, USA) | PS-NPs functionalized with an amine group and labeled with a fluorochrome (excitation/emission: 545/566 nm)  Shape: spherical  Particle size: 25 nm  Stable in physiological conditions with no agglomeration (no data referring to TEM or zeta-potential) | n.a. | Intragastric administration in rat   - Vehicle: 0.9% NaCl - Volume: 0.20 mL/animal |
| PS-MPs  (Lee et al. 2022) | Thermo Scientific | Ref. C37278 (Fluorescent red; 4% w/v; manufacturer website states product is carboxyl charge-stabilized hydrophobic polystyrene microspheres, normally used for flow cytometry)  Shape: spherical  Particle size: 2 µm  Zeta-potential (mV) in ultrapure water: − 32 ± 0.3  Analysis: Raman spectroscopy only to detect PS-MPs inside the tissue | n.a. | Oral gavage in mice   - Vehicle: water |
| BPA  (Szymanska, Calka, and Gonkowski 2018; Szymanska and Gonkowski 2018; Szymanska, Makowska, and Gonkowski 2018; Szymanska and Gonkowski 2019; Gonkowski et al. 2020; Makowska and Gonkowski 2020; Szymanska et al. 2020; Makowska et al. 2021; Makowska and Gonkowski 2022) | Sigma-Aldrich | Ref: 239658 | >99% | Oral administration in pig   - Gelatine capsules in morning feed |
| BPA/BPS  (Makowska, Lepiarczyk, and Gonkowski 2022; Makowska, Calka, and Gonkowski 2023; Makowska, Fagundes, and Gonkowski 2023; Makowska and Gonkowski 2023, 2024) | Sigma-Aldrich | BPA Ref: 239658  BPS Ref: 43024 | n.r. | Oral gavage in mice   - Water |
| BPA/BPS  (Makowska et al. 2025) | Sigma-Aldrich | - | n.r. | Oral gavage in mice   - Vehicle: 0.5% ethanol in drinking water - Concentration: 25 mg/mL |
| Rotenone  (Arnhold et al. 2016) | n.r. | - | n.r. | Oral gavage in mice   - Vehicle: 2% methylcellulose and 1.25% chloroform - Volume: 0.01 mL/g   *In vitro* model   - Vehicle: 0.05% ethanol |
| Rotenone  (Guan et al. 2017) | n.r. | - | n.r. | *In vitro* model   - Vehicle: unknown |
| Rotenone  (Miyazaki et al. 2019) | Sigma-Aldrich | - | n.r. | *In vitro* model   - Vehicle: DMSO |
| Rotenone  (Pan-Montojo et al. 2010) | Sigma-Aldrich | - | n.r. | Oral gavage in mice   - Vehicle: 4% carboxymethylcellulose and 1.25% chloroform - Volume: 0.01 mL/g - Concentration: 0.625 mg/mL |
| Rotenone  (Schaffernicht et al. 2021) | n.r. | - | n.r. | Oral gavage in mice   - Vehicle: 2% methylcellulose and 1.25% chloroform - Volume: 0.01 mL/g |
| Rotenone  (Sharrad et al. 2017) | n.r. | - | n.r. | *In vitro* model   - Vehicle: 0.001%, 0.01% DMSO |
| Rotenone  (Tasselli et al. 2013) | Sigma-Aldrich | - | n.r. | Oral gavage in mice   - Vehicle: 4% chloroform and 0.5% carboxymethyl cellulose sodium salt |
| Rotenone  (Virga, Capps, and Vohra 2018) | n.r. | - | n.r. | *In vitro* model   - Vehicle: ethanol |
| Rotenone  (Ahn et al. 2020) | ULTRA Scientific (PST-890) | - | n.r. | Oral gavage in mice   - Vehicle: 1% methylcellulose and 1.25% chloroform - Volume: 0.1 mL/25 g - Concentration: 0.625 mg/mL |
| Rotenone  (McQuade et al. 2021) | n.r. | - | n.r. | Oral gavage in mice   - Vehicle: 1.25% chloroform and 1% carboxymethyl cellulose sodium salt - Volume: 0.05 mL/10 g |
| Rotenone  (Pan-Montojo et al. 2012) | Sigma-Aldrich | - | n.r. | Oral gavage in mice   - Vehicle: 4% methylcellulose and 1.25% chloroform - Volume: 0.2 mL/25 g - Concentration: 0.625 mg/mL |
| λ-cyhalothrin  (Anadon et al. 2006) | Zeneca Agrochemicals | Compound: λ-cyhalothrin (mixture 1:1 of S and R isomers)  CAS: 91465-08-6 | 98.8% | Oral gavage in rat   - Vehicle: corn oil - Volume: 0.5 mL/animal |
| Paraquat  (Anselmi et al. 2018) | n.r. | - | n.r. | Oral gavage in rat   - Vehicle: 1% sucrose |
| Chlorpyrifos  (Darwiche et al. 2017) | LGC Standards | - | 99.8% | Oral gavage in rat  Vehicle: rapeseed oil |
| Paraquat  (Diss et al. 2016) | n.r. |  | n.r. | *In vitro* model   - Vehicle: unknown |
| 2,4-D  (Nanni et al. 2022) | n.r. | - | n.r. | Oral gavage in rat   - Vehicle: distilled water - Volume: 1 mL/rat |
| Paraquat  (Naudet et al. 2017) | Sigma-Aldrich | Ref: 856177  Compound: methyl viologen dichloride hydrate | n.r. | Oral administration in mice   - Vehicle: drinking water - Volume: 1.5 mL/10 g - Concentration: 50 µg/mL |
| Glyphosate  (Bulc, Calka, and Palus 2023; Palus et al. 2024; Palus, Karpiesiuk, and Jana 2025) | Sigma-Aldrich | CAS: 1071-83-6 | 99.5% | Oral administration in pig:   - Gelatine capsules in morning feed |
| Malathion  (Pupim et al. 2023) | Dominus Quimica | Compound: diethyldimethoxythiophosphorylthio  CAS: 121-75-5 | n.r. | Oral gavage in rat   - Vehicle: 0.9% saline solution |
| T-2 toxin  (Gaige et al. 2014) | Sigma-Aldrich | Ref: T4887 | n.r. | Oral gavage in mice   - Vehicle: 0.2-2% DMSO in distilled water - Volume: 5 µL/g |
| Deoxynivalenol  (Girardet et al. 2011) | Sigma-Aldrich | Ref: D-0156 | n.r. | Oral gavage in mice   - Vehicle: distilled water - Volume: 100 µL/10 g |
| T-2 toxin  (Makowska et al. 2017; Makowska, Obremski, and Gonkowski 2018; Rychlik et al. 2020) | n.r. | - | n.r. | Oral administration in pig   - Gelatine capsules in morning feed |
| ZEN  (Makowska et al. 2017) | n.r. | - | n.r. | Oral administration in pig   - Gelatine capsules in morning feed |
| Pectenotoxin-2  (Reale et al. 2021) | National Research Council Canada | - | n.r. | *In vitro* model   - Vehicle: methanol |
| Okadaic Acid  (Reale et al. 2021) | National Research Council Canada | - | n.r. | *In vitro* model   - Vehicle: methanol |
| Aflatoxin B1  (Dąbrowski et al., 2025) | Sigma Aldrich, Lyon, France |  | >98% | *In vitro* model   - Vehicle: DMSO |
| Apicidin  (Dąbrowski et al., 2025) | Sigma Aldrich |  | >98% | In vitro exposure; vehicle: DMSO |
| Aurofusarin  (Dąbrowski et al., 2025) | Santa Cruz Biotechnology, Dallas, TX, USA |  | >97% | In vitro exposure; vehicle: DMSO |
| Beauvericin  (Dąbrowski et al., 2025) | Sigma Aldrich |  | >97% | In vitro exposure; vehicle: ethanol |
| Brevianamide-F  (Dąbrowski et al., 2025) | BioAustralis, Smithfield, Australia |  | >95% | In vitro exposure; vehicle: ethanol |
| Cyclo-(L-Pro-L-Tyr)  (Dąbrowski et al., 2025) | BioAustralis |  | >98% | In vitro exposure; vehicle: ethanol |
| Deoxynivalenol (Dąbrowski et al., 2025) | Sigma Aldrich |  | >98% | In vitro exposure; vehicle: ethanol |
| Emodin (Dąbrowski et al., 2025) | Sigma Aldrich |  | >90% | In vitro exposure; vehicle: DMSO |
| Enniatins (Dąbrowski et al., 2025) | Sigma Aldrich |  | >99% | In vitro exposure; vehicle: ethanol |
| Fumonisin B1 (Dąbrowski et al., 2025) | Sigma Aldrich |  | >98% | In vitro exposure; vehicle: DMSO |
| Moniliformin (Dąbrowski et al., 2025) | Sigma Aldrich |  | >95% | In vitro exposure; vehicle: ethanol |
| Ochratoxin A (Dąbrowski et al., 2025) | Sigma Aldrich |  | >95% | In vitro exposure; vehicle: ethanol |
| Patulin (Dąbrowski et al., 2025) | Sigma Aldrich |  | >98% | In vitro exposure; vehicle: ethanol |
| Tryptophol (Dąbrowski et al., 2025) | Sigma Aldrich |  | >97% | In vitro exposure; vehicle: DMSO |
| Zearalenone (Dąbrowski et al., 2025) | Sigma Aldrich |  | >98% | In vitro exposure; vehicle: DMSO |
| Deoxynivalenol  (Rissato et al. 2020) | n.r. | - | n.r. | Oral administration in rat (food)   - DON in chow with known composition - DON was not quantified in control diet |
| Patulin extract  (Brand et al. 2019) | Fungal extract | Extracts isolated from Penicillium coprobium cultures from strain IBWF D03003  Extracts were generated from the mycelium after freeze-drying by extraction with acetone/MeOH and from the culture filtrate by direct extraction with ethyl acetate  Chromatographic analysis of patulin by HPLC-MS | 68% patulin | *In vitro* model   - Vehicle: DMSO |
| FB1/FB2 extract  (Kras et al. 2022) | Fungal extract | Extracts from culture of F. verticillioides in tryptone glucose yeast broth medium  Extraction from dried seeds with ethyl alcohol  HPLC analysis: FB1:FB2 ratio was 3:1 (73% to 27%) | n.r. | Intragastric administration in rat   - Vehicle: 0.9% saline solution |
| FB1/FB2 extract  (Rudyk et al. 2020) | Fungal extract | Extracts from culture of F. verticillioides in tryptone glucose yeast broth medium  Extracted with ethanol  HPLC analysis: contaminated corn contained 75% of FB1 and 25% of FB2 (182.0 and 59.5 µg/kg, respectively) | n.r. | Intragastric administration in rat   - Vehicle: 0.9% saline solution |
| FB1/FB2 extract  (Sousa et al. 2014) | Fungal extract | Sterilized culture medium of the fungus F. verticillioides, strain MRC 826 added to base diet  Samples of the diet (with/out the culture medium were quantified for FB1 and FB2) | n.r. | Oral administration in rat (food)   - Basal diet with known composition and 0.159 mg FB1/kg diet & FB2 n.d.; - F1 diet (0.996 mg FBs/kg) and F3 diet (2.819 mg/kg) at ~75% FB1/ 25% FB2 |
| FB1/FB2 extract  (Sousa et al. 2022) | Fungal extract | Sterilized culture medium of the fungus F. verticillioides, strain MRC 826 added to base diet  Samples of the diet (with/out the culture medium were quantified for FB1 and FB2) | n.r. | Oral administration in rat (food)   - Basal diet with known composition without FBs (confirmed by HPLC) - F1 diet (1.129 mg FBs/kg) and F3 diet (3.850 mg FBs/kg) at ~80%FB1, 20%FB2 |

**Table S5**. Overview of experimental endpoints assessed in studies focused on the ENS.

| Test Compound/References | Region of the gut analysed | ENS-related endpoints | Intestinal-related and other endpoints |
| --- | --- | --- | --- |
| Acrylamide  (Palus, Bulc, and Calka 2018; Palus, Makowska, and Calka 2018; Palus and Calka 2019; Palus, Makowska, and Calka 2019; Palus et al. 2019; Palus, Bulc, and Calka 2020a, 2020b; Bulc, Calka, and Palus 2022; Karpiesiuk, Calka, and Palus 2023) | Stomach, jejunum, duodenum, ileum | - Co-localization of neuronal markers - Double immunofluorescent staining - Quantification of nerve structures | - Histopathological assessment - ELISA: IL-1β, TNFα, and IL-6 |
| Bisphenol A  (Szymanska, Calka, and Gonkowski 2018; Szymanska and Gonkowski 2018; Szymanska, Makowska, and Gonkowski 2018; Szymanska and Gonkowski 2019; Gonkowski et al. 2020; Makowska and Gonkowski 2020; Szymanska et al. 2020; Makowska et al. 2021; Makowska and Gonkowski 2022) | Stomach, jejunum, duodenum, ileum, large intestine | - Co-localization of neuronal markers - Double immunofluorescent staining - Quantification of nerve structures | - Histopathological assessment - ELISA: IL-1β, TNFα, and IL-6 |
| Bisphenol A  Bisphenol S  (Makowska, Lepiarczyk, and Gonkowski 2022; Makowska, Calka, and Gonkowski 2023; Makowska, Fagundes, and Gonkowski 2023; Makowska and Gonkowski 2023, 2024) | Stomach, jejunum, colon | - Double immunofluorescent staining - Quantification of nerve structures | - Histopathological assessment |
| Bisphenol A  (Makowska et al. 2025) | Colon | - Immunofluorescent staining (VAchT, Ano-1, S100β) |  |
| PET-MPs  (Galecka and Calka 2024b, 2024a; Galecka, Szyrynska, and Calka 2024) | Duodenum, jejunum, ileum | - Double immunofluorescent staining | - Histopathological assessment - ELISA: IL-1β, TNFα, and IL-6 |
| T-2 toxin  (Makowska et al. 2017; Makowska, Obremski, and Gonkowski 2018; Rychlik et al. 2020) | Stomach, duodenum, colon | - Co-localization of neuronal markers - Double immunofluorescent staining |  |
| Zearalenone  (Makowska et al. 2017) | Colon | - Co-localization of neuronal markers - Double immunofluorescent staining |  |
| PS-NPs  (Augustyniak et al. 2025) | Jejunum | - Gene expression (fabp2, chga, lgr5, lyz1, muc2, muc1, map2, s100b, cspg4, ocln, cldn1, cldn2, cdh1, casp3, Il1b, tnf, Il23a, sod1, sod2, cat, gpx1, and gpx4) - Protein expression profiling (CAT, IL-1β, SOD1, SOD2, TNF-α) - Oxidative stress markers: MDA, total oxidant capacity, GSH, CAT and SOD activity - PS-NPs visualization/detection (confocal microscopy/TEM) - Untargeted Metabolomics (NMR spectroscopy) |  |
| Manganese  (Ghaisas et al. 2021) | Colon | - Gene expression: tnf, inos - Protein expression profiling: GFAP, iNOS, ferroportin, DMT-1, DJ-1 | - Transit time; bead latency; fecal pellet output - Gut microbiota (taxonomic abundances, fecal metabolite analysis) - ICP analysis for metal quantification |
| Rotenone  (Arnhold et al. 2016) | Colon | - Immunohistochemistry: α-syn; TH | - 1h stool collection test |
| Chlorpyrifos  (Darwiche et al. 2017) | Ileum | - Acetylcholinesterase activity - Gene and protein expression: iNOS | - 1h intestinal transit - Measurement of contraction of isolated rat ileum |
| Rotenone  (McQuade et al. 2021) | Colon | - Double immunofluorescent staining | - Fecal pellet output - Bead expulsion test |
| 2,4-dichlorophenoxyacetic acid  (Nanni et al. 2022) | Colon | - Quantification of enteric neurons - Morphometry of the neuronal cell body area |  |
| Glyphosate  (Bulc, Calka, and Palus 2023; Palus et al. 2024; Palus, Karpiesiuk, and Jana 2025) | Jejunum, duodenum, ileum, colon | - Double immunofluorescent staining - Gene expression: galr1, galr2, galr3, sod1, sod2 |  |
| Malathion  (Pupim et al. 2023) | Colon | - Double immunofluorescent staining - Butyrylcholinesterase enzyme activity | - Fecal pellet output |
| Rotenone  (Schaffernicht et al. 2021) | Jejunum, duodenum, ileum, colon | - WB: TH, ChAT, Muscarinic Acetylcholine Receptor, Dopamine receptor D2, PGP9.5 | - PET/MR imaging - Contractility Studies |
| Deoxynivalenol  (Rissato et al. 2020) | Jejunum | - Morphometry of the neuronal cell body area - Double immunofluorescent staining - NADPH-d histochemistry | - Enzymatic activity (SOD, GST, LOOH), GSH levels, LOOH levels |
| FB1/FB2 mixture  (Rudyk et al. 2020) | Duodenum, jejunum | - Morphometry of the neuronal cell body area - Immunohistochemistry to localize submucous and MP | - Serum Biochemical Analysis (AST, ALT, GGT) |
| FB1/FB2 mixture  (Sousa et al. 2014) | Jejunum | - Quantification of nerve structures - Morphometry of the neuronal cell body area - Double immunofluorescent staining |  |

**Table S6.** Overview of experimental endpoints assessed in studies that evaluated ENS–CNS communication.

| Test Compound/ Reference | Behavioural/Motor Tests | CNS-related endpoints | ENS-related endpoints | Intestinal Endpoints | Other Endpoints |
| --- | --- | --- | --- | --- | --- |
| PS-MPs  (Lee et al. 2022) | - Morris water maze - Open-field test - Fear conditioning - Elevated plus maze - Novel object recognition - Three-chamber sociability test | - BBB permeability - RNA-seq/qPCR (Aif1, IL-1β, TNF-α) - Synaptosomal plasma membrane - Immunofluorescence for visualization of different proteins | n.a. | n.a. | - Raman spectroscopy (detection of FCs in organism) |
| Rotenone  (Tasselli et al. 2013) | - Rotarod Test | - Immunohistochemical detection and counting of dopaminergic neurons (TH^+^) in SN | - Immunohistochemical analysis of the myenteric plexus (ChAT, nNOS, α-syn) | - Gastrointestinal motility (transit time, gastric emptying, fecal pellet output, bead latency) | - Body mass - Food intake |
| Cyhalothrin  (Anadon et al. 2006) | n.a. | n.a. | n.a. | n.a. | - Oral toxicokinetic studies: plasma and tissue levels |
| Rotenone  (Pan-Montojo et al. 2010) | n.a. | - Complex I activity - TH immunohistochemistry in SNpc - Co-immunostaining against α-syn and ChAT in spinal cord sections showing the intermediolateral nucleus neurons - DAB-staining against α-syn using synuclein-1 antibody in the dorsal horn of the spinal cord double-immunofluorescence staining against α-syn and ChAT on DMV sections | - βIII-tubulin, α-syn and DAPI staining in duodenum and ileum sections - Immunofluorescence staining using α-syn, thioflavine S and DAPI | n.a. | - Detection of ROT in plasma and CNS levels |
| Rotenone  (Ahn et al. 2020) | - Morris water maze - Novel object recognition - Tail suspension test - Grid performance test - Rotarod test | n.a. | n.a. | - Gastrointestinal motility (stool water content, transit time) | - Detection of ROT in colon and brain |
| Paraquat  (Anselmi et al. 2018) | - Vibrissae test - Stepping test | - Immunohistochemistry (TH) in SNpc - Co-localization of ChAT- or TH-immunoreactivity and 129Ser α-synuclein-immunoreactivity if DMV, A2, area, SNpc | - Immunohistochemistry (129Ser α-synuclein) | - Gastric tone and motility recordings | n.a. |
| T2 toxin  (Gaige et al. 2014) | n.a. | - c-FOS immunohistochemistry and quantification - Double immunofluorescent labelling c-Fos/NUCB2/nesfatin-1d - qPCR of AgRP, CART, MC4-R, NPY, POMC, IL-1β, IL-6, TNF-α, COX-2, mPGES-1, Iba-1, Vimentin, CAT, UCP-2, SOD-2, Nrf2, GAPDH | n.a. | n.a. | - Body temperature and locomotor activity - Indirect calorimetry - Glyceamia measurements - IL-1β plasmatic levels |
| Deoxynivalenol  (Girardet et al. 2011) | n.a. | - c-FOS immunohistochemistry and quantification - Double immunofluorescent labelling c-Fos/NUCB2/nesfatin-1d - qPCR of AgRP, CART, MC4-R, NPY, POMC | n.a. | n.a. | - Food intake |
| PS-NPs  (Li et al. 2024) | - Open-field test - Elevated plus maze - Y maze - Forelimb grip strength test - Cliff aversion test - Negative geotaxis test | - Immunohistochemistry (inflammation markers) - Targeted neurotransmitter metabolomics; KEGG analysis | - RNAseq (neurotransmitter-related markers) | - Hematoxylin and eosin staining, alcian blue-periodic acid schiff (AB-PAS) staining | - Gut microbiota analysis |
| PS-NPs  (Liang et al. 2025) | - Open-field test - Elevated plus maze - Rotarod test - Morris water maze - Strength grip test | - TH^+^ neurons in SN | n.a. | n.a. | - *In vitro* fluorescence imaging of test compounds - Immunofluorescence staining intestinal distribution |
| Paraquat  (Naudet et al. 2017) | - Locomotion test | n.a. | - Fluorescence staining of mucins in the colonic tissue (MUC1, MUC2, LPS, OCLDN, ZO-1) | n.a. | n.a. |
| Rotenone  (Pan-Montojo et al. 2012) | - Rotarod test | - In DMV: α-syn immunofluorescence intensity, ChAT^+^ neuronal counts, and stereological analysis of DMV neurons - In SN: TH^+^ dopaminergic neuronal counts, α-syn immunostaining, and stereological analysis for neuronal loss - In IML: α-syn immunoreactivity relative to adjacent areas to evaluate spread from ENS to CNS | - TUNEL staining; immunofluorescence staining | n.a. | n.a. |

**Notes:** *α-syn*, α-synuclein; *AChE*, acetylcholinesterase; *AgRP*, agouti-related peptide; *BBB*, blood–brain barrier; *BuChE*, butyrylcholinesterase; *CAT*, catalase; *ChAT*, choline acetyltransferase; *CNS*, central nervous system; *COX-2*, cyclooxygenase-2; *DAB*, 3,3′-diaminobenzidine; *DMV*, dorsal motor nucleus of the vagus; *ENS*, enteric nervous system; *FCs*, food contaminants; *GAPDH*, glyceraldehyde-3-phosphate dehydrogenase; *IL*, interleukin; *IL-1β*, interleukin-1 beta; *IL-6*, interleukin-6; *IML*, intermediolateral nucleus; *KEGG*, Kyoto Encyclopedia of Genes and Genomes; *LPS*, lipopolysaccharide; *MC4-R*, melanocortin 4 receptor; *MP*, myenteric plexus; *mPGES-1*, microsomal prostaglandin E synthase-1; *MUC*, mucin; *n.a.*, not applicable; *nNOS*, neuronal nitric oxide synthase; *NPY*, neuropeptide Y; *Nrf2*, nuclear factor erythroid 2-related factor 2; *OCLDN*, occludin; *POMC*, pro-opiomelanocortin; *PS-MPs*, polystyrene microplastics; *PS-NPs*, polystyrene nanoparticles; *qPCR*, quantitative polymerase chain reaction; *ROT*, rotenone; *SN*, substantia nigra; *SNpc*, substantia nigra pars compacta; *SOD-2*, superoxide dismutase-2; *TH*, tyrosine hydroxylase; *TUNEL*, terminal deoxynucleotidyl transferase dUTP nick-end labeling; *ZO-1*, zonula occludens-1.

**
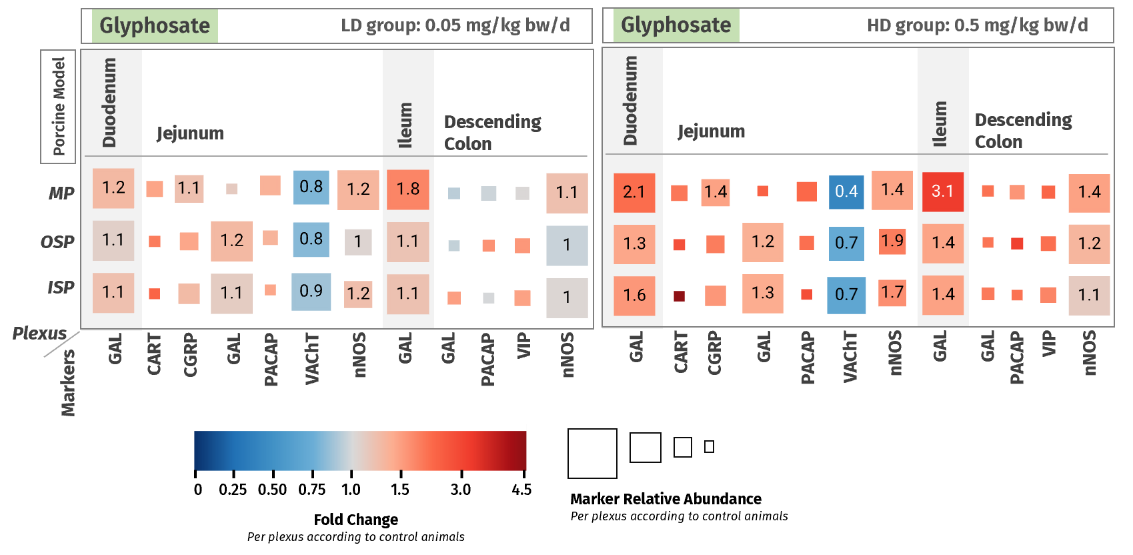
**

**Figure S1.** Square heatmap matrix showing the effect of glyphosate exposure on the immunoreactivity of intestinal neuronal subpopulations in a porcine model following 28-day exposure to a low-dose (LD, 0.05 mg GLY/kg bw/day) and high-dose (HD, 0.5 mg GLY/kg bw/day) group (Bulc, Calka, and Palus 2023; Palus et al. 2024). The heatmap displays fold-change values in neuronal marker immunoreactivity relative to untreated control animals. Color hue represents the direction and magnitude of change (red indicates upregulation, blue indicates downregulation), while the size of each square qualitatively reflects the relative abundance of the corresponding neuronal subpopulation in the control group within the analyzed gut region or plexus. Fold-change values are annotated within each square. Abbreviations: *MP*, myenteric plexus; *OSP*, outer submucosal plexus; *ISP*, inner submucosal plexus; *CART*, cocaine- and amphetamine-regulated transcript; *CGRP*, calcitonin gene-related peptide; *GAL*, galanin; *PACAP*, pituitary adenylate cyclase-activating polypeptide; *VAChT*, vesicular acetylcholine transporter; *VIP*, vasoactive intestinal peptide; *nNOS*, neuronal nitric oxide synthase.


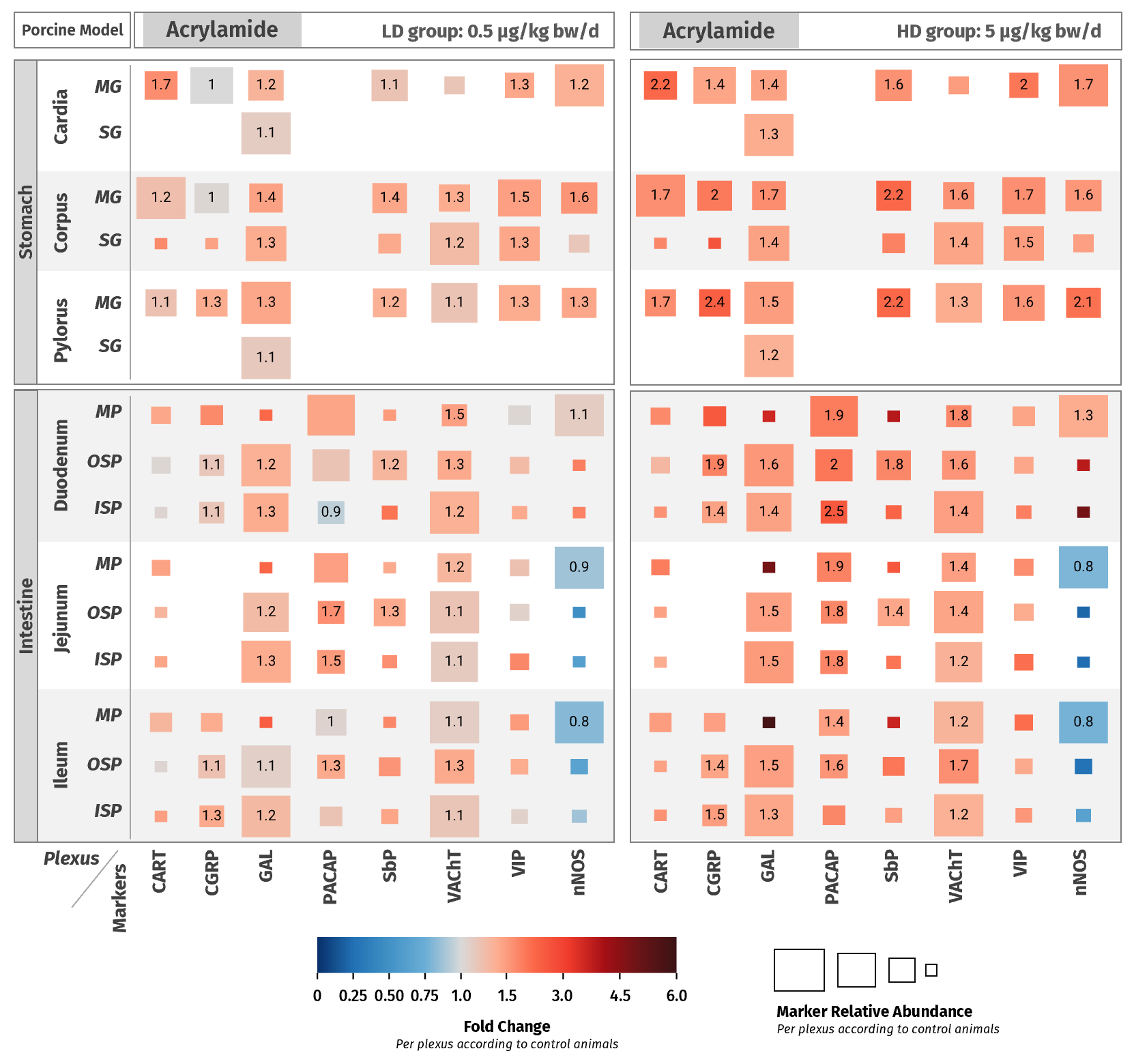


**Figure S2.** Square heatmap matrix showing the effect of acrylamide exposure on the immunoreactivity of intestinal neuronal subpopulations in a porcine model following 28-day exposure to a low-dose low dose (LD, 0.5 µg ACR/kg bw/day) and high dose (HD, 5 µg ACR/kg bw/day). The heatmap displays fold-change values in neuronal marker immunoreactivity relative to untreated control animals. Color hue represents the direction and magnitude of change (red indicates upregulation, blue indicates downregulation), while the size of each square qualitatively reflects the relative abundance of the corresponding neuronal subpopulation in the control group within the analyzed gut region or plexus. Fold-change values are annotated within each square. Abbreviations: *MG*, myenteric ganglia; *SG*, submucosal ganglia; *MP*, myenteric plexus; *OSP*, outer submucosal plexus; *ISP*, inner submucosal plexus; *CART*, cocaine- and amphetamine-regulated transcript; *CGRP*, calcitonin gene-related peptide; *GAL*, galanin; *PACAP*, pituitary adenylate cyclase-activating polypeptide; *SbP*, substance P; *VAChT*, vesicular acetylcholine transporter; *VIP*, vasoactive intestinal peptide; *nNOS*, neuronal nitric oxide synthase.


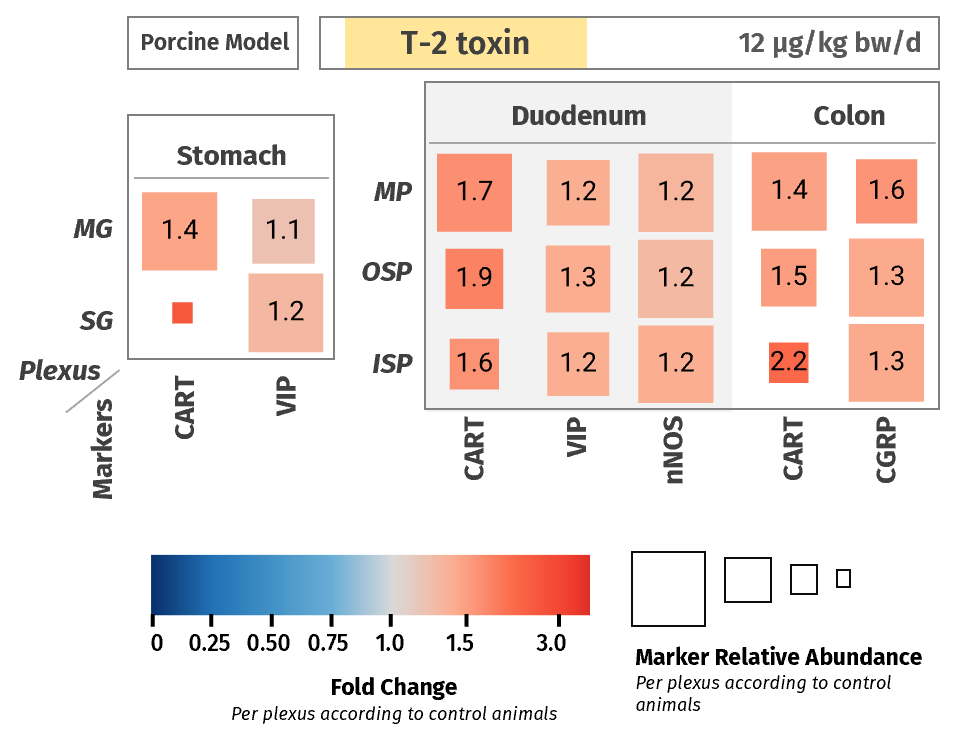


**Figure S3.** Square heatmap matrix showing the effect of T-2 exposure (12 µg/kg bw/day) on the immunoreactivity of intestinal neuronal subpopulations in a porcine model following 42-day exposure (Makowska et al. 2017; Makowska, Obremski, and Gonkowski 2018; Rychlik et al. 2020). The heatmap displays fold-change values in neuronal marker immunoreactivity relative to untreated control animals. Color hue represents the direction and magnitude of change (red indicates upregulation, blue indicates downregulation), while the size of each square qualitatively reflects the relative abundance of the corresponding neuronal subpopulation in the control group within the analyzed gut region or plexus. Fold-change values are annotated within each square. Abbreviations: *MG*, myenteric ganglia; *SG*, submucosal ganglia; *MP*, myenteric plexus; *OSP*, outer submucosal plexus; *ISP*, inner submucosal plexus; *CART*, cocaine- and amphetamine-regulated transcript; *CGRP*, calcitonin gene-related peptide; *VIP*, vasoactive intestinal peptide; *nNOS*, neuronal nitric oxide synthase.


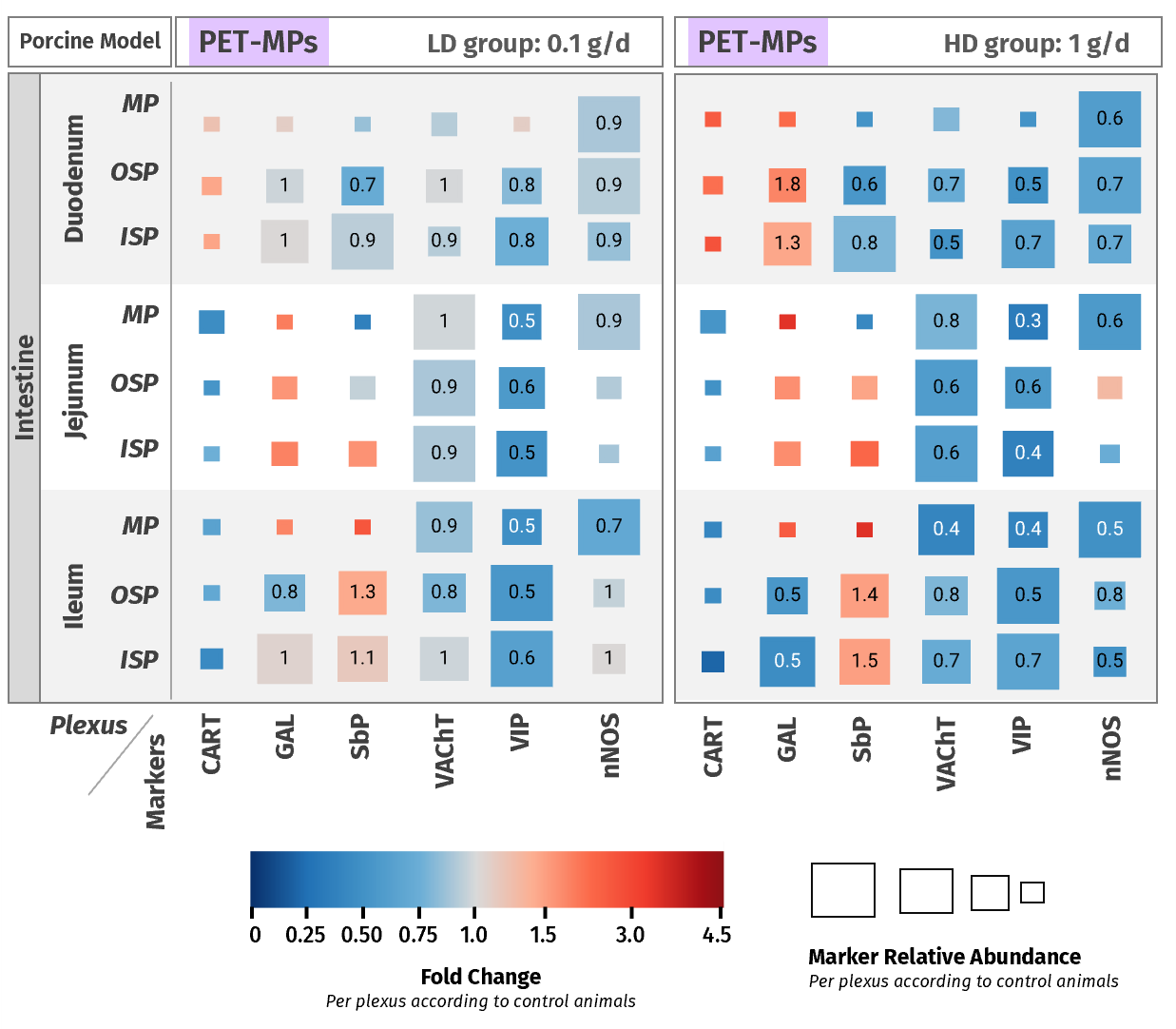


**Figure S4.** Square heatmap matrix showing the effect of PET-MPs exposure on the immunoreactivity of intestinal neuronal subpopulations in a porcine model following 28-day exposure to a low-dose low dose (LD, 0.1 g/day) and high dose (HD, 1 g/day) (Galecka and Calka 2024a, 2024b; Galecka, Szyrynska, and Calka 2024). The heatmap displays fold-change values in neuronal marker immunoreactivity relative to untreated control animals. Color hue represents the direction and magnitude of change (red indicates upregulation, blue indicates downregulation), while the size of each square qualitatively reflects the relative abundance of the corresponding neuronal subpopulation in the control group within the analyzed gut region or plexus. Fold-change values are annotated within each square. Abbreviations: *MP*, myenteric plexus; *OSP*, outer submucosal plexus; *ISP*, inner submucosal plexus; *CART*, cocaine- and amphetamine-regulated transcript; *CGRP*, calcitonin gene-related peptide; *GAL*, galanin; *SbP*, substance P; *VAChT*, vesicular acetylcholine transporter; *VIP*, vasoactive intestinal peptide; *nNOS*, neuronal nitric oxide synthase.


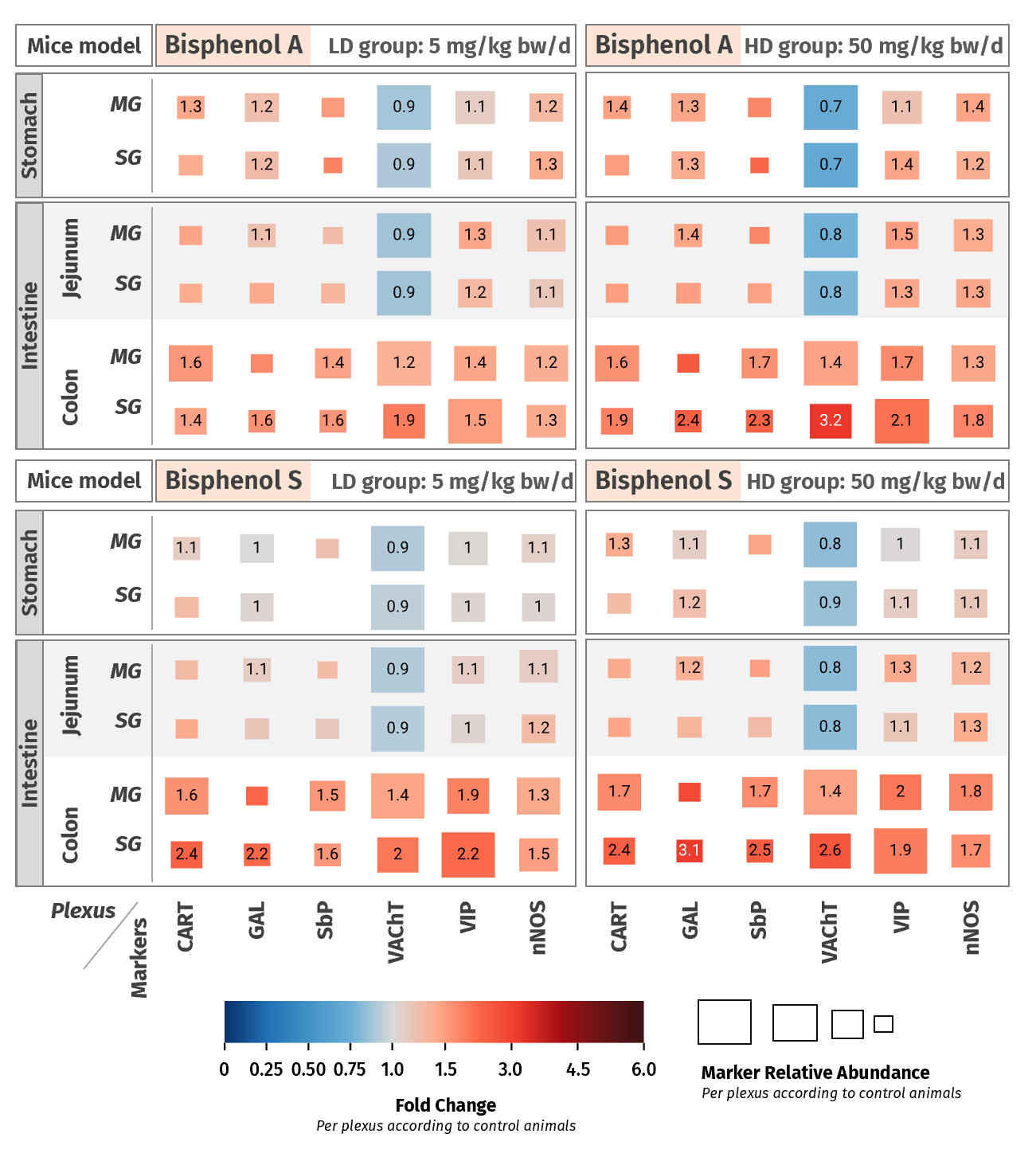


**Figure S5.** Square heatmap matrix showing the effect of bisphenol A (first panel) and bisphenol S (second panel) exposure on the immunoreactivity of intestinal neuronal subpopulations in CD1 mice following a 3-months exposure to a low-dose (LD, 5 mg/kg bw/day) and high-dose (HD, 50 mg/kg bw/day). The heatmap displays fold-change values in neuronal marker immunoreactivity relative to untreated control animals. Color hue represents the direction and magnitude of change (red indicates upregulation, blue indicates downregulation), while the size of each square qualitatively reflects the relative abundance of the corresponding neuronal subpopulation in the control group within the analyzed gut region or plexus. Fold-change values are annotated within each square. Abbreviations: *MP*, myenteric plexus; *OSP*, outer submucosal plexus; *ISP*, inner submucosal plexus; *CART*, cocaine- and amphetamine-regulated transcript; *CGRP*, calcitonin gene-related peptide; *GAL*, galanin; *SbP*, substance P; *VAChT*, vesicular acetylcholine transporter; *VIP*, vasoactive intestinal peptide; *nNOS*, neuronal nitric oxide synthase.


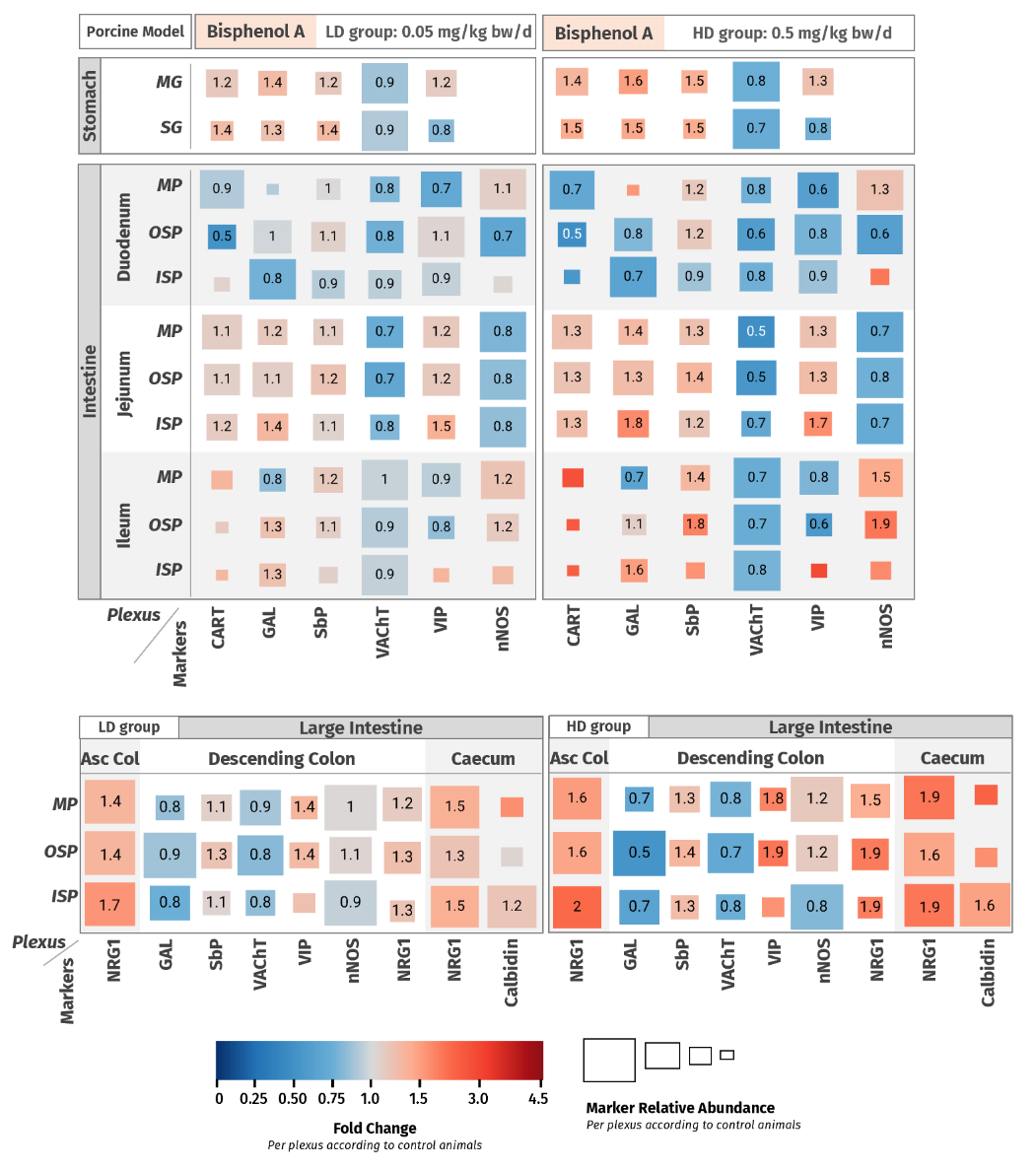


**Figure S6.** Square heatmap matrix showing the effect of bisphenol A exposure on the immunoreactivity of intestinal neuronal subpopulations in a porcine model following 28-day exposure to a low-dose (LD, 0.05 mg/kg bw/day) and high-dose (HD, 0.5 mg/kg bw/day). The heatmap displays fold-change values in neuronal marker immunoreactivity relative to untreated control animals. Color hue represents the direction and magnitude of change (red indicates upregulation, blue indicates downregulation), while the size of each square qualitatively reflects the relative abundance of the corresponding neuronal subpopulation in the control group within the analyzed gut region or plexus. Fold-change values are annotated within each square. Abbreviations: *MP*, myenteric plexus; *OSP*, outer submucosal plexus; *ISP*, inner submucosal plexus; *CART*, cocaine- and amphetamine-regulated transcript; *CGRP*, calcitonin gene-related peptide; *GAL*, galanin; *SbP*, substance P; *VAChT*, vesicular acetylcholine transporter; *VIP*, vasoactive intestinal peptide; *NRG1*, neuregulin-1; *nNOS*, neuronal nitric oxide synthase.
